# Membrane binding controls the ATPase cycle and localization of MinD in *Bacillus subtilis*

**DOI:** 10.7554/eLife.101517

**Published:** 2026-06-08

**Authors:** Helge Feddersen, Charlotte Dyckmans, Marc Bramkamp

**Affiliations:** 1 Institute for General Microbiology, Christian-Albrechts-University Kiel Kiel Germany; Washington University in St. Louis United States; University of Geneva Switzerland

**Keywords:** Min system, cell division, membrane binding, ATPase, protein patterns, SMLM, *B. subtilis*

## Abstract

Bacteria precisely regulate the place and timing of their cell division. One of the best-understood systems for division site selection is the Min system in *Escherichia coli*. In *E. coli*, the Min system displays remarkable pole-to-pole oscillation, creating a time-averaged minimum at the cell’s geometric center, which marks the future division site. Interestingly, the Gram-positive model species *Bacillus subtilis* also encodes homologous proteins: the cell division inhibitor MinC and the Walker-ATPase MinD. However, *B. subtilis* lacks the activating protein MinE, which is essential for Min dynamics in *E. coli*. We have shown before that the *B. subtilis* Min system is highly dynamic and quickly relocalizes to active sites of division. This raised questions about how Min protein dynamics are regulated on a molecular level in *B. subtilis*. Here, we show with a combination of in vitro experiments and in vivo single-molecule imaging that the ATPase activity of *B. subtilis* MinD is activated by membrane binding. Additionally, both monomeric and dimeric MinD bind to the membrane, and binding of ATP to MinD is a prerequisite for fast membrane detachment. Single-molecule localization microscopy data confirm membrane binding of monomeric MinD variants. However, only wild-type MinD enriches at cell poles and sites of ongoing division, likely due to interaction with MinJ. Monomeric MinD variants and locked dimers remain distributed along the membrane and lack the characteristic pattern formation. Single-molecule tracking data further support that MinD has a freely diffusive population, which is increased in the monomeric variants and a membrane-binding defective mutant. Thus, MinD dynamics in *B. subtilis* under the tested conditions do not require any unknown protein component and can be fully explained by MinD’s binding and unbinding kinetics with the membrane. The spatial organization of MinD relies on the short-lived temporal residence of MinD dimers at the membrane.

## Introduction

Accurate selection of the division site is essential in bacteria for maintaining cell size, ensuring proper chromosome segregation, and preventing the formation of anucleate minicells. Cell division is governed by a complex protein machine known as the divisome (Cameron and Margolin, 2024; Mahone and Goley, 2020; den Blaauwen et al., 2017). The spatiotemporal organization of this protein complex is directed by the dynamic arrangement of a bacterial tubulin homolog, FtsZ (Bi and Lutkenhaus, 1991; Löwe and Amos, 1998). FtsZ forms treadmilling protofilaments that condense into a ring-like structure at the division site (Schäper et al., 2024; Whitley et al., 2021; Squyres et al., 2021), recruiting several cell wall synthesis proteins, such as division-specific transglycosylases and transpeptidases (Egan et al., 2020; Adams and Errington, 2009). Together, these proteins synthesize the bacterial peptidoglycan cell wall. To ensure that FtsZ polymerizes only at the correct location, many bacterial species employ the Min system, a conserved pathway that prevents aberrant polar septation events.

The Min system was identified through the formation of small, round, anucleate cells that divide close to the cell poles (Adler et al., 1967). The gene locus responsible for the ‘*min*iature’ cell phenotype in *Escherichia coli* was termed *minB* (Schaumberg and Kuempel, 1983; Davie et al., 1984), later shown to encode the three structural genes *minC*, *minD,* and *minE* (de Boer et al., 1989). MinC is the actual inhibitor of FtsZ-polymer formation (de Boer et al., 1992; Hu et al., 1999). The MinC protein contains an N- and a C-terminal domain (Cordell et al., 2001), both of which contribute to FtsZ inhibition. The C-terminal domain binds to the C-terminal tail of FtsZ, whereas the N-terminal domain, which also mediates MinC dimerization, binds at the interface of two FtsZ subunits (Hu and Lutkenhaus, 2000; Shen and Lutkenhaus, 2009; Shen and Lutkenhaus, 2010). MinC-mediated inhibition of FtsZ has also been demonstrated in *Bacillus subtilis* (Marston and Errington, 1999; Gregory et al., 2008; Scheffers, 2008), suggesting a conserved mechanism across species.

The spatial localization of MinC is regulated by MinD, a Walker-type ATPase that belongs to the ParA/MinD family of proteins (Marston and Errington, 1999; de Boer et al., 1991; Marston et al., 1998). These partitioning ATPases are involved in positioning cargo within bacterial cells (Pulianmackal and Vecchiarelli, 2024; Lutkenhaus, 2012). While the molecular details of partitioning differ among ParA-like proteins, a common feature is their use of binding to a large intracellular surface as a spatial template (Pulianmackal and Vecchiarelli, 2024; Lutkenhaus, 2012). ParA proteins typically bind nonspecifically to DNA, covering the nucleoid and exploiting it as a track for cargo positioning (Szardenings et al., 2011). In contrast, MinD proteins bind to the membrane via an amphipathic helix (Szeto et al., 2003; Szeto et al., 2002). In *E. coli*, membrane binding of MinD strictly depends on ATP binding and subsequent dimerization (de Boer et al., 1991). Dimerized MinD recruits MinC and thereby activates MinC to inhibit FtsZ oligomerization. In *E. coli*, MinE further regulates MinD localization by binding to the membrane and interacting with MinD, stimulating ATP hydrolysis and triggering MinD release from the membrane. Slow nucleotide exchange in cytosolic MinD leads to an oscillatory cycle of membrane attachment and detachment, resulting in pole-to-pole oscillation of MinCD (Raskin and de Boer, 1999; Hale et al., 2001; Hu and Lutkenhaus, 1999). This oscillatory behavior generates a temporal minimum of MinC at midcell, such that Z-ring assembly is restricted exclusively to this position. The oscillation can be reconstituted in vitro on planar membranes, demonstrating the robustness of this self-organizing system and a prime example of pattern formation (Loose et al., 2008).

The Gram-positive model bacterium *B. subtilis* likewise encodes the *minC* and *minD* genes (Varley and Stewart, 1992; Levin et al., 1992), and loss of either results in the characteristic minicell phenotype. Thus, a role for the Min system analogous to that in *E. coli* has been proposed (Marston and Errington, 1999; Lee and Price, 1993). However, *B. subtilis* lacks a MinE homolog, and the Min proteins do not oscillate from pole to pole (Marston and Errington, 1999; Lee and Price, 1993). Instead, static imaging shows that MinD accumulates at the cell poles and at active division sites. Initial genetic and cell biological studies demonstrated that the polar scaffold protein DivIVA is essential for polar MinD localization (Marston and Errington, 1999). The interaction between DivIVA and MinD is mediated by the integral membrane protein MinJ (Patrick and Kearns, 2008; Bramkamp et al., 2008). MinJ is also part of the divisome and interacts with multiple division proteins (Bramkamp et al., 2008). Loss of MinJ causes defects in divisome disassembly, as proteins such as FtsL and PBP2b remain associated with the nascent cell pole after division in Δ*minJ* cells (van Baarle and Bramkamp, 2010). This function is supported by recent findings that MinD promotes FtsZ polymer disassembly at the cell poles, and that deletion of *minD* inhibits polar peptidoglycan remodeling (Yu et al., 2020).

We have recently shown that all components of the *B. subtilis* Min system, including DivIVA and MinJ, dynamically localize within the bacterial cell (van Baarle and Bramkamp, 2010; Bach et al., 2014; Giacomelli et al., 2022; Feddersen et al., 2021). Interestingly, even the scaffold protein DivIVA, although highly conserved in many Gram-positive phyla, is not statically anchored at the poles but instead redistributes along the membrane from poles to septa (Bach et al., 2014; Giacomelli et al., 2022; Feddersen et al., 2021). In contrast, DivIVA homologs in actinobacteria show greatly reduced mobility, consistent with their role in apical growth (Giacomelli et al., 2022).

Despite these advances, the mechanism by which MinD activity and localization are regulated in the absence of MinE has remained unclear, raising the possibility of additional, unidentified regulatory factors or alternative modes of ATPase control. In this study, we address these long-standing questions by combining in vitro biochemical analyses with single-molecule imaging in vivo. We define the ATPase and membrane-binding properties of *B. subtilis* MinD and examine how its dynamics are shaped by interactions with MinJ and MinC. Our findings reveal an unexpected mechanism of MinD regulation that operates independently of MinE-like factors, providing a revised understanding of how the non-oscillatory *B. subtilis* Min system robustly controls cytokinesis and prevents aberrant septation events.

## Results

### Purification and gel filtration of His-MinD

Since localization and function of MinD appear to be strongly tied to its ATPase activity, the first logical step in describing and understanding details of the ATPase cycle was identification of the relevant biochemical characteristics, to eventually be able to correlate these properties to in vivo observations. To this end, *B. subtilis minD* was heterologously expressed in *E. coli*, yielding an N-terminally His-tagged fusion protein (Figure 1—figure supplement 1), as C-terminal MinD fusions have been shown to interfere with membrane binding (Ishikawa et al., 2017). Since MinD is known for being membrane-associated, a well-established *E. coli* MinD purification protocol was adapted (Ramm et al., 2018), where buffers were supplied with excess Mg^2+^-ADP to potentially reduce MinD self- and membrane interaction. Based on the respective western blot and gel filtration chromatogram (Figure 1—figure supplements 1 and 2), His-MinD is not able to dimerize when purified in the presence of excess Mg^2+^-ADP, as evidenced by a main peak at the expected sizes of the monomeric form (~31 kDa, 16.11 ml) and the absence of a peak at the expected size of the dimeric form (~62 kDa, 14.65 ml, Figure 1—figure supplement 2). However, western blotting of Ni-NTA and size-exclusion chromatography (SEC) fractions indicates the presence of multiple His-tagged degradation products alongside full-length His-MinD (Figure 1—figure supplement 1). Because these fragments retain the His-tag, they co-purify during Ni-NTA and can co-elute or form non-covalent assemblies with full-length MinD, increasing the heterogeneity of the sample and likely producing the minor shoulder observed at an apparent molecular weight of ~44 kDa (15.4 ml, Figure 1—figure supplement 2). Unfortunately, the Mg^2+^-ADP excess in the purification buffers did not seem to stop His-MinD membrane interaction, as a significant fraction of the recombinant protein was later detected to be lost in the cell pellet. However, sufficiently high His-MinD concentrations could be obtained to proceed with biochemical analysis.

### *B. subtilis* MinD displays full ATPase activity when incubated with ATP and liposomes

**Figure 1.**
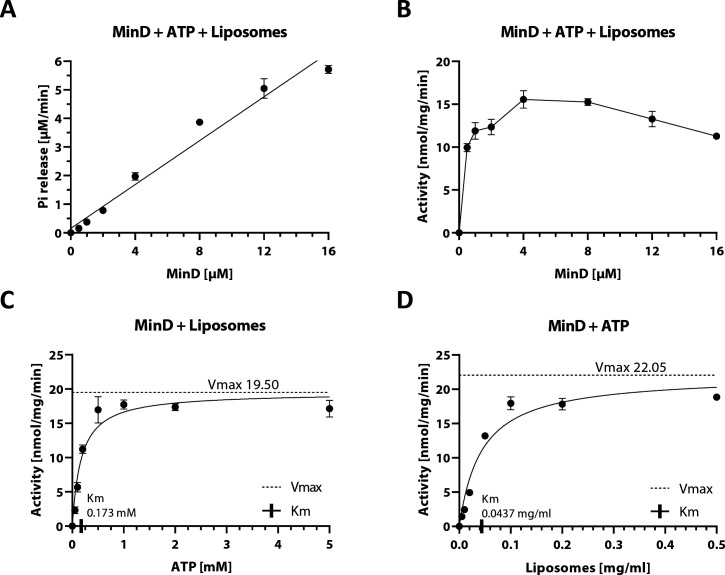
Biochemical analysis of *B*. *subtilis* MinD ATPase cycle. (**A**) Phosphate release plotted against different MinD concentrations, fitted with a simple linear regression (*R*^2^=0.97). Phosphate release was measured using the EnzChek phosphate assay kit. Samples contained 0.2 mg ml^–1^ liposomes and were pre-incubated for 10 min before addition of 2 mM Mg^2+^-ATP; *n*≥3. (**B**) Specific activity of the MinD ATPase from (**A**) measured as a function of MinD concentration. (**C**) Specific activity of MinD measured as a function of ATP concentration, determined as in (**B**) with fixed MinD concentration of 10 µM. Fitting the Michaelis-Menten equation (black lines) gives *k*_cat_ = 36.27 hr^−1^, *V*_max_ = 19.50 nmol mg^–1^ min^–1^ and *K*_M_ = 0.173 mM. (**D**) Specific activity of MinD measured as a function of liposome concentration, determined as in (**B**) with fixed MinD concentration of 6 µM. Fitting the Michaelis-Menten equation (black lines) gives *k*_cat_ = 41.01 hr^−1^, *V*_max_ = 22.05 nmol mg^–1^ min^–1^ and *K*_M_ = 0.0437 mg ml^–1^.

Being able to purify His-MinD allowed us to investigate the biochemical conditions affecting the MinD ATPase cycle next. For this purpose, we chose to use the commercially available enzyme-coupled EnzChek phosphate assay kit (Thermo Fisher Scientific) to measure ATPase activity. The activity was determined by measuring the P_i_ production rate (in µM min^−1^) as a function of His-MinD concentration. We first tested His-MinD ATPase activity at protein concentrations between 0.5 µM and 16 µM. Under these conditions, only baseline ATPase activity below the detection limit was observed (consistent with Figures 1D, 0 mg ml^–1^ liposomes condition). Since MinD interacts with the membrane and it was expected that the ATPase cycle is involved in controlling this interaction, we next measured ATPase activity in the presence of liposomes extruded from *E. coli* total lipids (Avanti). Remarkably, this led to a robust increase in ATPase activity of His-MinD, appearing to be linearly dependent on protein concentration (Figure 1A and B). To be able to compare the activity level to the published *E. coli* MinD in vitro ATPase data (Hu and Lutkenhaus, 2001; Hu and Lutkenhaus, 2003; Heermann et al., 2020), the specific activity is shown as a specific activity in nmol mg^–1^ min^–1^ (Figure 1B). Interestingly, activity levels of recombinant *B. subtilis* MinD were found to be on par with fully active *E. coli* MinD in conjunction with MinE and phospholipids (Hu and Lutkenhaus, 2001). Moreover, the ATPase activity did not show cooperative behavior compared to *E. coli* MinD at lower concentrations (Hu and Lutkenhaus, 2001). We further measured that ATP hydrolysis can be titrated with both ATP and liposomes and is described by Michaelis-Menten kinetics (Figure 1C and D). Again, the *V*_max_ values obtained here (19.50 nmol mg^–1^ min^–1^ and 22.05 nmol mg^–1^ min^–1^, respectively) indicate similar ATPase activity levels for MinD from *E. coli* and *B. subtilis*.

**Figure 1—figure supplement 1.**
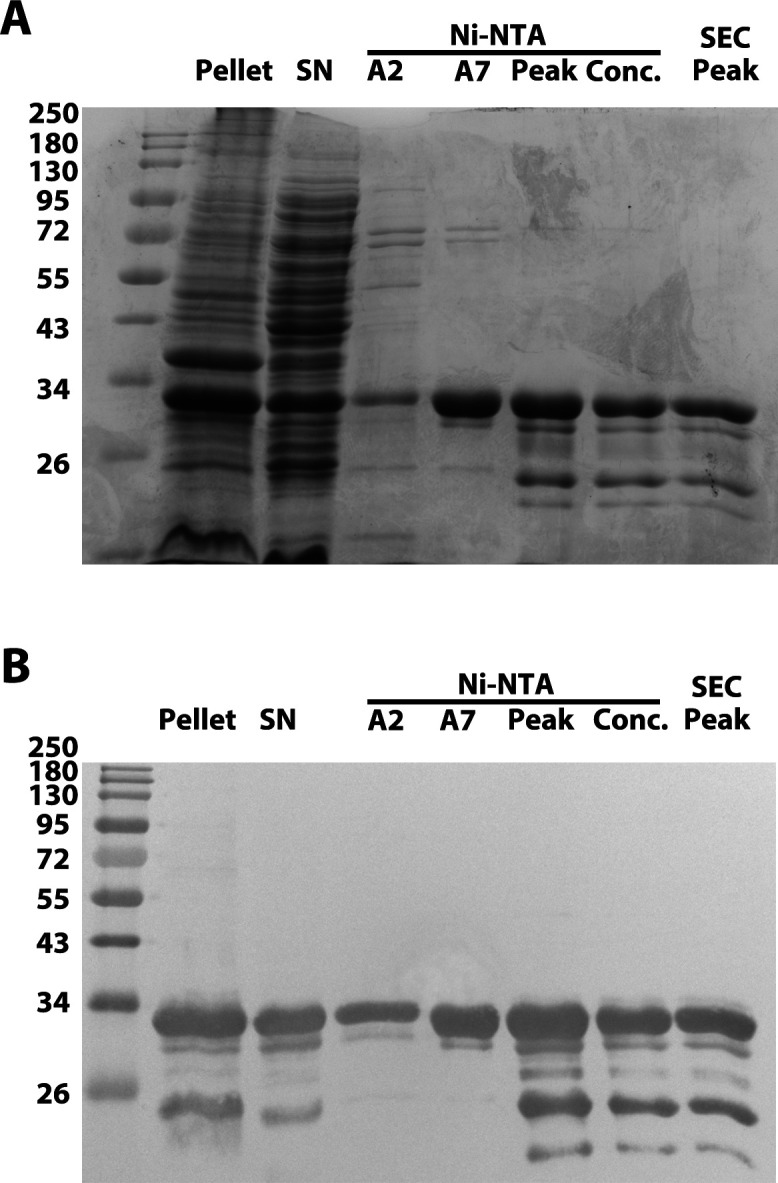
His-MinD purification analysis. (**A**) Sodium dodecyl sulfate-polyacrylamide gel electrophoresis (SDS-PAGE) gel analysis of purification products of His-MinD (31.7 kDa), stained with Coomassie blue stain. From left to right: Ladder, pellet after lysate, supernatant, Ni-NTA peaks A2, A7, C4 (main peak), concentrated sample, gel filtration peak. Ladder = Color Prestained Protein Standard, Broad Range (New England Biolabs). (**B**) Western blot of replicate gel from (**A**) using Penta-His antibody (QIAGEN). Figure 1—figure supplement 1—source data 1.Original files for sodium dodecyl sulfate-polyacrylamide gel electrophoresis (SDS-PAGE) and western blot analysis displayed in Figure 1—figure supplement 1. Figure 1—figure supplement 1—source data 2.PDF file containing original sodium dodecyl sulfate-polyacrylamide gel electrophoresis (SDS-PAGE) and western blots for Figure 1—figure supplement 1, indicating the relevant bands.

**Figure 1—figure supplement 2.**
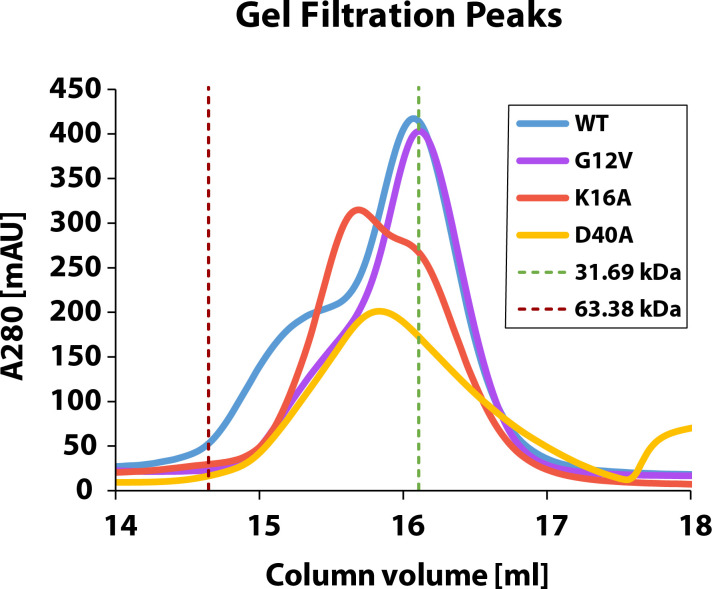
Gel filtration chromatograms of His-MinD and variants. Gel filtration analysis of purified His-MinD and indicated mutants. Curves were aligned according to the respective void volume after sample injection. Green and red dotted lines mark the calibrated elution volumes corresponding to proteins of 31.69 kDa (His-MinD monomer) and 63.38 kDa (His-MinD dimer), as determined from molecular weight standards.

### MinC and the PDZ domain of MinJ have different effects on MinD ATPase activity

Even though ATPase activity levels were similar, proteins that directly interact with MinD could potentially modulate this activity in vivo as seen for MinD:MinE in *E. coli* (Hu and Lutkenhaus, 2001; Hu and Lutkenhaus, 2003; Heermann et al., 2020). To get the complete picture of MinD activity, investigating the potential effects of both known *B. subtilis* MinD interactors MinC and MinJ was therefore necessary. For this purpose, both proteins were subjected to purification attempts. While purification of the cytosolic His-MinC could be successfully established using affinity chromatography followed by SEC (Figure 2—figure supplement 1), the intermembrane protein His-MinJ proved more challenging. Since several different purification attempts of the full-length protein yielded no stable product, a truncated form containing only the soluble C-terminal PDZ domain of MinJ fused to an N-terminal His-tag (His-PDZ) via a flexible linker was expressed instead (Figure 2—figure supplement 2). PDZ domains are often found in membrane-associated proteins and known to selectively interact with the C-terminus of partner peptides or proteins to sequester them to highly specific sites of the cell, in both pro- and eukaryotes (Ponting et al., 1997). Although His-PDZ could be isolated via Ni-NTA affinity chromatography, it was unstable during SEC and consistently lost from the column, precluding further purification by this method. Hence, further experiments were performed using His-PDZ directly after affinity chromatography.

**Figure 2.**
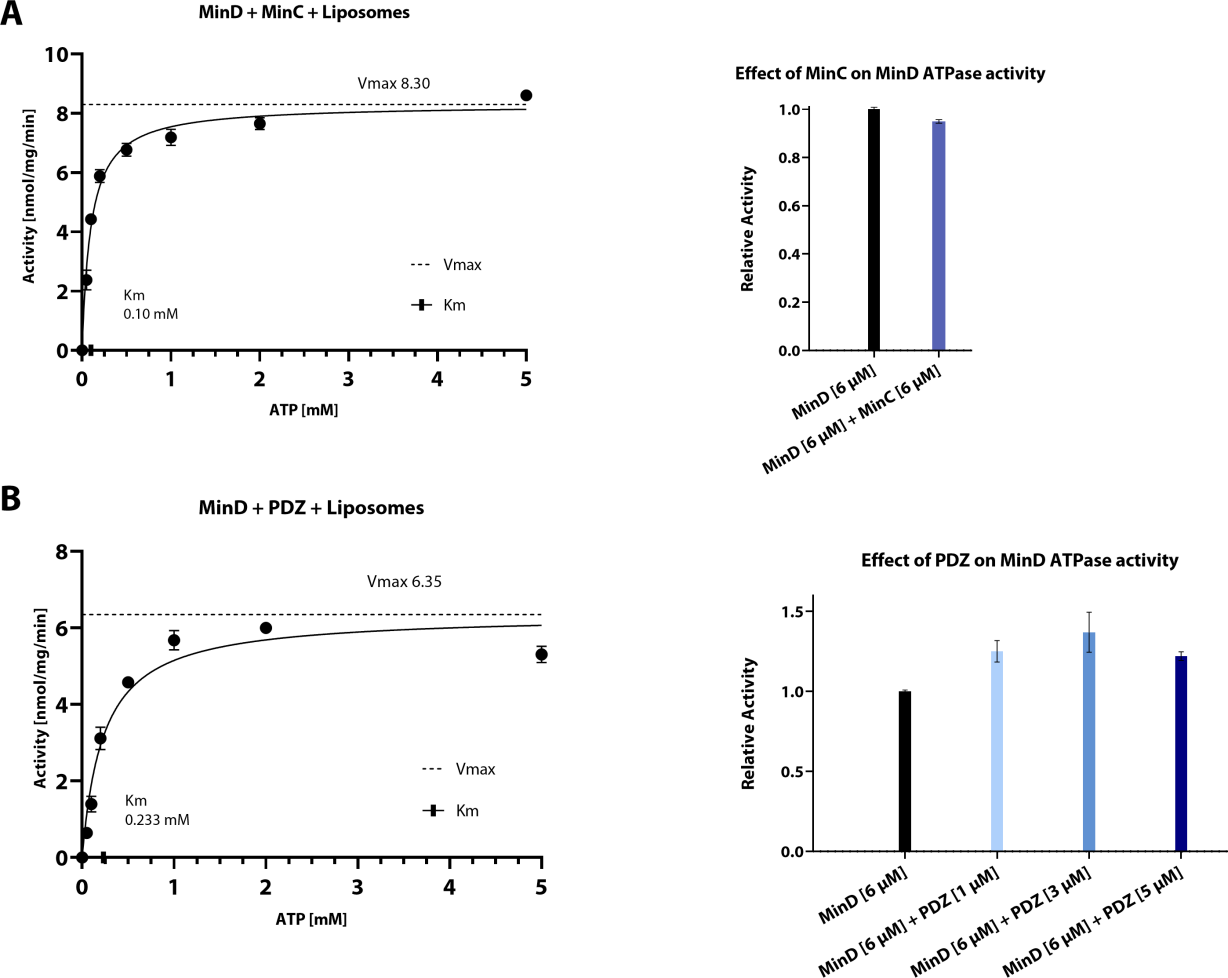
Biochemical analysis of the effect of His-MinC or His-PDZ on MinD ATPase activity. (**A**) Left: Specific activity of MinD measured as a function of ATP concentration. Phosphate release was measured using the EnzChek phosphate assay kit. Samples contained 6 µM His-MinD, 6 µM His-MinC, and 0.2 mg ml^–1^ liposomes and were pre-incubated for 10 min before addition of 2 mM Mg^2+^-ATP; *n*≥3. Fitting the Michaelis-Menten equation (black lines) gives *k*_cat_ = 15.77 hr^−1^, *V*_max_ = 8.30 nmol mg^–1^ min^–1^ and *K*_M_ = 0.10 mM. Right: Relative activity of 6 µM His-MinD in the presence of 6 µM His-MinC (blue) compared to an internal control of 6 µM His-MinD (black, relative activity = 1). Whiskers represent SD. Specific activity of only His-MinD was 8.61 [nmol mg^–1^ min^–1^] (SD = 0.03, *n*=2). Relative activity of 6 µM His-MinD in the presence of 6 µM His-MinC was 0.95 with SD = 0.071 (*n*=3). Significance was assessed by performing Welch’s t-test on the raw data. p<0.05. (**B**) Left: Specific activity of MinD measured as a function of ATP concentration. Phosphate release was measured using the EnzChek phosphate assay kit. Samples contained 6 µM His-MinD, 5 µM His-PDZ, and 0.2 mg ml^–1^ liposomes and were pre-incubated for 10 min before addition of 2 mM Mg^2+^-ATP; *n*≥3. Fitting the Michaelis-Menten equation (black lines) gives *k*_cat_ = 12.07 hr^−1^, *V*_max_ = 6.35 nmol mg^–1^ min^–1^ and *K*_M_ = 0.233 mM. Right: Relative activity of 6 μM His-MinD in the presence of different concentrations of His-PDZ (blue) compared to only 6 μM His-MinD (black, relative activity = 1). Whiskers represent SD. Specific activity of an internal control of His-MinD was 4.60 nmol mg^–1^ min^–1^ (SD = 0.03, *n*=2). Relative activities of His-MinD in the presence of 1 µM, 3 µM, and 5 µM His-PDZ were 1.22, 1.37, and 1.25 with SD = 0.07, 0.13, and 0.03, respectively (*n*=3 each). Significance was assessed by performing Welch’s t-test on the raw data of all samples vs. control. p<0.05 for all PDZ concentrations.

To test the effect of His-MinC and His-PDZ on His-MinD ATPase activity, the previously established enzymatic assays were repeated in the presence of either His-MinC or His-PDZ (Figure 2). Addition of His-MinC resulted in a very small but statistically significant decrease in MinD ATPase activity (~5%). Given the small sample size, this effect should be interpreted cautiously. However, it could be caused by the experimental setup, since the high amounts of MinC (6 µM) that have been used might sequester a small fraction of MinD away from membrane surfaces that are required for efficient ATP hydrolysis, due to direct interaction of MinC and MinD.

**Figure 2—figure supplement 1.**
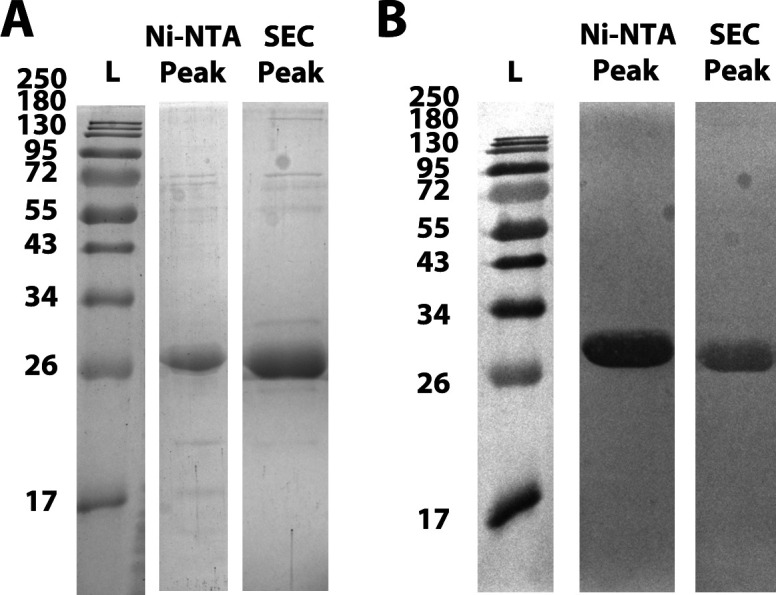
His-MinC purification analysis. (**A**) Sodium dodecyl sulfate-polyacrylamide gel electrophoresis (SDS-PAGE) gel analysis of purification products of His-MinC (27.3 kDa), stained with Coomassie blue stain. From left to right: Ladder, Ni-NTA peak, SEC peak. Ladder = Color Prestained Protein Standard, Broad Range (New England Biolabs). (**B**) Western blot of replicate gels from (**A**) using Penta-His antibody (QIAGEN). Figure 2—figure supplement 1—source data 1.Original files for sodium dodecyl sulfate-polyacrylamide gel electrophoresis (SDS-PAGE) and western blot analysis displayed in Figure 2—figure supplement 1. Figure 2—figure supplement 1—source data 2.PDF file containing original sodium dodecyl sulfate-polyacrylamide gel electrophoresis (SDS-PAGE) and western blots for Figure 2—figure supplement 1, indicating the relevant bands.

**Figure 2—figure supplement 2.**
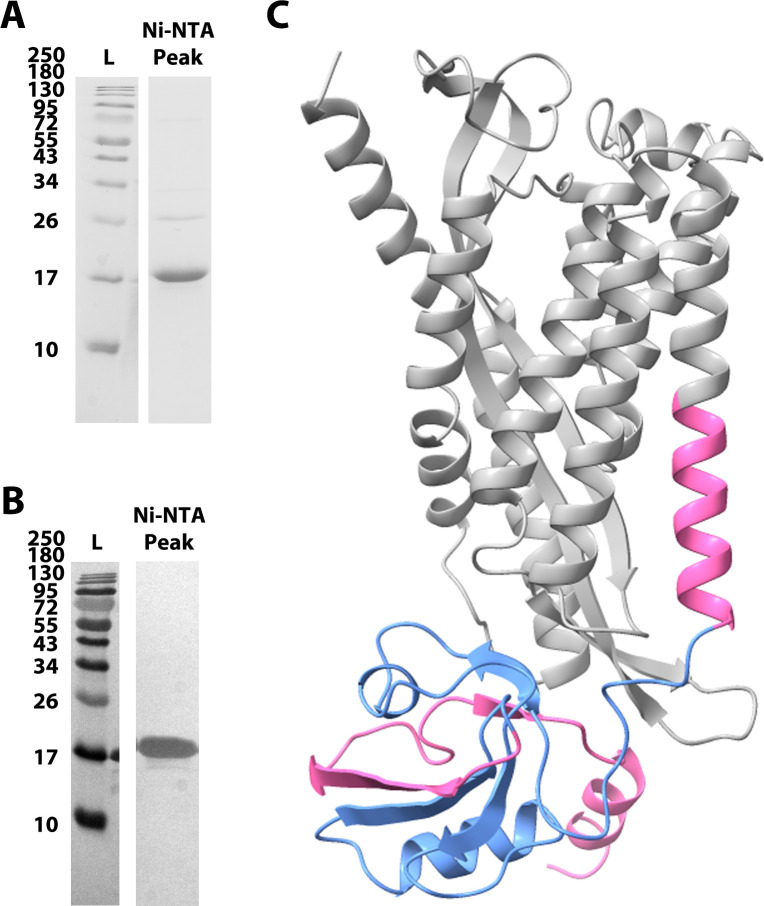
His-PDZ purification analysis and rendering of MinJ AlphaFold model. (**A**) Sodium dodecyl sulfate-polyacrylamide gel electrophoresis (SDS-PAGE) gel analysis of purification products of His-PDZ (17.6 kDa), stained with Coomassie blue stain. From left to right: Ladder (**L**), Ni-NTA main peak. Ladder = Color Prestained Protein Standard, Broad Range (New England Biolabs). (**B**) Western blot of replicate gel from (**A**) using Penta-His antibody (QIAGEN). (**C**) Rendering of AlphaFold3 model of *B. subtilis* MinJ (gray) (Abramson et al., 2024). The truncation expressed with pET-28a-PDZ, which includes the PDZ domain (blue), is highlighted in pink. Figure 2—figure supplement 2—source data 1.Original files for sodium dodecyl sulfate-polyacrylamide gel electrophoresis (SDS-PAGE) and western blot analysis displayed in Figure 2—figure supplement 2. Figure 2—figure supplement 2—source data 2.PDF file containing original sodium dodecyl sulfate-polyacrylamide gel electrophoresis (SDS-PAGE) and western blots for Figure 2—figure supplement 2, indicating the relevant bands.

In contrast, addition of His-PDZ led to a slight but reproducible increase in MinD ATPase activity, which prompted us to repeat the experiment at different His-PDZ concentrations (Figure 2). All His-PDZ concentrations significantly increased His-MinD ATPase activity compared to the control (1 µM His-PDZ=25% ± 6.8%, 3 µM His-PDZ=37 ± 12.5%; 5 µM His-PDZ=22% ± 2.8%). We speculate that the PDZ domain may promote MinD:MinD encounters, facilitating dimerization rather than directly enhancing catalytic turnover. Because only the soluble PDZ domain could be purified, it remains unclear how full-length, membrane-anchored MinJ would influence MinD activity in vivo. The magnitude of the effect might differ when the PDZ domain is present in its native membrane-associated context.

### MinD ATPase activity is triggered by membrane binding

**Figure 3.**
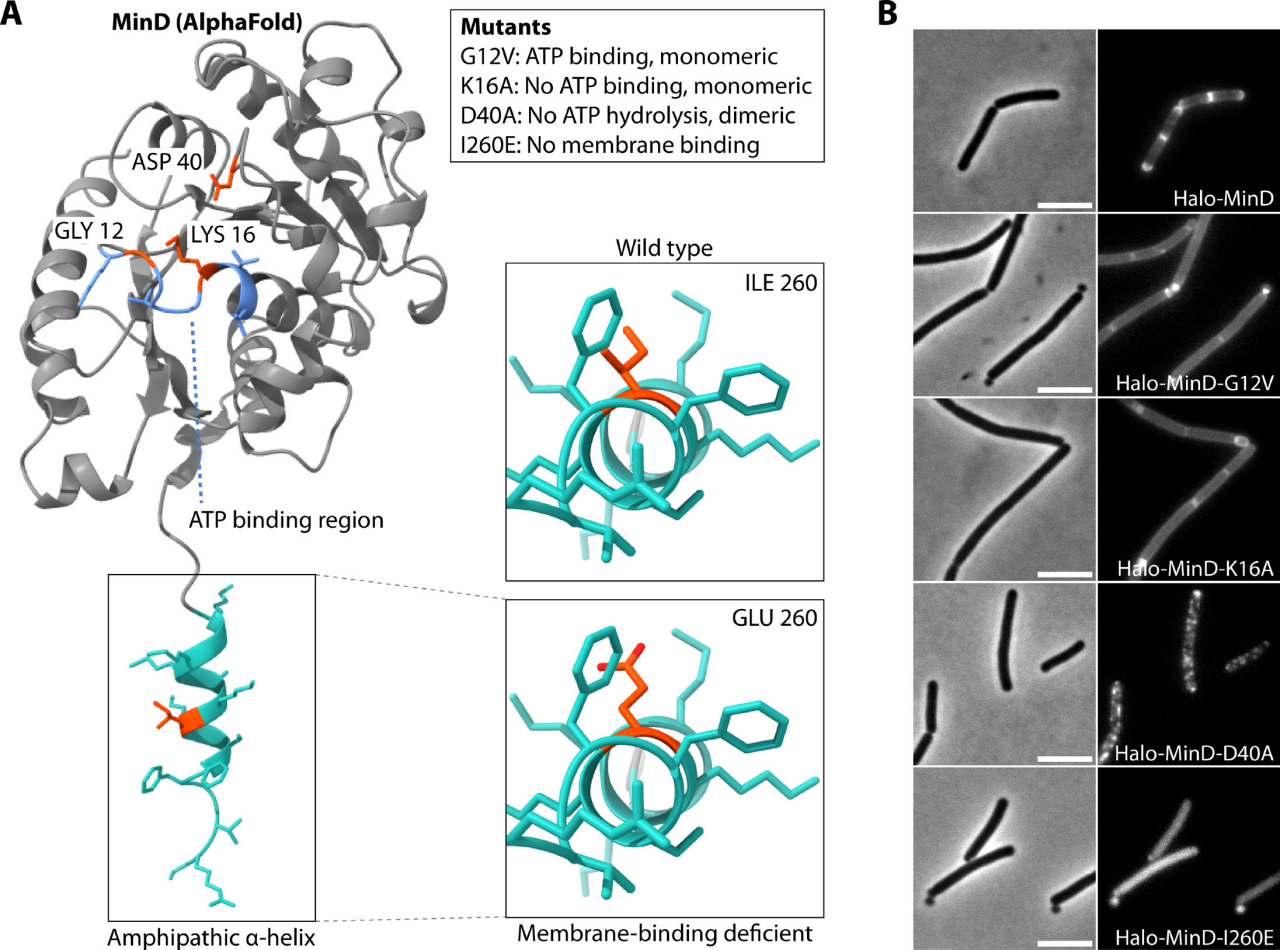
Cartoon of MinD AlphaFold model with highlighted key amino acids and the effects of their mutagenesis. (**A**) Left: Rendering of MinD AlphaFold model with ATP binding region highlighted in light blue, membrane targeting sequence (MTS) in turquoise, and key residues in orange. Box: explanation of mutagenesis effects. Right: Close-up on hydrophobic region of amphipathic helix. (**B**) Representative wide-field microscopy images of *B. subtilis* expressing indicated Halo-MinD variants as the only MinD copy from the native locus, stained with TMR ligand. Left: phase contrast; scale bar 5 µm. Right: fluorescence.

To determine whether interaction with the membrane is sufficient to stimulate MinD ATPase activity, a MinD mutant that is defective in membrane binding was required. MinD interacts with the membrane through an amphipathic α-helix located at the N-terminus, thus being the membrane targeting sequence (MTS) (Figure 3A). Exchanging the hydrophobic isoleucine residue in the apolar core of the MTS with a charged glutamate (I260E) has been shown to abolish membrane binding of MinD by perturbing the amphipathic properties, demonstrated through fluorescence microscopy (Strahl and Hamoen, 2010). Accordingly, a His-MinD-I260E variant was constructed and purified via the established purification protocol. The yield of the purification was extensively higher compared to the wild-type protein (Figure 4—figure supplement 1), hinting toward a successful abolishment of membrane interaction.

**Figure 4.**
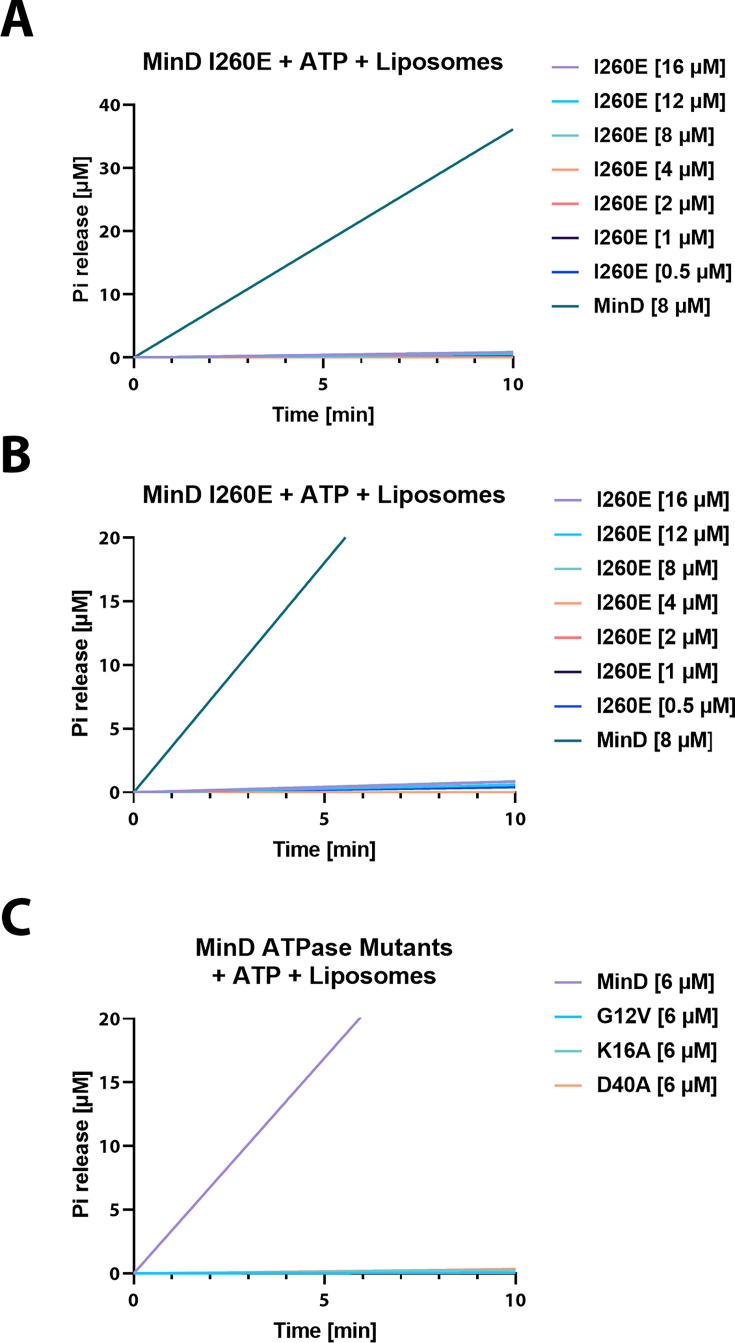
His-MinD mutants are catalytically inactive in ATP hydrolysis assay. (**A**) Phosphate release plotted against different His-MinD-I260E concentrations and His-MinD as control, fitted with a simple linear regression. Phosphate release was measured using the EnzChek phosphate assay kit. Samples contain 0.2 mg ml^–1^ liposomes and were pre-incubated for 10 min before the addition of 2 mM Mg^2+^-ATP. (**B**) Same as (**A**) but with different y-axis scaling for better visualization of His-MinD-I260E baseline activity. (**C**) Same as (**B**) but comparing His-MinD mutants G12V, K16A, and D40A to His-MinD at 6 µM concentrations, respectively.

We next repeated ATPase activity assays with the purified recombinant I260E mutant and, remarkably, its activity remained below the detection limit of the assay, while a positive control of His-MinD wild-type protein displayed regular activity (Figure 4). In summary, these data collectively suggest that membrane binding is both necessary and the key trigger for ATP hydrolysis of *B. subtilis* MinD, without any apparent need for an additional stimulus.

**Figure 4—figure supplement 1.**
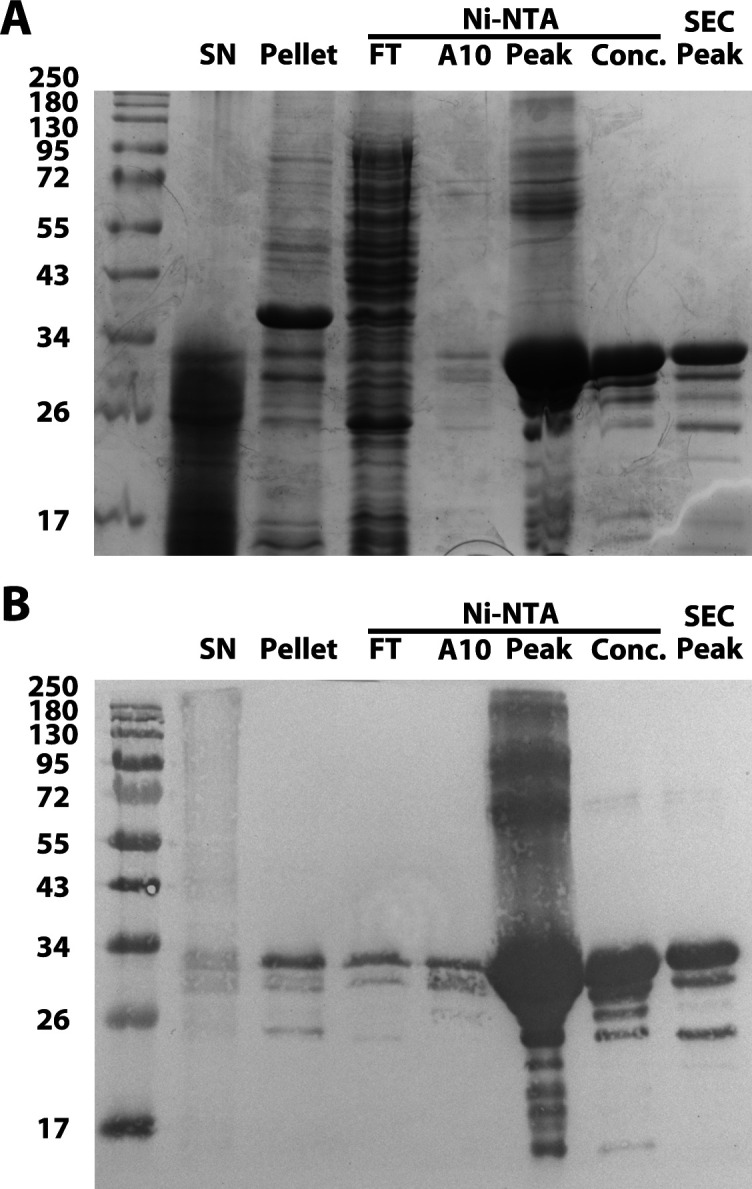
His-MinD-I260E purification analysis. (**A**) Sodium dodecyl sulfate-polyacrylamide gel electrophoresis (SDS-PAGE) gel analysis of purification products of His-MinD-I260E (31.7 kDa), stained with Coomassie blue stain. From left to right: Ladder, supernatant after lysis, pellet, Ni-NTA flowthrough, Ni-NTA peaks A10 and C4 (main peak), concentrated sample, gel filtration peak. Ladder = Color Prestained Protein Standard, Broad Range (New England Biolabs). (**B**) Western blot of replicate gel from (**A**) using Penta-His antibody (QIAGEN). Figure 4—figure supplement 1—source data 1.Original files for sodium dodecyl sulfate-polyacrylamide gel electrophoresis (SDS-PAGE) and western blot analysis displayed in Figure 4—figure supplement 1. Figure 4—figure supplement 1—source data 2.PDF file containing original sodium dodecyl sulfate-polyacrylamide gel electrophoresis (SDS-PAGE) and western blots for Figure 4—figure supplement 1, indicating the relevant bands.

**Figure 4—figure supplement 2.**
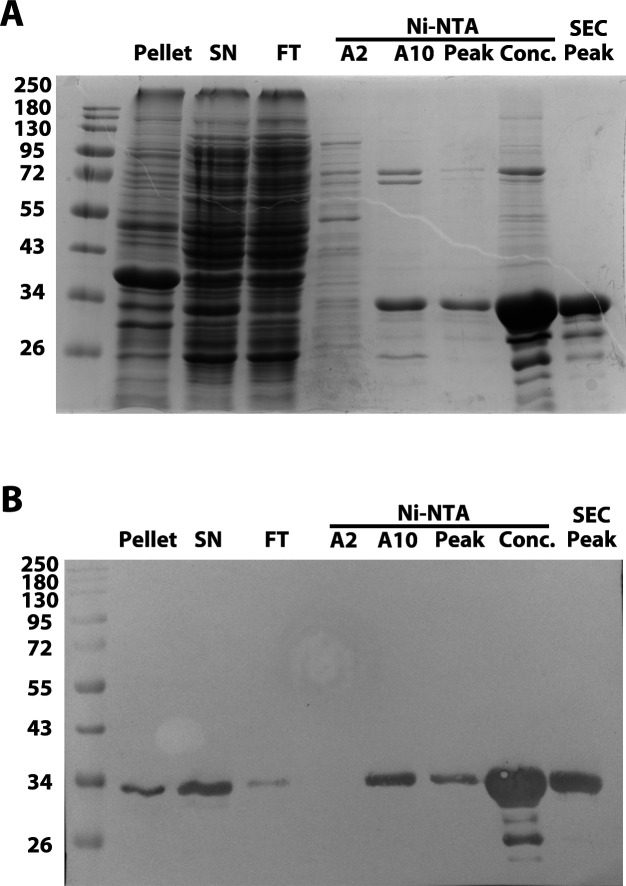
His-MinD-G12V purification analysis. (**A**) Sodium dodecyl sulfate-polyacrylamide gel electrophoresis (SDS-PAGE) gel analysis of purification products of His-MinD-G12V (31.7 kDa), stained with Coomassie blue stain. From left to right: Ladder, pellet after lysate, supernatant, Ni-NTA flowthrough, Ni-NTA peaks A2, A10, C4 (main peak), concentrated sample, gel filtration peak. Ladder = Color Prestained Protein Standard, Broad Range (New England Biolabs). (**B**) Western blot of replicate gel from (**A**) using Penta-His antibody (QIAGEN). Figure 4—figure supplement 2—source data 1.Original files for sodium dodecyl sulfate-polyacrylamide gel electrophoresis (SDS-PAGE) and western blot analysis displayed in Figure 4—figure supplement 2. Figure 4—figure supplement 2—source data 2.PDF file containing original sodium dodecyl sulfate-polyacrylamide gel electrophoresis (SDS-PAGE) and western blots for Figure 4—figure supplement 2, indicating the relevant bands.

**Figure 4—figure supplement 3.**
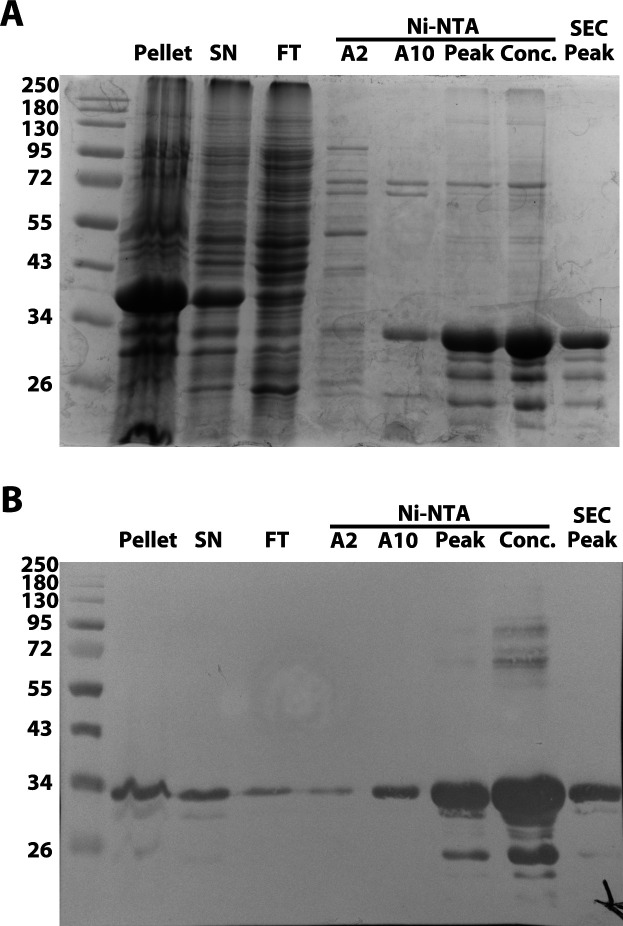
His-MinD-K16A purification analysis. (**A**) Sodium dodecyl sulfate-polyacrylamide gel electrophoresis (SDS-PAGE) gel analysis of purification products of His-MinD-K16A (31.7 kDa), stained with Coomassie blue stain. From left to right: Ladder, pellet after lysate, supernatant, Ni-NTA flowthrough, Ni-NTA peaks A2, A10, C4 (main peak), concentrated sample, gel filtration peak. Ladder = Color Prestained Protein Standard, Broad Range (New England Biolabs). (**B**) Western blot of replicate gel from (**A**) using Penta-His antibody (QIAGEN). Figure 4—figure supplement 3—source data 1.Original files for sodium dodecyl sulfate-polyacrylamide gel electrophoresis (SDS-PAGE) and western blot analysis displayed in Figure 4—figure supplement 3. Figure 4—figure supplement 3—source data 2.PDF file containing original sodium dodecyl sulfate-polyacrylamide gel electrophoresis (SDS-PAGE) and western blots for Figure 4—figure supplement 3, indicating the relevant bands.

**Figure 4—figure supplement 4.**
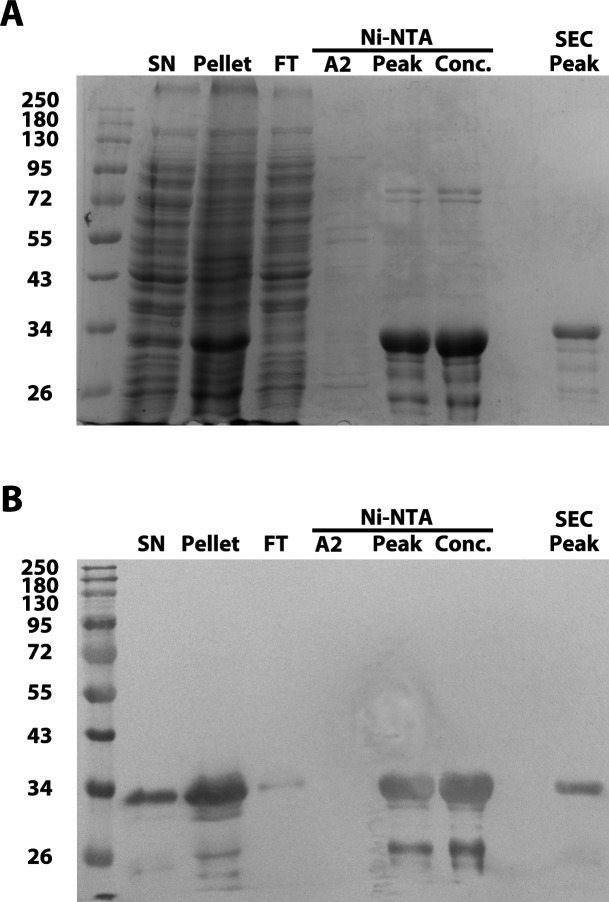
His-MinD-D40A purification analysis. (**A**) Sodium dodecyl sulfate-polyacrylamide gel electrophoresis (SDS-PAGE) gel analysis of purification products of His-MinD-D40A (31.7 kDa), stained with Coomassie blue stain. From left to right: Ladder, supernatant after lysis, pellet, Ni-NTA flowthrough, Ni-NTA peaks A2 and C4 (main peak), concentrated sample, gel filtration peak. Ladder = Color Prestained Protein Standard, Broad Range (New England Biolabs). (**B**) Western blot of replicate gel from (**A**) using Penta-His antibody (QIAGEN). Figure 4—figure supplement 4—source data 1.Original files for sodium dodecyl sulfate-polyacrylamide gel electrophoresis (SDS-PAGE) and western blot analysis displayed in Figure 4—figure supplement 4. Figure 4—figure supplement 4—source data 2.PDF file containing original sodium dodecyl sulfate-polyacrylamide gel electrophoresis (SDS-PAGE) and western blots for Figure 4—figure supplement 4, indicating the relevant bands.

Next, we wanted to compare in vivo and in vitro data. Therefore, a *B. subtilis* strain for microscopy was constructed, where the native copy of MinD was replaced by a HaloTag-MinD fusion protein (Figure 3B, HaloTag will henceforth be referred to as Halo). We then introduced the I260E mutation, resulting in a strain producing Halo-MinD-I260E as the only copy. Microscopic analysis of a Halo-MinD-I260E with TMR dye confirmed drastically reduced membrane binding through its almost exclusive cytosolic localization (Figure 3B). Furthermore, cell length and the number of minicells increased drastically, comparable to a *minD* knockout (Table 1).

**Table 1.** Measurements of cell length and minicell formation of *B. subtilis* strains.

Strain	Relevant genotype	Length ± SD (µm)	Minicells (%)	No. cells counted
**168**	**Wild type**	3.32±0.91	0.20	610
**BHF077**	**Δ*minD***	5.80±2.10	33.53	865
**BHF078**	**Halo-MinD**	4.06±0.99	7.44	430
**BHF079**	**Halo-MinD (G12V**)	6.13±2.13	34.54	443
**BHF080**	**Halo-MinD (K16A**)	5.44±1.84	38.45	489
**BHF081**	**Halo-MinD (D40A**)	5.67±2.09	33.70	454
**BHF082**	**Halo-MinD (I260E**)	5.38±1.68	33.01	721

### Construction of catalytic MinD mutants

To determine the role of ATP-mediated dimerization in membrane binding, we explored further mutations that affect the ATP binding region and thus the dimerization process. Due to the conserved nature of Walker-type MinD/ParA-like ATPases, residues involved in catalytic activity have been well characterized (Karoui and Errington, 2001; Leonard et al., 2005; Wu et al., 2011). We therefore constructed expression plasmids encoding three different MinD variants (illustrated in Figure 3A): This first mutation, MinD-G12V, produces a steric clash within the active site, so that ATP can still be bound, but dimers cannot form (Karoui and Errington, 2001; Leonard et al., 2005). The second mutation, K16A, completely disrupts ATP binding and hence results in monomeric protein (Karoui and Errington, 2001). The third variant encodes the mutation D40A, which should interfere with ATP hydrolysis (Leonard et al., 2005; Wu et al., 2011). Therefore, it was expected that MinD-D40A forms a trapped nucleotide sandwich dimer similar to Soj or *E. coli* MinD (Leonard et al., 2005; Wu et al., 2011). All MinD variants were successfully expressed and purified and resulted in similar protein yields when compared to the wild-type protein (Figure 1—figure supplement 1, Figure 4—figure supplement 2, Figure 4—figure supplements 3 and 4). When evaluating the gel filtration chromatograms, His-MinD-G12V eluted in a single peak, which can be interpreted as a monomer when compared to the wild-type His-MinD elution peak (Figure 1—figure supplement 2). His-MinD-D40A, on the other hand, also eluted in a single peak, albeit being wider and eluting earlier, indicating a mainly dimeric conformation. It eluted between the expected dimer and monomer positions during SEC as a wide peak (Figure 1—figure supplement 2). As His-MinD-D40A cannot hydrolyze ATP, it should be dimeric and likely also form small oligomers or transient multimers. A mixture of monomer/dimer/oligomer likely produced the broad, left-shifted peak whose center is between monomer and dimer. Only His-MinD-K16A reproducibly formed a rather unexpected shoulder in its main elution peak, which could hint toward further interaction with the gel filtration matrix or other potential self-interactions. All of the recombinant His-MinD mutants displayed negligible ATPase activity when tested using the EnzChek phosphate assay kit (Figure 4C). This was expected, as all of these MinD variants (G12V, K16A, and D40A) were mutated in residues that significantly alter the ATP binding site. In all cases, activity levels remained consistently near the detection limit, precluding reliable quantitative interpretation.

To investigate in vivo effects of these variants, the same mutations were introduced into the strain expressing Halo-MinD in *B. subtilis*, respectively, and subsequently imaged via wide-field microscopy (Figure 3B). All strains producing MinD mutants displayed an increased cell length and minicell production comparable to Δ*minD* (Table 1), as it was expected in cells containing dysfunctional Min proteins. Surprisingly, both monomeric MinD variants (G12V and K16A) were still observed to be membrane associated; however, resulting in the apparent loss of the polar-septal MinD localization (Figure 3B). Finally, the locked dimer D40A mutant appeared to be mainly membrane associated, thereby forming large foci, indicating accumulation of MinD, but much less dispersed when compared to G12V or K16A (Figure 3B). In summary, the time-resolved MinD gradient appears to be lost in all MinD mutants, including D40A. This suggests that the ATPase cycle and thus quick cycling of MinD is indeed necessary for the formation of the observed dynamic gradient, as it has been previously suggested (Feddersen et al., 2021).

### Monomeric MinD binds the membrane with high efficiency

**Figure 5.**
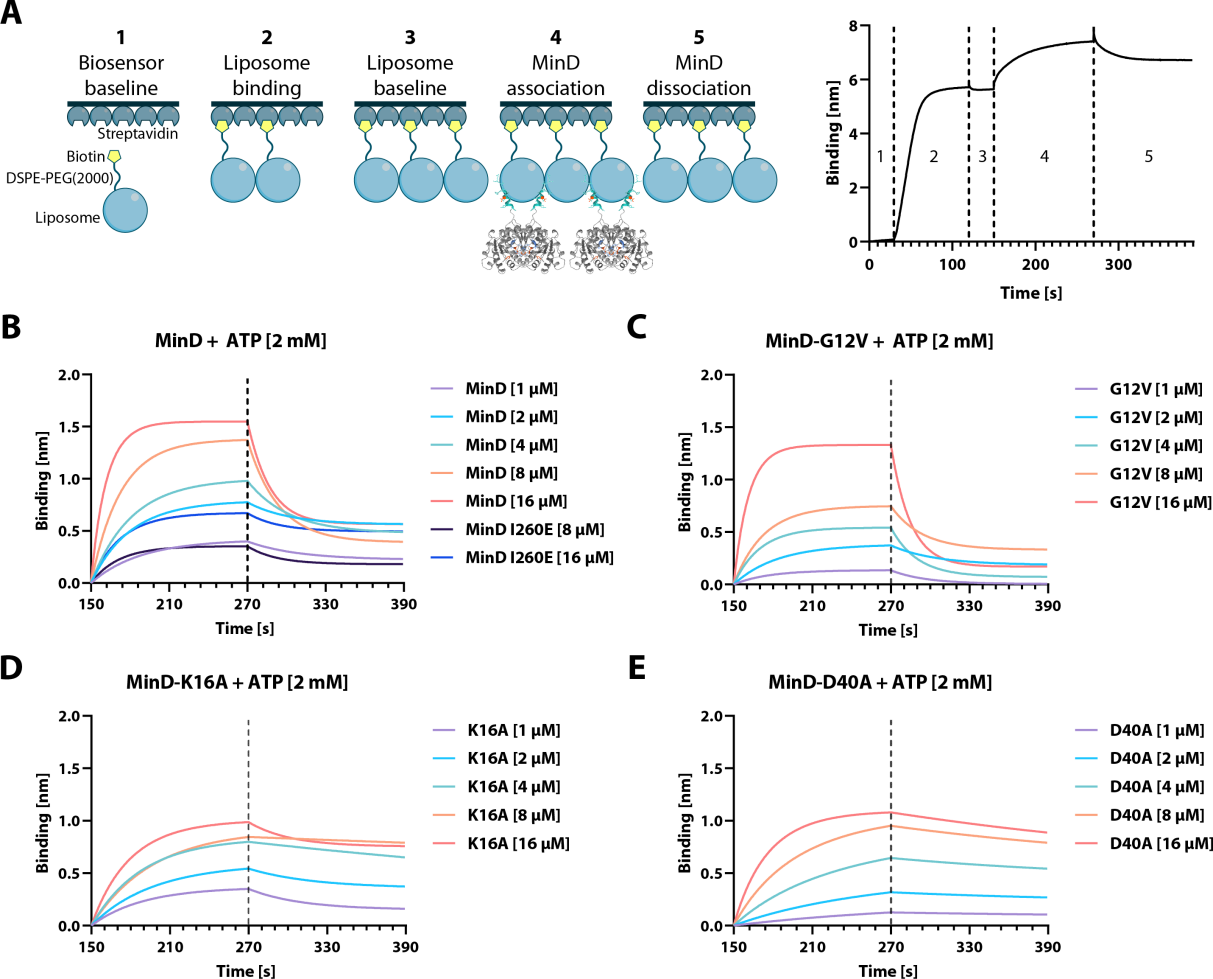
Bio-layer interferometry (BLI) analysis of His-MinD and different mutants. (**A**) Left: Cartoon representation of the different BLI steps, starting with (1) establishing a baseline in protein buffer, (2) binding of liposomes through the biotinylated DSPE-PEG(2000) phospholipid, (3) establishing a new baseline, (4) binding of MinD, and finally (5) dissociation of MinD. Right: Exemplary graph resulting from sensor readouts of steps (1)–(5). (**B**) MinD binding plotted against time through association and dissociation phases (steps 4–5) at different protein concentrations, including the His-MinD-I260E mutant. (**C–E**) Same as (**B**) using the indicated respective mutants of His-MinD (G12V, K16A, and D40A).

Membrane binding appeared to be a key factor of the ATPase cycle, and since the microscopy data indicated binding of MinD monomers to the membrane, we next aimed to determine biochemically if the respective MinD variants are capable of binding to the membrane and, if so, to characterize their respective binding kinetics. To this end, we employed bio-layer interferometry (BLI), a technique capable of quantifying binding strength and protein interaction, as well as identifying reaction kinetics. Liposomes were immobilized on a biosensor to create a membrane-like surface for subsequent MinD binding assays. After successful saturation of the sensor with liposomes, MinD-liposome association and dissociation were measured; a scheme of the experimental setup is depicted in Figure 5A. As a negative control for unspecific binding, MinD-sensor interaction was tested on empty SA sensors. Furthermore, unspecific binding to liposome-saturated sensors was tested using bovine serum albumin (BSA). Both resulted in negligible, diffuse signals (data not shown).

**Figure 5—figure supplement 1.**
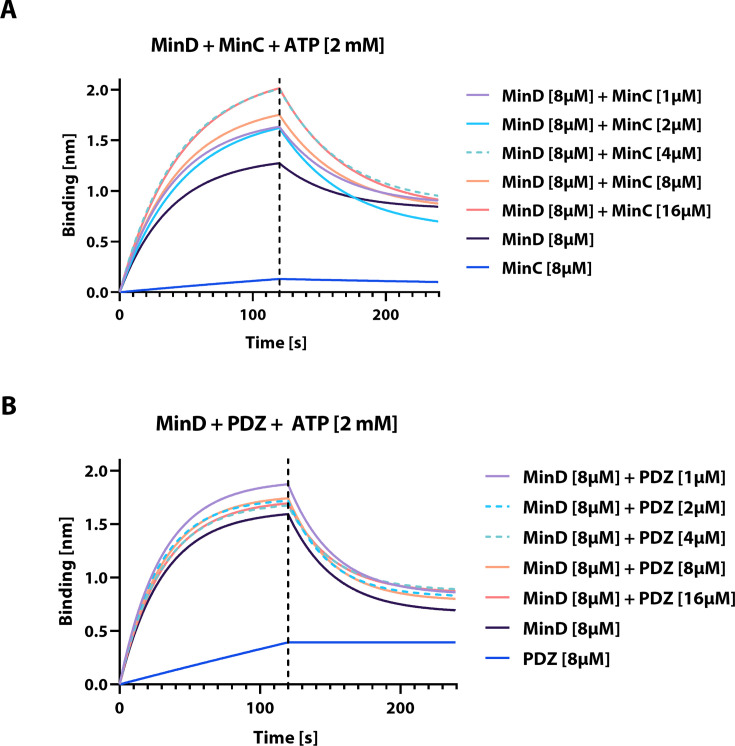
Bio-layer interferometry (BLI) analysis of interactions between His-MinD and His-MinC or His-PDZ. (**A**) MinD binding (see Figure 5A) plotted against time through association and dissociation phases (steps 4–5) at different indicated His-MinC concentrations, including His-MinD and His-MinC only controls, all in the presence of freshly added ATP [2 mM]. (**B**) Same as (**A**) using the indicated respective concentrations of His-PDZ.

**Figure 5—figure supplement 2.**
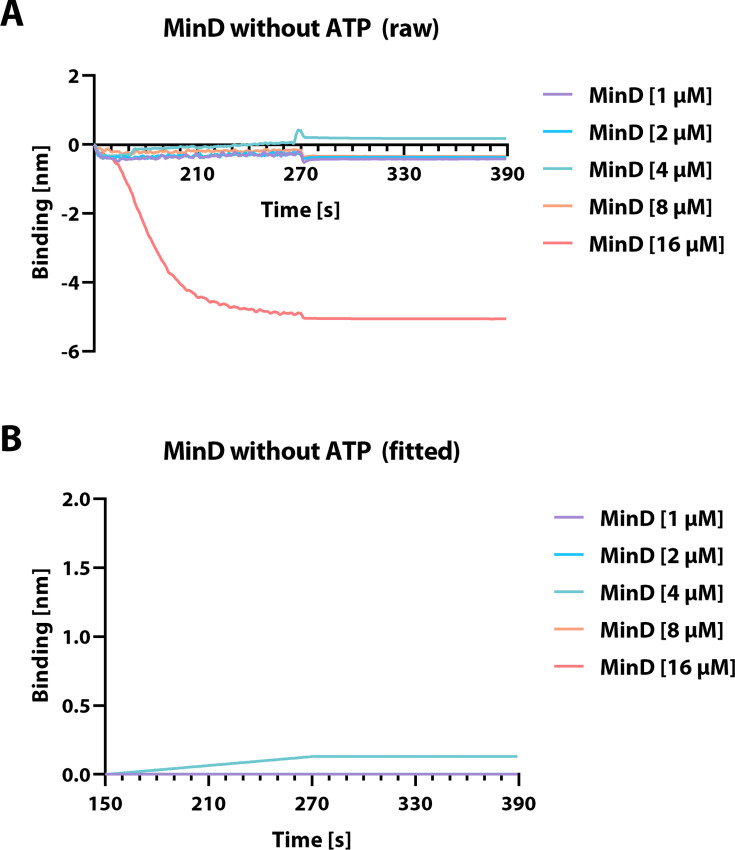
Bio-layer interferometry (BLI) analysis of His-MinD without the addition of ATP. His-MinD binding to liposome-associated BLI sensor (see Figure 5), plotted against time through association and dissociation phases at different protein concentrations without the addition of Mg^2+^-ATP.

Kinetics of His-MinD binding were tested first, using different protein concentrations between 1 µM and 16 µM in the presence of ATP. Binding to the sensor-bound liposomes scaled with increasing protein concentration, resulting in an equilibrium dissociation constant *K*_D_ = 7.17 µM and an association rate constant *k*_on_ = 3.03 × 10^3^ M^–1^ s^–1^ as a reference point for the wild-type protein (Figure 5B, Table 2). Unsurprisingly, His-MinD-I260E showed a more than 10-fold reduced affinity to the membrane with a *K*_D_ = 74.36 µM (Table 2), which is also apparent in the binding graph (Figure 5B). The marked increase in *K*_D_ is mainly due to the more than 16-fold decrease in the association rate constant *k*_on_ = 0.18 × 10^3^ M^–1^ s^–1^, while the dissociation rate constant *k*_off_ = 13.45 × 10^–3^ s^–1^ was only slightly lower than that of wild-type MinD. Next, both monomeric mutants G12V and K16A were probed, and to our surprise, were capable of membrane interaction (Figure 5C and D). This is in stark contrast to *E. coli* MinD, where dimerization needs to precede membrane binding to achieve sufficient affinity (Szeto et al., 2003; Szeto et al., 2002; Hu and Lutkenhaus, 2003; Wu et al., 2011; Lackner et al., 2003). Similar to I260E, the G12V mutant displayed an around 10-fold reduced membrane affinity equilibrium with *K*_D_ = 75.38 µM (Table 2). However, G12V only exhibited an ~5-fold reduced binding affinity with *k*_on_ = 0.63 × 10^3^ M^–1^ s^–1^ when compared to the wild-type MinD, while the *k*_off_ = 47.8 × 10^–3^ s^–1^ represents the fastest dissociation rate constant between all MinD variants and more than 2-fold increase compared to wild-type MinD (Table 2). This indicates a higher turnover and thus a quicker release of the protein from the membrane. Markedly different from this, K16A displayed a *K*_D_ of 1.36 µM, but not much lower binding rates (*k*_on_ = 2.03 × 10^3^ M^–1^ s^–1^, Figure 5C, Table 2). Instead, the low *K*_D_ can be mostly attributed to the reduced dissociation (*k*_off_ = 2.77 × 10^–3^ s^–1^), implying a much longer retention of His-MinD-K16A at the membrane (Figure 5D). Since G12V can bind ATP while K16A cannot, these findings reveal that the presence of ATP in the binding pocket affects the association and dissociation of MinD.

**Table 2.** Kinetic constants of His-MinD and variants obtained via bio-layer interferometry (BLI).

Protein	*K*_D_ [µM]	*k_on_* [M^–1^ s^–1^]	*k*_on error_	*k*_off_ [s^–1^]	*k*_off error_	*X* ^2^	*R* ^2^
**MinD**	7.17	3.03×10^3^	0.13×10^3^	21.7×10^–3^	0.33×10^–3^	16.07	0.95
**MinD-G12V**	75.38	0.63×10^3^	0.19×10^3^	47.8×10^–3^	1.13×10^–3^	13.89	0.95
**MinD-K16A**	1.36	2.03×10^3^	0.062×10^3^	2.77×10^–3^	0.088×10^–3^	6.97	0.95
**MinD-D40A**	0.74	2.39×10^3^	0.053×10^3^	1.76×10^–3^	0.069×10^–3^	6.01	0.98
**MinD-I260E**	74.36	0.18×10^3^	0.19×10^3^	13.45×10^–3^	0.38×10^–3^	3.61	0.95

*K*_D_ = equilibrium dissociation constant; *k*_on_ = association rate constant; *k*_off_ = dissociation rate constant.

Last, the His-MinD-D40A mutant protein was probed, resulting in an almost 10-fold reduced dissociation equilibrium *K*_D_ = 0.74 µM (Table 2, Figure 5E). Here, the strongly reduced membrane dissociation (*k*_off_ = 1.76 × 10^–3^ s^–1^) causes the drastic change in *K*_D_, which we expected for the trapped dimer, as it could position the two amphipathic helices into a more optimal orientation of the apolar residues toward the membrane, resulting in stronger binding and no capability of hydrolyzing ATP and thus relieving the dimer (Strahl and Hamoen, 2010; Leonard et al., 2005; Wu et al., 2011).

In summary, these BLI data demonstrate that *B. subtilis* MinD is capable of binding to the membrane in both monomeric and dimeric forms, while the presence of ATP in the binding pocket appears to affect both membrane binding and dissociation.

### MinC and the PDZ domain of MinJ have no significant impact on MinD membrane kinetics

In vivo, the integral membrane protein MinJ recruits MinD to poles and septum, which in turn recruits MinC to form a functional complex that inhibits FtsZ-ring assembly. However, the mechanistic details of how MinJ influences membrane association of MinD remain unclear. Does MinJ actively recruit MinD from the cytoplasm, or does it primarily sequester MinD already bound to the membrane? Furthermore, does MinJ promote MinD dimerization or oligomerization, potentially enhancing ATPase activity and triggering membrane dissociation? Similarly, MinC binding to MinD may influence the stability or turnover of MinD at the membrane, yet its impact on association/dissociation kinetics remains unknown. To address these questions directly, we employed BLI to test the effect of His-MinC and His-PDZ on His-MinD membrane kinetics.

After validation of the experimental setup (see Materials and methods), we next tested how His-MinC and His-PDZ affect MinD-liposome binding kinetics by subjecting liposome-coated sensors to solutions containing 8 µM His-MinD and varying concentrations [1, 2, 4, 8, and 16 µM] of His-MinC or His-PDZ, respectively (Figure 5—figure supplement 1, Table 3). MinD controls appear twice in the table because the experiments were performed using two independent purification batches. The corresponding His-MinC and His-PDZ samples were each paired with the His-MinD batch they contained. Due to batch-specific differences in His-MinD stability and degradation, we did not directly compare measurements across batches. Interestingly, neither His-MinC nor the His-PDZ domain significantly altered the membrane-binding kinetics of His-MinD in BLI experiments. The extracted *K*_D_, *k*_on_, and *k*_off_ values differed only marginally between conditions (Table 3), and these apparent shifts fell within the fitting uncertainty and typical inter-run variability of BLI. This contrasts with the slight increase in His-MinD ATPase activity observed in the presence of His-PDZ (Figure 2), which had led to the hypothesis that PDZ might promote MinD oligomerization at the membrane, enhancing ATP hydrolysis. However, the discrepancy between the two systems and the high lipid concentration (2  mg ml^–1^) in the ATPase assay vs. a liposome-coated sensor in BLI may account for the different outcomes. In the BLI experiment, limited surface area and distinct spatial organization of liposomes may restrict the ability of the soluble PDZ domain to effectively cluster MinD, unlike full-length MinJ, which is membrane-anchored via its transmembrane helices. Thus, while the PDZ domain alone appears insufficient to modulate membrane-binding kinetics of MinD, it remains plausible that the full-length MinJ could exert a more pronounced effect through spatial coordination at the membrane.

**Table 3.** Kinetic constants of His-MinD in combination with His-MinC or His-PDZ.

Protein	*K*_D_ [µM]	*k*_on_ [M^–1^ s^–1^]	*k*_on error_	*k*_off_ [s^–1^]	*k*_off error_	*X* ^2^	*R* ^2^
**MinD** *	31.7	0.27×10^3^	0.21×10^3^	8.65×10^–3^	0.26×10^–3^	4.10	0.957
**+MinC**	33.4	0.35×10^3^	0.084×10^3^	11.7×10^–3^	0.16×10^–3^	8.07	0.979
							
**MinD** *	51.1	0.34×10^3^	0.12×10^3^	17.5×10^–3^	0.24×10^–3^	2.40	0.986
**+PDZ**	47.5	0.35×10^3^	0.14×10^3^	16.6×10^–3^	0.25×10^–3^	8.98	0.973

*K*_D_ = equilibrium dissociation constant; *k*_on_ = association rate constant; *k*_off_ = dissociation rate constant.

* His-MinD control appears twice, from independent purifications. Since batches were not directly comparable, controls are only compared to samples containing the same batch. Concentrations: His-MinD 8 µM, His-MinC 1–16 µM [1, 2, 4, 8, 16], His-PDZ 1–16 µM [1, 2, 4, 8, 16], see Figure 5—figure supplement 1.

### Single-molecule localization microscopy confirms in vitro data

**Figure 6.**
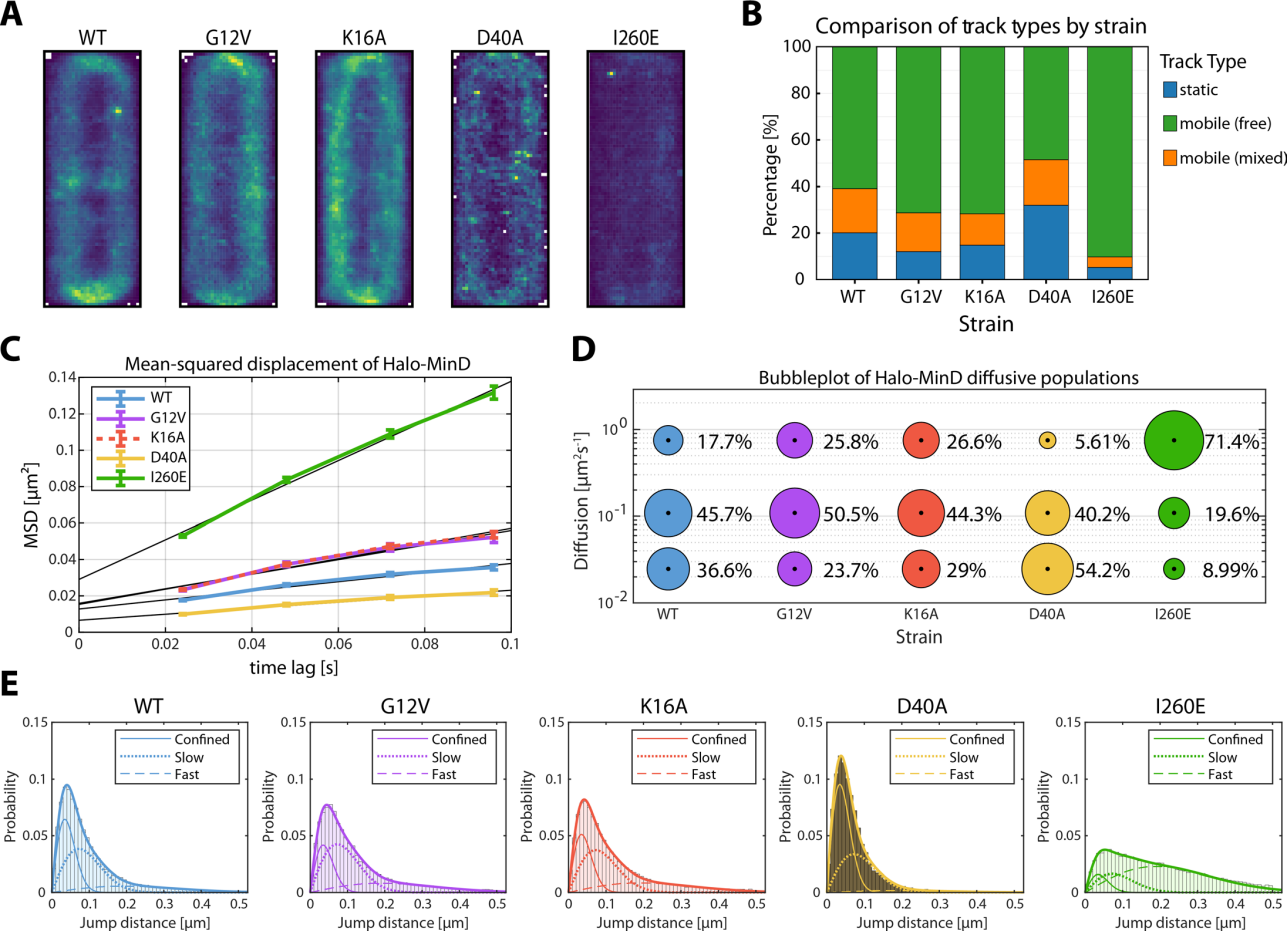
Single-molecule localization microscopy (SMLM) analysis of Halo-MinD and mutants expressed in *B*. *subtilis*. Exponentially growing *B. subtilis* cells expressing Halo-MinD and variants (*n*≥48 cells, respectively) were stained with TMR ligand and subsequently imaged. Individual protein trajectories were recorded using SMLM and analyzed with Zen Blue (Zeiss), TrackMate, the SMTracker 2.0 software package, and manual scripts in R. Minimum track-length 4 frames of 24 ms, with at least 2596 trajectories per strain. (**A**) Heatmap representation of intracellular localization of individual molecules of Halo-MinD and variants, respectively, plotted on normalized cells. Brighter colors indicate higher abundance. (**B**) Barplot of stationary localization analysis (SLA), comparing different track types within the protein population. Tracks were considered static when not leaving a circular area of 97 nm diameter within 5+ frames. Mobile populations were further divided into free and mixed tracks, where mixed tracks displayed a switch between free and confined movement. (**C**) Plot of the mean-squared displacement of Halo-MinD and variants over time, fitted with a linear fit excluding the last time lag. (**D**) Bubble plot displaying single-molecule diffusion rates of the indicated MinD fusions. Populations were determined by fitting the probability distributions of the frame-to-frame displacement (jump distance) data of all respective tracks to a three-component model (fast mobile, slow mobile, and confined protein populations). (**E**) Probability distributions of jump distances of Halo-MinD and variants. Data was fit with a three-component model, indicating confined, slow, and fast tracks.

To validate the biochemical in vitro results, we decided to utilize single-molecule localization microscopy (SMLM) with focus on single-molecule tracking (SMT), a technique allowing us to observe and evaluate MinD dynamics in vivo inside *B. subtilis* cells. We began SMLM analysis by plotting a heatmap of the intracellular localization of all individually recorded Halo-MinD molecules, as well as the mutated variants, respectively, on normalized cells (Figure 6A). This representation did not only provide a robust internal control for the imaging and post-processing process for the well-characterized localization of MinD but also revealed systemic differences in localization caused by the respective mutations. The first obvious difference is reduced MinD enrichment at the cell center and poles in the G12V or K16A variants (Figure 6A). Here, the membrane-bound MinD fraction seems to be much more evenly distributed along the membrane, similar to what we observed during wide-field microscopy (Figure 3B). This result does not only confirm the ability of MinD monomers to bind the membrane (Figure 5C and D) but also fuels the speculation that MinJ might be unable to recruit MinD in its monomeric form, so that polar and septal recruitment by MinJ requires dimeric MinD. In combination with the information obtained from BLI results (Figure 5), this could mean that the membrane itself serves as a proxy for MinD dimerization and subsequent recruitment, quickly followed by ATP hydrolysis and membrane detachment of MinD. In contrast, but in agreement with wide-field imaging (Figure 3B), the Halo-MinD-D40A mutant does appear to frequently form foci and larger local accumulations, with little protein in the cytosolic fraction (Figure 6A). This, again, could be caused by local MinJ interaction and recruitment in combination with the missing capability of membrane detachment or with increased lateral MinD interactions (Heermann et al., 2021). Finally, the I260E mutant appears evenly distributed throughout the cell (Figure 6A), as it was expected.

Independently, all protein trajectories were subjected to a stationary localization analysis (SLA) (Figure 6B). This was done by first grouping all trajectories into either a mobile or static population by testing if the molecule left a circle with a diameter of 97 nm (equaling the pixel size of the imaging setup) within at least five consecutive frames. Trajectories that were classified as mobile were further subgrouped into mixed and free trajectories, where only free trajectories never presented a confinement event (Oviedo-Bocanegra et al., 2021). In agreement with previous data, the D40A mutant displayed the highest fraction of static tracks (Figure 6B), and by far the lowest proportion of truly free trajectories (48.5%). In contrast, more than 90% of Halo-MinD-I260E molecules were classified as free with only a few trajectories classifying as static (Figure 6B), accentuating the absence of interaction with the membrane or other structures. When comparing Halo-MinD to the monomeric mutants G12V and K16A, the number of static tracks was visibly reduced in the mutants. Since both monomers are unable to dimerize, it is less likely that they can form larger clusters, which we observed in a previous study (Feddersen et al., 2021). Between both monomeric mutants, K16A displayed a slightly larger number of static trajectories (Figure 6B), which is in alignment with BLI data, where the K16A mutant dissociates much less effectively from the membrane (Figure 3C and D, Table 2).

Next, we examined the average intracellular mobility of Halo-MinD compared with the mutant variants by analyzing the mean-squared displacement (MSD, Figure 6C). In MSD analysis, the traveled distances of recorded trajectories are plotted against a given time lag (Δ*t*) as a function of Δ*t*, here one imaging frame (24 ms), where the slope can indicate the speed (Barak and Webb, 1982; Goulian and Simon, 2000; Weimann et al., 2013). As expected, the I260E mutant displayed the fastest average mobility, since it does not bind the membrane effectively anymore (Figure 6C). In contrast, the D40A mutant was the slowest recorded protein, indicated by the only mild incline (Figure 6C). Since we expect most of the proteins’ population to be membrane bound, and furthermore observed larger foci that suggest cluster formation, this was expected. Halo-MinD wild-type protein appeared to be more mobile in comparison (Figure 6C), as it should exist in both monomeric and dimeric conformations and either freely diffusive in the cytosol or interact with the membrane. Last, the two monomeric mutants G12V and K16A displayed extremely similar average speeds that were both faster than the wild-type protein (Figure 6C). Since these MinD variants cannot form dimers, general diffusion should be faster, as there should be no MinD dimers or oligomers, and possibly no recruitment by MinJ.

If the underlying movement in MSD analysis can be described by simple Brownian motion, the gradient of the curve is proportional to the diffusion coefficient *D* (Weimann et al., 2013). We know, however, that MinD interacts at least with MinC, MinJ, the membrane, and itself, in combination with the fact that bacterial cells exhibit natural confinement. With this information, one cannot assume simple Brownian motion; therefore, we have chosen jump distance (JD) analysis as a more accurate assessment to describe Halo-MinD dynamics (Figure 6D and E). In JD analysis, the distances molecules travel between subsequent frames are plotted in a probability distribution (Figure 6E), and diffusion coefficients are obtained by curve fitting (Weimann et al., 2013), in this case with a three-component model separating fast, slow, and confined molecules (Figure 6D and E). To accurately compare population sizes, the simultaneous option was chosen in the SMTracker software, fixing the diffusion coefficients for every MinD variant (Oviedo-Bocanegra et al., 2021). To also obtain individual, variant-specific diffusion coefficients, the same data was additionally fitted individually (non-simultaneous square displacement [SQD] analysis in SMTracker2, Table 4).

**Table 4.** Diffusion coefficients obtained from non-comparative square displacement (SQD) analysis of Halo-MinD and variants fitted with three populations.

Strain	Fast diffusive [µm^2^ s^–1^]	Slow diffusive [µm^2^ s^–1^]	Confined [µm^2^ s^–1^]
**MinD WT**	0.60 (22.3%)	0.084 (50.4%)	0.020 (27.3%)
**MinD-G12V**	0.68 (28.5%)	0.10 (47.5%)	0.026 (24.0%)
**MinD-K16A**	0.70 (27.3%)	0.11 (43.9%)	0.024 (28.8%)
**MinD-D40A**	0.44 (13.7%)	0.063 (50.0%)	0.021 (36.3%)
**MinD-I260E**	0.76 (73.4%)	0.095 (20.3%)	0.022 (6.3%)

Percentages indicate relative population size.

Generally, the results of JD analysis correlated well with our in vitro data. When dissecting the different populations in the wild type, more than a third of all proteins appear to be confined (36.6%, Figure 6D), which likely comprises membrane-bound protein that is further stabilized by protein-protein interactions via lateral interaction of MinD with itself, and possibly MinC and MinJ. The slow mobile population was the largest (45.7%, Figure 6D), and likely summarizes different conformations of MinD that interact with the membrane, but are still diffusive and not captured or stabilized through further interactions. The third, fast diffusive population (17.7%, Figure 6D) likely reflects cytosolic MinD. These results stress the importance of the MinD ATPase cycle for its localization and dynamics, as these are severely altered in the MinD mutants.

In detail, the probability distributions of Halo-MinD-D40A and I260E (Figure 6E) illustrate the strong shift between MinD molecules in a confined (D40A) and a diffusive state (I260E) best. When examining the different populations, most Halo-MinD-D40A molecules were classified as confined (54.2%) and slow mobile (40.2%), whereas I260E only displayed ~9% of confined molecules (Figure 6D), matching prior results and expectations of the membrane-binding mutant. When comparing D40A to wild-type Halo-MinD, the largest shift is observed between the fast mobile and confined populations, shifting toward a distinctively more confined regimen in the D40A mutant (36.6% WT, 52.2% D40A; Figure 6D) and visible disappearance of the fast population in the probability distribution (Figure 6E). These results are in line with the previous observations, as a trapped dimer of MinD will remain at the membrane at much higher efficiency (Figure 5E and Table 2) and appeared to form foci and clusters (Figures 3A and 6B). G12V and K16A mutants, on the other hand, showed an increase in fast mobile populations (WT 17.7%, G12V 25.8%, K16A 26.6%; Figure 6D, E), which could be attributed to a reduced interaction with MinD interaction partners (MinC, MinD, MinJ, and plasma membrane). This notion is supported by the decrease in confined molecules for both G12V and K16A mutants compared to the wild type (WT 36.6%, G12V 23.7%, K16A 29%; Figure 6D and E), which are expected to be membrane bound and likely stabilized through other protein-protein interactions, which may be reduced or absent in the monomeric conformation. This notion is also supported by the absence of a MinD gradient in epifluorescence images of G12V and K16A mutants (Figure 3B), a pattern usually formed through sequestering via MinJ. Finally, when comparing both monomeric variants, the K16A mutant displays a noticeable shift between the slow diffusive (G12V 50.5%, K16A 44.3%; Figure 6D) and confined populations (23.7% G12V, 29% K16A; Figure 6D). The larger confined population of Halo-MinD-K16A is consistent with the BLI results (Figure 5D, Table 2), where K16A appears to have a lower dissociation from the membrane, increasing its likelihood of confinement, possibly caused by the inability to bind ATP.

In summary, these results emphasize the importance of the MinD ATPase cycle for the functionality of the Min system in *B. subtilis*. Even though *B. subtilis* MinD is not capable of oscillation, its intracellular dynamics are highly dependent on the functionality of the MinD ATPase domain and its catalytic site, which is reflected by the variety of localization and interaction defects displayed by the MinD mutants on a molecular level, that all result in severe division anomalies.

### MinC and MinJ have different effects on MinD protein dynamics in SMLM

**Figure 7.**
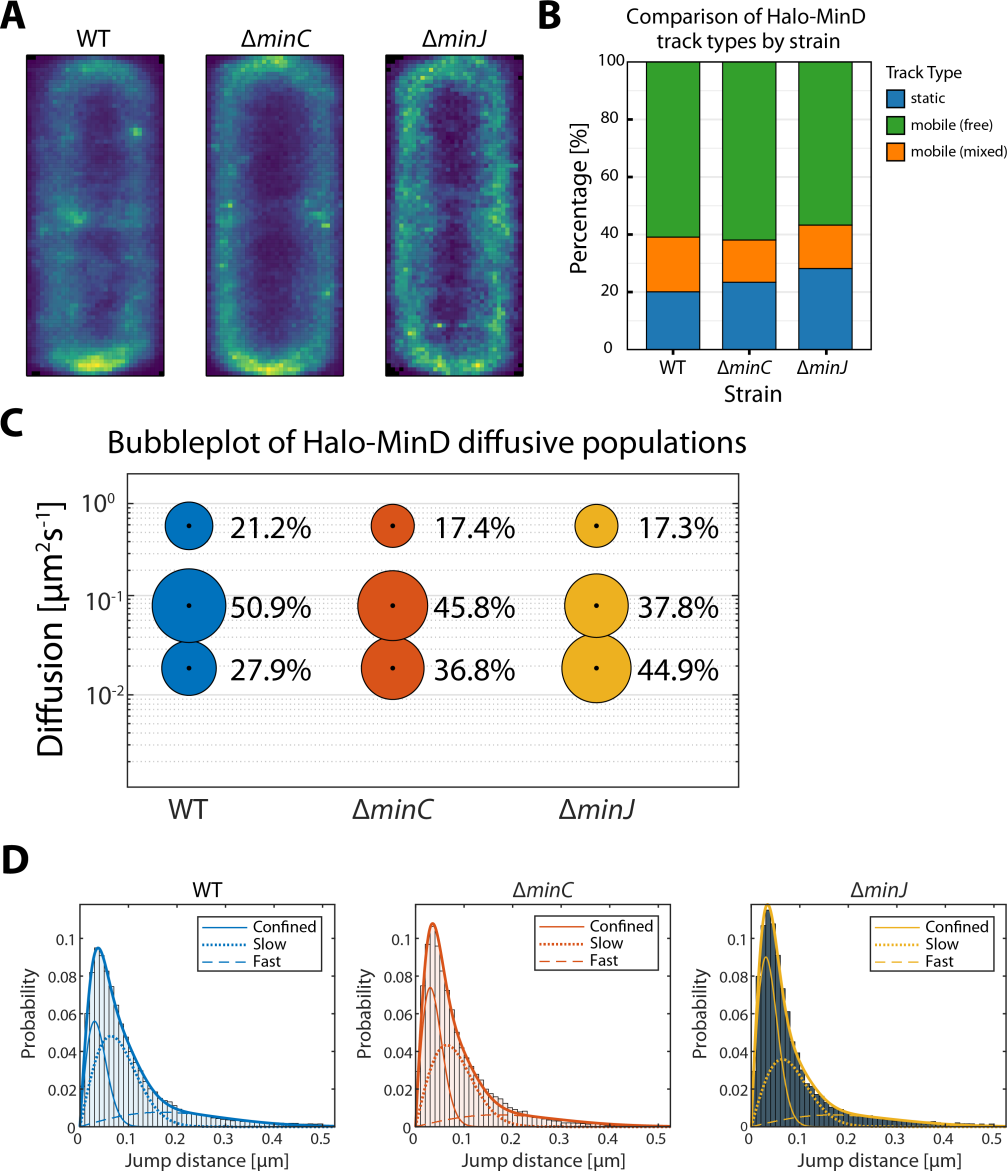
Single-molecule localization microscopy (SMLM) analysis of Halo-MinD expressed in *B*. *subtilis* wild-type and mutant backgrounds. Exponentially growing *B. subtilis* cells expressing Halo-MinD in the indicated genetic backgrounds (*n*≥115 cells, respectively) were stained with TMR ligand and subsequently imaged. Individual protein trajectories were recorded using SMLM and analyzed with Zen Blue (Zeiss), TrackMate, the SMTracker 2.0 software package, and manual scripts in R. Minimum track-length 4 frames of 24 ms, with at least 2688 trajectories per strain. (**A**) Heatmap representation of intracellular localization of individual molecules of Halo-MinD in the indicated genetic background, respectively, plotted on normalized cells. Brighter colors indicate higher abundance. (**B**) Barplot of stationary localization analysis (SLA), comparing different track types within the protein population. Tracks were considered static when not leaving a circular area of 97 nm diameter within 5+ frames. Mobile populations were further divided into free and mixed tracks, where mixed tracks displayed a switch between free and confined movement. (**C**) Bubble plot displaying single-molecule diffusion rates of Halo-MinD in different genetic backgrounds. Populations were determined by fitting the probability distributions of the frame-to-frame displacement (jump distance) data of all respective tracks to a three-component model (fast mobile, slow mobile, and confined protein populations). (**D**) Probability distributions of jump distances of Halo-MinD in different genetic backgrounds. Data was fit with a three-component model, indicating confined, slow, and fast tracks.

MinC and MinJ interact with MinD in vivo, suggesting protein dynamics should be affected by this. However, in our ATPase and BLI experiments, MinC had only minor effects on ATPase activity and membrane-binding kinetics of MinD, while the PDZ domain slightly enhanced ATPase activity without altering membrane-binding kinetics of MinD. To validate these observations in vivo and to identify differences between MinD interacting with a cytosolic PDZ domain or a full-length MinJ, we performed the same SMLM workflow and analysis pipeline described in the previous section on cells expressing Halo-MinD in Δ*minC* and Δ*minJ* backgrounds and compared them to the wild-type background (Figure 7, Table 5).

**Table 5.** Diffusion coefficients obtained from non-comparative square displacement (SQD) analysis of Halo-MinD in different genetic backgrounds fitted with three populations.

Strain	Fast diffusive [µm^2^ s^–1^]	Slow diffusive [µm^2^ s^–1^]	Confined [µm^2^ s^–1^]
**WT**	0.57 (22.1%)	0.083 (50.2%)	0.019 (27.7%)
**Δ*minC***	0.31 (29.3%)	0.059 (44.3%)	0.01 (26.4%)
**Δ*minJ***	0.6 (19%)	0.069 (41.2%)	0.018 (39.7%)

Percentages indicate relative population size.

When comparing the average Halo-MinD distribution between the respective strains, the Δ*minC* mutant exhibited a similar intracellular localization pattern as the wild type, with strong membrane enrichment and highest concentrations at midcell and poles (Figure 7A). However, Δ*minC* cells interestingly exhibited a slightly reduced cytosolic signal and a corresponding increased membrane association. As expected, Δ*minJ* cells lacked the characteristic septal accumulation. Instead, Halo-MinD was distributed relatively uniformly along the membrane, consistent with MinJ being required for efficient sequestering of MinD to the future division site and cell poles.

In the SLA (Figure 7B), Halo-MinD in the Δ*minC* strain displayed only a modest increase in the static fraction compared to wild type (23.4% from 20.1%). This shift can be attributed to a reduction in the mixed, likely membrane-interacting population (from 19.0% to 14.7%), whereas the freely mobile fraction remained essentially unchanged (61% to 62%). These results indicate that in the absence of MinC, less MinD moves along the membrane and a slightly larger proportion becomes immobilized. Although the effect size is small, it suggests that MinD may form larger or slightly more stable membrane-associated assemblies when MinC is absent in vivo. In the Δ*minJ* background, the static Halo-MinD population surprisingly increased (20.1% to 28.2%), together with respective reductions in both the freely diffusing cytosolic pool (61.0% to 56.6%) and the mixed, likely membrane-interacting population (19.0% to 15.1%). This redistribution suggests that MinJ contributes to efficient MinD turnover on the membrane, in line with MinD ATPase data with His-PDZ (Figure 7). In the absence of MinJ, MinD appears to remain membrane-bound for longer, consistent with impaired detachment or reduced cycling between membrane-associated states. One possible explanation is that MinD may form more stable or aggregation-prone assemblies when MinJ is absent, whereas the presence of MinJ could promote more dynamic membrane association and hence sharper local accumulations and cycling of MinD, necessary for enriched polar and septal localization. Jump-distance analysis with fixed diffusion coefficients corroborated these findings (Figure 7C and D), showing that Δ*minJ* cells contained a substantially enlarged confined population of MinD (27.9% vs. 44.9%), together with a large reduction of the slow diffusive population (50.9% vs. 37.8%). Fitting the data without fixed diffusion coefficients produced the same trend (Table 5), indicating that the shift reflects MinD spending more time in the respective state rather than actual changes in the underlying diffusive coefficients.

In the Δ*minC* strain, Halo-MinD shows a slight redistribution toward the confined state, with an increased fraction of very slow or immobile molecules (27.9% vs. 36.8%) and a corresponding decrease in both the fast and intermediate populations (50.9% vs. 45.8% and 21.2% vs. 17.4%, respectively, Figure 7C and D). SQD analysis without fixed diffusion coefficient (Table 5) shows that the diffusion coefficients of the fast and intermediate populations are reduced compared with wild type, indicating that the mobile molecules themselves move more slowly on average. Combined, this could suggest that in the absence of MinC, membrane-bound MinD is more prone to forming clusters or larger aggregates, which restrict mobility and reduce the efficiency of its membrane cycling.

Together, these data suggest that MinJ promotes productive membrane cycling of MinD, and in its absence, MinD accumulates more often in a confined, likely non-productive membrane-associated state, whereas MinC plays a comparatively minor regulatory role, where it passively contributes to MinD mobility by preventing excessive clustering and facilitating proper membrane-associated dynamics.

## Discussion

In bacteria, the oscillation of MinCDE in *E. coli* is one of the best-studied examples of a reaction-diffusion system, and the two core components MinD and MinE cycle between membrane and cytosol in an ATP-dependent manner, generating a standing wave with a time-resolved minimum at midcell (Rowlett and Margolin, 2013; Bramkamp and van Baarle, 2009). Here, binding and unbinding of the components to the membrane surface influences the diffusion coefficients and controls the dynamic activity of MinC (Ramm et al., 2019). While the *E. coli* MinCDE system is one of the best-studied examples of intracellular pattern formation in bacteria (Bramkamp and van Baarle, 2009; Ramm et al., 2019; Kretschmer and Schwille, 2016; Halatek et al., 2018), the Min system in *B. subtilis* remains less well characterized. In a previous study (Feddersen et al., 2021), we suggested that gradient formation depends on ATPase-driven MinD dynamics, backed by PALM and FRAP analysis as a basis for a mathematical model. However, due to a lack of biochemical data, we had to assume that the *B. subtilis* MinD cycles between membrane-bound and cytosolic localizations based on ATP binding and hydrolysis, similar to *E. coli* MinD (Feddersen et al., 2021). A further gap in our knowledge of the *B. subtilis* Min system concerned the activation mechanism of MinD in the absence of a MinE homolog.

To address this gap, we first analyzed *B. subtilis* MinD biochemically in vitro. Purified MinD showed basal ATPase activity that drastically increased upon liposome addition. Although *E. coli* MinD similarly requires lipids, it additionally depends on MinE for full activation (Hu and Lutkenhaus, 2001). The reported maximum ATPase rate, around 20 nmol mg⁻¹ min⁻¹ (Hu and Lutkenhaus, 2001; Hu and Lutkenhaus, 2003), was nearly identical to that of *B. subtilis* MinD, indicating that membrane binding alone is sufficient to trigger robust ATP hydrolysis in the *Bacillus* system. When comparing to other MinD/ParA ATPases that have been biochemically characterized, the activity level of *B. subtilis* MinD is on the lower end of the spectrum with a *k*_cat_ of 36.27 hr^−1^. Soj, the ParA homolog in *B. subtilis*, reaches more than 2-fold higher catalytic activity (83.6 hr^–1^) when fully activated by supplementing Spo0J (ParB) and DNA (Scholefield et al., 2011), similarly to Soj in *Thermus thermophilus* combined with Spo0J and *parS* containing DNA (Leonard et al., 2005). Studies investigating the activity levels of ParA in *Caulobacter crescentus*, purified in the presence of ATP, determined a *k*_cat_ of 0.79 min^–1^ (47.4 hr^–1^) in the absence of ParB, increasing almost 5-fold to 3.7 min^–1^ (222 hr^–1^) in the presence of ParB (Easter and Gober, 2002). A more recent study found the *C. crescentus* ParA *k*_cat_ increase from 5.8 hr^−1^ to 120 hr^–1^ upon addition of ParB (Lim et al., 2014).

These findings suggest that membrane association is the primary trigger for MinD ATPase activity in *B. subtilis*. Specifically, the comparison of maximum velocities, activity stimulation factors, and *K*_M_ values between the *E. coli* and *B. subtilis* MinD proteins suggests that a functional homolog of the MinE protein is not necessary in *B. subtilis*. At the same time, our biochemical data indicate that MinJ/PDZ increases MinD ATP hydrolysis rates at least to a modest extent (between 22% and 37%, Figure 2B), pointing to a broader regulatory influence of MinJ on MinD that becomes evident from additional mechanistic analyses discussed below. In contrast, the presence of MinC led only to a slight decrease in MinD ATPase activity in our ATPase assays (~5%, Figure 2A). This modest inhibitory effect suggests that MinC does neither directly stimulate MinD turnover nor inhibit it, and may instead stabilize MinD in a conformation or assembly state with mildly reduced catalytic activity, as discussed below.

With the goal of testing the binding of isolated MinD to the membrane, we designed a BLI experiment in which we immobilized liposomes and measured the binding of purified MinD proteins. Importantly, without addition of ATP (and ADP present from purification), we observed only very weak MinD binding to the liposomes (Figure 5—figure supplement 2), indicating that MinD membrane binding and dissociation are coupled to the ATP hydrolysis cycle or the nucleotide binding state. In the presence of ATP, MinD readily binds to the liposomes with high affinity. The ParA/MinD family is well studied, and mutations producing strictly monomeric (G12V, K16A), dimeric (D40A), or membrane-binding-deficient (I260E) MinD variants are well established (Strahl and Hamoen, 2010; Karoui and Errington, 2001; Leonard et al., 2005). The most intriguing observation of the binding experiments is that MinD monomers can apparently bind to the liposome membrane in vitro. Both monomeric mutants (G12V and K16A) are readily binding to the membrane, but the release of the proteins from the membrane surface differs drastically. While the K16A mutant, unable to bind ATP, has a slow membrane release rate (*k*_off_ rate of only 2.77*10^–3^ s^–1^), the G12V monomeric mutant, which can still bind ATP, is released fast from the membrane (*k*_off_ rate 47.8*10^–3^ s^–1^). The D40A mutant of MinD, which is thought to be a stable ATP-bound dimer, releases with very slow kinetics (*k*_off_ rate 1.76*10^–3^ s^–1^). This indicates that ATP hydrolysis accelerates MinD release from the membrane and that ATP binding itself influences MinD association and dissociation kinetics, although we cannot entirely exclude that the mutations introduce subtle structural changes that affect membrane affinity. These findings crucially support a reaction-diffusion model in which the MinD ATPase cycle is essential for correct pattern formation of *B. subtilis* MinD. When testing the impact of MinC on MinD membrane-binding kinetics, no significant differences were observed, suggesting that MinC does not substantially influence the binding dynamics of MinD, in-line with the collective view of MinC as a passenger rather than a driver of MinD dynamics (Ramm et al., 2019). It would have been desirable to study the interaction of MinD with reconstituted full-length MinJ proteins to specifically investigate the influence that MinJ has on the membrane interaction of MinD. Unfortunately, we did not succeed in the purification of a full-length MinJ protein so far. MinJ contains seven predicted transmembrane helices and displays a pronounced instability, rendering purification difficult. While the binding kinetics were unaffected during BLI, our in vitro ATPase experiments combining the soluble PDZ domain of MinJ with MinD already demonstrate that MinJ possibly modulates MinD:membrane interactions to some extent through an effect on the hydrolysis efficiency.

To clarify this point and to better understand the isolated MinD membrane interaction under in vivo conditions, we turned to state-of-the-art microscopy (SMLM) (Turkowyd et al., 2016; Yao and Carballido-López, 2014).

SMLM data comparing the behavior of MinD in wild-type vs. ∆*minJ* background (Figure 7) collectively suggest that MinJ stabilizes correct pattern formation of MinD. By promoting efficient membrane detachment and maintaining MinD in a dynamically cycling state, MinJ appears to ensure that MinD remains properly redistributed throughout the cell cycle rather than becoming sequestered in nonproductive clusters. This organizational role likely underlies the ability of MinJ to position MinD, and thus MinC, at the appropriate cellular sites (Patrick and Kearns, 2008; Bramkamp et al., 2008), thereby supporting robust spatial regulation of division. However, a short-lived MinJ-mediated stabilization of MinD dimers at the membrane would be comparable to the stabilizing effect that MinE has on MinD oligomers in *E. coli* (Loose et al., 2011; Vecchiarelli et al., 2016), and was previously observed in our in vivo FRAP studies (Feddersen et al., 2021). In contrast to MinJ, MinC did not seem to have a major impact on MinD dynamics in vivo. When observing MinD in the absence of *minC*, the main effect was a subtle reduction in MinD cycling activity, shifting a small fraction of MinD into more static or confined membrane-associated states. In the wild-type situation, MinC may passively restrict uncontrolled aggregation by capping or destabilizing certain interfaces, or conversely transiently stabilize short-lived assemblies required for regulation.

The locked MinD dimers (D40A mutant) frequently nucleated into larger membrane clusters. This cluster formation is underlined by the drastic increase in the confined/immobile population (54.2%) of D40A in SMLM. This increase in the static population comes at the expense of freely diffusive protein (~5.6%), indicating that the vast majority of MinD-D40A is membrane-associated. We observed a clear enrichment of these MinD clusters at the cell poles and septa, lending support to the notion that MinD dimers are specifically stabilized/recruited by MinJ. This interpretation is supported by previous data showing that in the absence of MinJ, MinD proteins are randomly localized along the membrane in clusters, but not enriched at poles or (in a *minJ* mutant background rare) septa (van Baarle and Bramkamp, 2010). In line with this idea, the monomeric MinD variants (G12V, K16A) are evenly bound to the membrane surface along the entire cell length.

The fact that most monomeric MinD appears membrane-associated in vivo also supports our in vitro measurements in which monomeric MinD variants efficiently bind the membrane, a notion strongly supported by a recent report that examined the localization of the same monomeric variants in epifluorescence microscopy (Bohorquez et al., 2024). The time-resolved MinD gradient is lost in all mutants – including D40A – which should still be recruited by MinJ, suggesting the ATPase cycle, and thus MinD cycling is indeed necessary for proper formation of a gradient, as suggested before (Feddersen et al., 2021). Our data are in perfect agreement with recent in vitro data collected with the *E. coli* MinD using mass-sensitive particle tracking (MSPT) (Heermann et al., 2021). MSPT analysis revealed that *E. coli* MinD forms lateral interactions, leading to higher-order oligomers (trimer, tetramer, and even higher oligomers). It was speculated that dimerization of *E. coli* MinD at the membrane leads to conformational changes, opening an additional interaction surface of MinD for lateral interaction (Heermann et al., 2021). These lateral MinD interactions were thought to recruit further monomeric MinD from solution to the membrane, thereby forming a nucleation for larger clusters. The MinD cluster formation that we observed in *B. subtilis* using SMLM (Feddersen et al., 2021) is likely in vivo evidence for this cooperative membrane binding. It should be noted that the MSPT analysis was not sensitive enough to detect monomer binding to the membrane, but this was explicitly not ruled out (Heermann et al., 2021). Addition of a second amphipathic helix to the *E. coli* MinD, however, was shown to promote monomer binding of *E. coli* MinD to the membrane (Heermann et al., 2020). Thus, the difference between the *E. coli* and *B. subtilis* MinD is likely the higher membrane-binding capacity of the *Bacillus* protein. The observed cooperativity in membrane binding and thus nucleation, as shown for the *E. coli* MinD in vitro (Heermann et al., 2020; Heermann et al., 2021) and here for the *B. subtilis* MinD in vitro and in vivo, is required for pattern formation by conferring nonlinearity (Feddersen et al., 2021; Miyagi et al., 2018; Halatek and Frey, 2012).

Consistent with this model, our SMT data revealed three major dynamic states of MinD: fast cytosolic diffusion, a slower membrane-associated pool, and a confined fraction corresponding to clustered or multimeric assemblies. Most of the membrane-binding-deficient mutant I260A shifted drastically into the fast, cytosolic population, validating this assignment. Conversely, the locked-dimer (D40A) mutant showed a pronounced increase in confined, slow-moving molecules, in line with its strong tendency to nucleate clusters. Monomeric variants (G12V, K16A) displayed reduced confinement, supporting the view that dimerization is required for higher-order assembly or recruitment. Together, these observations indicate that MinD dynamically cycles between cytosol and membrane in an ATP-dependent manner. Thus, we conclude that membrane-bound dimers of MinD have a much stronger tendency to interact with and recruit binding partners (including self-nucleation). This finding is further supported by a recent report that analyzed MinD dynamics in vivo using diffraction-limited microscopy (Bohorquez et al., 2024). In this study, a MinD-D40A mutant hyper-recruited MinC, leading to cluster formation of MinCD at the membrane. Deletion of MinC restored the MinD gradient formation, indicating that MinC might indeed be part of a MinC:MinD co-polymer, as suggested for the *E. coli* system (Ghosal et al., 2014). This assumption is in good agreement with our in vivo findings, where self-nucleation seems to increase drastically for the D40A mutant or in the absence of MinJ. In contrast, monomeric G12V and K16A mutants do not display this behavior, in line with monomeric MinD variants not being able to interact with MinC, which was similarly observed through wide-field microscopy in the same study (Bohorquez et al., 2024).

Collectively, our data reveal that the MinD ATPase cycle is essential for regulated membrane binding and release, which is crucial for the formation of a sharp MinD gradient at the septum (Feddersen et al., 2021). However, in contrast to *E. coli* MinD, monomeric *B. subtilis* MinD can bind the membrane with higher affinity, suggesting that MinD also dimerizes at the membrane surface. Dimer formation is essential for ATPase activity, as indicated by our in vitro measurements, and is necessary for efficient membrane detachment. The main characteristics of the *E. coli* MinD with respect to membrane binding, self-interaction leading to nucleation, and membrane detachment are therefore shared in the *B. subtilis* protein. The difference, that ATP hydrolysis of the *B. subtilis* MinD does not require a trigger other than membrane binding, makes sense in a system that does not need to oscillate, but rather accumulate and sustain a sharp gradient at the current site of division. However, the balance of a dynamic membrane cycle of MinD seems to be fine-tuned by the interaction with MinC and especially MinJ, which appears to keep MinD in a productive state and protects it from aberrant aggregation, while recruiting it toward poles and septum, the intended site of action of MinC.

While these findings elucidate the functional principles of the *B. subtilis* Min system further, it is not clear if and how broadly they can be inferred on other rod-shaped, Gram-positive bacteria that possess a Min system. While Min systems are often summarized into either oscillating MinCDE systems or DivIVA-dependent gradients, the group of spore-forming Firmicutes appear to have rather individual sets of Min proteins. Often, they contain homologs from both systems, presumably necessary to individually regulate and allow the switch between symmetric (vegetative) and asymmetric (sporulation) division (Jamroškovič et al., 2012). A recent study demonstrated, for example, that *Clostridioides difficile* MinDE proteins expressed in *B. subtilis* can display oscillatory behavior, interfering with sporulation (Makroczyová et al., 2016), while DivIVA in *C. difficile* appears to directly interact with MinD without a MinJ-like bridge protein (Valenčíková et al., 2018). Furthermore, a very recent study identified another protein (MrpA) in *C. difficile* that directly interacts with MinD, an interaction leading to aberrant cell division, as well as affecting colony morphology (Mehra et al., 2025). The extent to which these mechanisms operate simultaneously or are differentially utilized under specific physiological conditions, such as vegetative growth or sporulation, remains an open question. Our findings on the Min system in *B. subtilis* therefore likely represent only one end of a broad spectrum of bacterial division site selection mechanisms.

## Materials and methods

**Key resources table:** 

Reagent type (species) or resource	Designation	Source or reference	Identifiers	Additional information
Antibody	Penta-His antibody (Mouse monoclonal)	QIAGEN	Cat#: 34660, RRID:AB_2619735	WB (1:1000)
Antibody	Goat anti-Mouse IgG (H+L) Highly Cross-Adsorbed Secondary Antibody, AP	Thermo Fisher Scientific	Cat#: A16081, RRID:AB_2534755	WB (1:10,000)
Strain, strain background (*B. subtilis*)	168	Laboratory collection	Strain_ID:168	Wild type; trpC2
Strain, strain background (*B. subtilis*)	minC::lox71-kanR-lox66	Addgene	Strain_ID:BKK28000	*B. subtilis* BKK Library
Strain, strain background (*B. subtilis*)	minJ::tet	Bramkamp et al., 2008	Strain_ID:RD021	
Strain, strain background (*B. subtilis*)	minD::lox71-kanR-lox66	Addgene	Strain_ID:BKK27990	*B. subtilis* BKK Library
Strain, strain background (*B. subtilis*)	minD::lox71-kanR-lox66	This paper	Strain_ID:BHF077	BKK27990 gDNA ->168
Strain, strain background (*B. subtilis*)	minD::aad9-Halo-minD	This paper	Strain_ID:BHF078	EHF054 ->BHF077
Strain, strain background (*B. subtilis*)	minD::aad9-Halo-minD-G12V	This paper	Strain_ID:BHF079	EHF055 ->BHF077
Strain, strain background (*B. subtilis*)	minD::aad9-Halo-minD-K16A	This paper	Strain_ID:BHF080	EHF056 ->BHF077
Strain, strain background (*B. subtilis*)	minD::aad9-Halo-minD-D40A	This paper	Strain_ID:BHF081	EHF057 ->BHF077
Strain, strain background (*B. subtilis*)	minD::aad9-Halo-minD-I260E	This paper	Strain_ID:BHF082	EHF058 ->BHF077
Strain, strain background (*B. subtilis*)	minD::aad9-Halo-minD, minC::lox71-kanR-lox66	This paper	Strain_ID:BHF083	BKK28000 gDNA ->BHF078
Strain, strain background (*B. subtilis*)	minD::aad9-Halo-minD, minJ::tet	This paper	Strain_ID:BHF084	RD021 gDNA ->BHF078
Strain, strain background (*E. coli*)	NEB 5-alpha	New England Biolabs (C2987H)	Strain_ID:NEB 5-alpha	*fhuA2Δ(argF-lacZ)U169 phoA glnV44 Φ80Δ(lacZ)M15 gyrA96 recA1 relA1 endA1 thi-1 hsdR17*
Strain, strain background (*E. coli*)	BL21(DE3)pLysS	Promega	Cat#: L1195	F–, *ompT, hsdS*_B_ (r_B_–, m_B_–), *dcm*, *gal*, λ(DE3), pLysS, Cm^r^
Recombinant DNA reagent	pUC18 (plasmid)	Norrander et al., 1983	Plasmid_ID:pUC18	*lacZα*, *pMB1 ori*, *bla* (Ap^R^)
Recombinant DNA reagent	pET-28a (+) (plasmid)	Novagen	Plasmid_ID:pET-28a (+)	*kan^R^*, *T7lac*, *f1 ori*, N-term His-tag/thrombin/T7-Tag
Recombinant DNA reagent	pET-28a-minC	This paper	Plasmid_ID:NAP012	MinC amplified from 168
Recombinant DNA reagent	pET-28a-minD	This paper	Plasmid_ID:NAP013	MinD amplified from 168
Recombinant DNA reagent	pET-28a-minD-G12V	This paper	Plasmid_ID:EHF045	Mutagenesis of NAP013
Recombinant DNA reagent	pET-28a-minD-K16A	This paper	Plasmid_ID:EHF046	Mutagenesis of NAP013
Recombinant DNA reagent	pET-28a-minD-I260E	This paper	Plasmid_ID:EHF047	Mutagenesis of NAP013
Recombinant DNA reagent	pET-28a-minD-D40A	This paper	Plasmid_ID:EHF052	Mutagenesis of NAP013
Recombinant DNA reagent	pET-28a-PDZ	This paper	Plasmid_ID:CD001	Truncated MinJ PDZ domain
Recombinant DNA reagent	pUC18mut-minDNUp-aad9-Halo-minD	Laboratory stock	Plasmid_ID:EHF042	
Recombinant DNA reagent	pUC18-minC-aad9-haloTag-minD-minDdown	This paper	Plasmid_ID:EHF054	NEBuilder HiFi Assembly
Recombinant DNA reagent	pUC18-minC-aad9-haloTag-minD-G12V-minDdown	This paper	Plasmid_ID:EHF055	Mutagenesis of EHF054
Recombinant DNA reagent	pUC18-minC-aad9-haloTag-minD-K16A-minDdown	This paper	Plasmid_ID:EHF056	Mutagenesis of EHF054
Recombinant DNA reagent	pUC18-minC-aad9-haloTag-minD-D40A-minDdown	This paper	Plasmid_ID:EHF057	Mutagenesis of EHF054
Recombinant DNA reagent	pUC18-minC-aad9-haloTag-minD-I260E-minDdown	This paper	Plasmid_ID:EHF058	Mutagenesis of EHF054
Sequence-based reagent	MinC-NdeI-F	This paper	Oligo_ID:NA016	GTCATCATATGGTGAAGACCAAAAAGCAG
Sequence-based reagent	MinC-XhoI-R	This paper	Oligo_ID:NA017	GTCATCTCGAGTCACATTCCTCCCTCAAG
Sequence-based reagent	MinD-NdeI-F	This paper	Oligo_ID:NA018	GTCATCATATGTTGGGTGAGGCTATCGTA
Sequence-based reagent	MinD-XhoI-R	This paper	Oligo_ID:NA019	GTCATCTCGAGTTAAGATCTTACTCCGAA
Sequence-based reagent	GC-1-pUC18toMinC-F	This paper	Oligo_ID:HF0245	GCCTGCAGGTCGACTCTAGAGGATCCCCGGAACAAAGAATGGACTAACATTG
Sequence-based reagent	GC-1-MinCtoSpec-R	This paper	Oligo_ID:HF0246	ACATCCTTTCACCCTTCACATTCCTCCCTCAAG
Sequence-based reagent	GC-2-MinCtoSpec-Halo-F	This paper	Oligo_ID:HF0247	GGGAGGAATGTGAAGGGTGAAAGGATGTACTTAAAC
Sequence-based reagent	GC-2-Spec-HalotoMinD-R	This paper	Oligo_ID:HF0248	ATAGCCTCACCCAACCCATTGGAAATCTCCAGAG
Sequence-based reagent	GC-3-HalotoMinD+Down-F	This paper	Oligo_ID:HF0249	AGATTTCCAATGGGTTGGGTGAGGCTATCGTAATAAC
Sequence-based reagent	GC-3-MinDDowntopUC18-R	This paper	Oligo_ID:HF0250	CATGATTACGAATTCGAGCTCGGTACCCGCCTACTTCAATCTGCTGTTC
Sequence-based reagent	MinD-G12V-QC-F	This paper	Oligo_ID:HF0213	TCGGGAAAAGtCGGAGTAGGT
Sequence-based reagent	MinD-G12V-QC-R	This paper	Oligo_ID:HF0214	AGTTATTACGATAGCCTCAC
Sequence-based reagent	MinD-K16A-QC-F	This paper	Oligo_ID:HF0215	CGGAGTAGGTgcGACAACAACATC
Sequence-based reagent	MinD-K16A-QC-R	This paper	Oligo_ID:HF0216	CCTTTTCCCGAAGTTATTAC
Sequence-based reagent	MinD-I260E-QC-F	This paper	Oligo_ID:HF0237	GATGGCTAAGgaaAAGTCATTTTTCGG
Sequence-based reagent	MinD-I260E-QC-R	This paper	Oligo_ID:HF0238	ATTCCTTTGTTTTGCTCTTC
Sequence-based reagent	MinD-D40A-QC-F	This paper	Oligo_ID:HF0239	AGTAGATACTgcgATAGGACTGC
Sequence-based reagent	MinD-D40A-QC-R	This paper	Oligo_ID:HF0240	AAGCATACGCGCTTC
Sequence-based reagent	MinJ-PDZ-28a-BamHI-F	This paper	Oligo_ID:HF0255	CATGGATCCTTGGAGGTCTTGTTCCAGGGACCGCGTATTTTTCTG
Sequence-based reagent	MinJ-PDZ-28a-XhoI-R	This paper	Oligo_ID:HF0256	CGCCTCGAGTTATTATGATCCCGAAGCGAC
Peptide, recombinant protein	Gel filtration standard	Bio-Rad	Cat#: 1511901	Used for SEC calibration
Commercial assay or kit	EnzChek Phosphate Assay Kit	Thermo Fisher Scientific	Cat#: E6646	ATP hydrolysis analysis
Commercial assay or kit	Q5 Site-Directed Mutagenesis Kit	New England Biolabs	Cat#: E0554	
Commercial assay or kit	NEBuilder HiFi DNA Assembly Cloning Kit	New England Biolabs	Cat#: E2621	
Software, algorithm	AlphaFold2	Jumper et al., 2021	https://doi.org/10.1038/s41586-021-03819-2; RRID:SCR_025454	Structural modeling of MinD
Software, algorithm	AlphaFold3	Abramson et al., 2024	https://doi.org/10.1038/s41586-024-07487-w; RRID:SCR_028034	Structural modeling of MinJ
Software, algorithm	Fiji (ImageJ2)	Schindelin et al., 2012	https://doi.org/10.1038/nmeth.2019; RRID:SCR_002285	Version 1.53 g
Software, algorithm	Oufti	Paintdakhi et al., 2016	https://doi.org/10.1111/mmi.13264; RRID:SCR_016244	Manual cell outlines for SMTracker 2.0
Software, algorithm	SMTracker 2.0	Oviedo-Bocanegra et al., 2021	https://doi.org/10.1093/nar/gkab696	SMLM track analysis
Software, algorithm	MicrobeJ	Ducret et al., 2016	https://doi.org/10.1038/nmicrobiol.2016.77; RRID:SCR_023914	Version 5.11c
Software, algorithm	TrackMate	Tinevez et al., 2017	https://doi.org/10.1016/j.ymeth.2016.09.016	Version 6.0.1
Software, algorithm	R Studio	Posit	RRID:SCR_000432	Version 2023.12.1+402
Software, algorithm	GraphPad Prism 9	GraphPad Software	RRID:SCR_002798	Version 9.4.0, data visualization and analysis
Software, algorithm	Zen Blue/Zen Black	Zeiss	RRID:SCR_013672	Image acquisition and SR analysis
Software, algorithm	BLItz Pro	Sartorius	Version 1.3.1.3	Kinetic analysis and fitting
Software, algorithm	Tecan i-control	Tecan	Version 2.0	Control for Infinite 200 Pro
Software, algorithm	R (4.2.2)	R Development Core Team, 2022	https://www.R-project.org/; RRID:SCR_001905	Version 4.2.2
Other	Streptavidin (SA) Biosensors	Sartorius	Cat#: 18-5019	Used with BLItz platform
Other	HaloTag TMR Ligand	Promega/Zeiss	Cat#: G8251	HaloTag staining
Other	NileRed	Thermo Fisher/Invitrogen	Cat#: N1142	Cell membrane staining (1 µg ml^–1^)
Other	Ni-NTA column (Protino)	Macherey-Nagel	Cat#: 745400.1	Protein affinity purification
Other	Superdex 200 Increase 10/300 GL	Cytiva	Cat#: 28990944	Size-exclusion chromatography
Other	Nuclepore Track-Etched Membrane	Whatman	Cat#: 10419506	Polycarbonate membrane (0.1 µm)
Other	Gene Frames	Thermo Scientific	Cat#: AB0577	1.5×1.6 cm
Other	Amicon Ultra filter devices 10 kDa MWCO	Millipore; Merck	Cat#: UFC901008	Protein concentration
Other	*E. coli* total extract (lipids)	Avanti Research	Cat#: 100500C	Liposome preparation
Other	DSPE-PEG(2000)-biotin	Avanti Research	Cat#: 880129	Liposome preparation

### Bacterial strains, plasmids, and oligonucleotides

Plasmid and strain construction of all relevant constructs is described in Appendix 1. Oligonucleotides, plasmids, and strains used in this study are listed in Supplementary file 1, Supplementary file 2, and Supplementary file 3, respectively, as well as in the Key resources table.

### Media and growth conditions

*E. coli* was grown on lysogeny broth (LB) agar plates containing 10 g l^–1^ tryptone, 5 g l^–1^ yeast extract, 10 g l^–1^ NaCl, and 1.5% (wt/vol) agar at 37°C overnight using appropriate selection (kanamycin 50 µg ml^–1^, chloramphenicol 35 µg ml^–1^).

*B. subtilis* was grown on nutrient agar plates using commercial nutrient broth and 1.5% (wt/vol) agar at 37°C overnight. To reduce inhibitory effects, antibiotics were only used for transformations and when indicated, since allelic replacement is stable after integration (kanamycin 5 µg ml^–1^, spectinomycin 100 µg ml^–1^).

For microscopy, *B. subtilis* was inoculated to an OD_600_ 0.05 from a fresh overnight culture and grown in MD medium – a modified version of Spizizen Minimal Medium (Anagnostopoulos and Spizizen, 1961) – at 37°C with aeration in baffled shaking flasks (200 rpm) to OD_600_ 0.5–0.8. MD medium contains 10.7 mg ml^−1^ K_2_HPO_4_, 6 mg ml^−1^ KH_2_PO_4_, 1 mg ml^−1^ Na_3_ citrate, 20 mg ml^−1^ glucose, 50 µg ml^−1^ L-tryptophan, 11 µg ml^−1^ ferric ammonium citrate, 2.5 mg ml^−1^ L-aspartate, and 0.36 mg ml^−1^ MgSO_4_ and was always supplemented with 1 mg ml^−1^ casamino acids. Subsequently, cultures were diluted to OD_600_ 0.05 in fresh, pre-warmed MD medium and grown to OD_600_ 0.5 (exponential phase).

### Purification of His-MinC, His-PDZ, His-MinD, and variants

For heterologous expression of His-MinC, His-PDZ, and His-MinD and its variants, freshly transformed BL21(DE3) cells carrying the respective pET-28a expression vector (Supplementary file 2), as well as pLysS (His-MinC, His MinD, and variants only), were transferred to LB medium with appropriate selection (kanamycin 50 µg ml^–1^; chloramphenicol 35 µg ml^–1^ in the presence of pLysS) and grown overnight (37°C, 200 rpm). The next day, fresh LB medium was inoculated 1:150 from the overnight culture and grown to an OD_600_ of 0.5 at 37°C and 200 rpm. Since His-PDZ tended to form inclusion bodies under these conditions, its expression culture was instead grown at 18°C and 120 rpm without IPTG induction. For all other constructs, protein expression was induced with 1 mM IPTG. Cells expressing His-MinC and all His-MinD variants were harvested after 3 hr of induction, whereas His-PDZ cultures were collected after overnight incubation. Cells were harvested by centrifugation (4°C, 5000×*g*, 15 min) and stored at –80°C. For purification, a protocol of *E. coli* MinD purification was adapted from Ramm et al., 2018: the cell pellet was resuspended on ice in lysis buffer (50 mM NaH_2_PO_4_, 500 mM NaCl, and 10 mM imidazole; pH 8.0), supplemented with protease inhibitor cocktail (Roche), 0.2 mM Mg^2+^-ADP, and 0.4 mM TCEP (His-MinD and variants only) and ~250 U mL^–1^ DNase I (Roche), and subsequently lysed in a pre-cooled (4°C) French pressure cell (Amico; 3×1200 psi). The debris was then removed via centrifugation (10,000×*g*, 30 min, 4°C), and the supernatant was passed through a 1 ml Ni-NTA column (Protino, Macherey-Nagel) utilizing liquid chromatography (ÄKTApurifier). Here, the resin was washed with 10 ml binding buffer A (50 mM NaH_2_PO_4_, 500 mM NaCl, 20 mM imidazol; pH 8) before elution buffer B (50 mM NaH_2_PO_4_, 500 mM NaCl, 250 mM imidazol; pH 8 [and 0.2 mM Mg^2+^-ADP and 0.4 mM TCEP for His-MinD and variants]) was added sequentially at concentrations of 5% (10 ml), 10% (20 ml), and finally 100% (10 ml). Recombinant protein eluate was collected in 0.5 ml fractions. Fractions containing high concentrations of protein (determined by chromatogram analysis, sodium dodecyl sulfate-polyacrylamide gel electrophoresis [SDS-PAGE], and western blotting using Penta-His antibody [QIAGEN]) were pooled and concentrated using Amicon filter devices with 10 kDa molecular weight cutoff (Millipore; Merck). Additionally, protein aggregates were removed by pelleting them through centrifugation (10,000×*g*, 10 min, 4°C).

Samples were further purified by SEC through a Superdex200 Increase 10/300 GL column (Cytiva) at a flow rate of 0.75 ml min^–1^ using gel filtration buffer C (50 mM HEPES-KOH, 150 mM KCl, 10% [vol/vol] glycerol, 0.1 mM EDTA, pH 7.2). For His-MinD and its variants, buffer C was supplemented with 0.4 mM TCEP and 0.2 mM Mg²^+^-ADP immediately before use. His-PDZ was not subjected to SEC because it could not be recovered after SEC. Instead, it was used directly following affinity purification. To calibrate the column and allow identification of relevant fractions, a gel filtration standard (Bio-Rad) was run according to the manufacturer’s protocol and analyzed using the same conditions, respectively. Fractions containing recombinant protein were pooled and either directly used for the respective assays, stored at 4°C for a maximum of 1 week, or flash-frozen and stored at –80°C. Final protein concentration was determined using Bradford assay with BSA as standards. Protein purity and stability were confirmed via SDS-PAGE using 12% polyacrylamide gels and western blotting using a Penta-His antibody (QIAGEN) (Figure 1—figure supplement 1, Figure 2—figure supplement 1, Figure 2—figure supplement 2, Figure 4—figure supplement 1, Figure 4—figure supplement 2, Figure 4—figure supplements 3 and 4).

### ATP hydrolysis assays

ATP hydrolysis was analyzed in continuous reactions using the EnzChek Phosphate Assay Kit (Thermo Fisher Scientific), according to the manufacturer’s manual. Reaction volumes were reduced to 100 µl and assayed in flat-bottom 96-well plates (Greiner-UV-Star 96-well plates). Reactions were measured at 37°C continuously every minute over a time-course of 3 hr in a Tecan Infinite 200 Pro with the Software Tecan i-control version 2.0. If not indicated differently, the reactions contained either 0–16 µM His-MinD or the respective variant/protein combination, as well as 2 mM Mg^2+^-ATP and 0.2 mg ml^–1^ liposomes. Liposomes were made from *E. coli* total extract (Avanti) by first drying and then resuspending and rehydrating lipids in gel filtration buffer C (50 mM HEPES-KOH, 150 mM KCl, 10% [vol/vol] glycerol, 0.1 mM EDTA). Next, the lipids were extruded 40 times through 100 nm polycarbonate membranes (Nuclepore Track-Etched Membrane 0.1 µm, Whatman). Before starting the reactions with 2 mM Mg^2+^-ATP, the plate was pre-incubated for 10 min in order to eliminate phosphate contamination.

Data were first analyzed in Excel (normalization, subtraction of ATP autohydrolysis, and subtraction of the no-substrate control), and a linear regression was used to determine the hydrolysis rate per minute. Data visualization and further analysis were performed using GraphPad Prism version 9.4.0 for Windows (GraphPad Software). Statistical comparisons between His-MinD alone (*n*=2) and His-MinC/His-PDZ conditions (*n*=3 each) were performed using Welch’s t-test (two-tailed), which is appropriate for unequal variances and unequal sample sizes.

### AlphaFold prediction and structure-based analysis

A structural model of *B. subtilis* MinD was calculated using AlphaFold2 (Jumper et al., 2021) installed on a local computer (Figure 3A), where the structural homology search was performed against the full AlphaFold database as of 11.02.2024 (Jumper et al., 2021).

A structural model of *B. subtilis* MinJ was calculated using AlphaFold3 (Abramson et al., 2024), where the structural homology search was performed against the full AlphaFold database as of 01.02.2025 (Abramson et al., 2024).

### Bio-layer interferometry

Measurements were performed on the BLItz platform (Sartorius) using Streptavidin (SA) Biosensors (Sartorius, 18-5019). The BLItz device was operated with the Software BLItz Pro (version 1.3.1.3) using the advanced kinetics protocol modified as shown in Supplementary file 4 and essentially as described before (Weiß et al., 2023; Maximova et al., 2019; Wallner et al., 2017).

Beforehand, liposomes were made from a 33:1 mixture of *E. coli* total extract (Avanti) and DSPE-PEG_(2000)_-biotin (Avanti) by first drying and then resuspending and rehydrating lipids in gel filtration buffer C (50 mM HEPES-KOH, 150 mM KCl, 10% [vol/vol] glycerol, 0.1 mM EDTA). Next, the lipids were extruded 40 times through 100 nm polycarbonate membranes (Nuclepore Track-Etched Membrane 0.1 µm, Whatman). Biosensors were hydrated for at least 10 min in a 96-well plate in 200 µl gel filtration buffer C.

As a starting point, unspecific binding was tested. For this, non-biotinylated His-MinD was used to be loaded onto the Streptavidin biosensor. For the binding assays, all measurements were performed in black reaction tubes with 200 µl gel filtration buffer C or the indicated solution (Supplementary file 4). After measuring the initial baseline, biotinylated liposomes were allowed to bind the biosensor. Another baseline was measured in the same tube as before. For association, 200 µl solutions of His-MinD and variants in different concentrations and/or in combination with His-MinC or His-PDZ, respectively, were exposed to the sensor, first without and later with the addition of 2 mM Mg-^2+^ATP (final concentration) just before use. Furthermore, unspecific binding to liposome-saturated sensors was tested using BSA (16 µM). For dissociation, the sensor was exposed to buffer again. Each measurement was performed with a fresh biosensor.

For BLI experiments involving His-MinD and one of its direct interactors, we first assessed whether His-MinC or His-PDZ can directly interact with His-MinD in vitro. To this end, a liposome-coated BLI sensor was first dipped into an 8 µM His-MinD solution. Subsequently, the sensor was exposed to solutions containing either 8 µM His-MinD together with 8 µM His-MinC or 8 µM His-PDZ, respectively. As a negative control, a sample containing 8 µM His-MinD and 8 µM BSA was used. Both His-MinC and His-PDZ induced a significant increase in the equilibrium binding signal upon interaction with immobilized His-MinD, consistent with specific complex formation. In contrast, the BSA control showed only a minimal signal increase, confirming the specificity of the observed binding. To further validate the sensor specificity, individual proteins were tested. Only His-MinD exhibited robust binding to the liposome-coated sensor, while His-MinC and His-PDZ showed negligible signal, indicating nonspecific interactions (Figure 5—figure supplement 1). These initial tests support the expected specificity of the MinD:MinC and MinD:PDZ interactions.

Kinetic analysis and fitting were performed by the BLItz Pro Software (version 1.3.1.3). Data was exported and visualized using GraphPad Prism version 9.4.0 for Windows (GraphPad Software).

The BLItz Pro Software analysis follows a 1:1 binding model, where:

$$\displaystyle A + B \xrightleftharpoons[k_{\mathrm{off}}]{k_{\mathrm{on}}} AB$$

with *k*_on_ = rate of association or ‘on-rate’ and *k*_off_ = rate of dissociation or ‘off-rate’.

Formula for fitting the association (*a*=slope):

$$\displaystyle Y=Y_{0}+a\,(1- e^{- k_{\rm obs}\times t}) $$

$$\displaystyle k_{\rm obs}=k_{\rm on}\times \left [A\left (M\right)\right ]+k_{\rm off}$$

Formula for fitting the dissociation:

$$\displaystyle Y=Y_{0}+a\times e^{- k_{\rm off}\times t}$$

Resulting in the equilibrium dissociation constant *K*_D_:

$$\displaystyle K_{D}=\frac{k_{\rm off}}{k_{\rm on}}$$

### Fluorescence microscopy

#### Microscopy slide preparation

For epifluorescence imaging, *B. subtilis* cells were mounted on 1.5% MD agarose pads using 1.5×1.6 cm ‘Gene Frames’ (Thermo Scientific) and incubated 10 min at 37°C before microscopic analysis.

For SMLM, slides and coverslips were first cleaned by overnight storage in 1 M KOH, carefully rinsed with ddH_2_O, and subsequently dried with pressurized air. Next, 1.5% (wt/vol) low melting agarose (Sigma-Aldrich) was dissolved in MD medium. Medium was sterile-filtered (0.2 µm pore size) shortly before being used to remove particles. To produce flat, uniform, and reproducible agarose pads, 1.5×1.6 cm ‘Gene Frames’ (Thermo Fisher) were utilized, and pads were allowed to solidify for 1 hr at room temperature to be used within the next 3 hr.

#### Staining of HaloTag

For staining, 1 ml of bacterial culture was moved to a 2 ml microcentrifuge tube and incubated with 5 nM (SMLM) or 50 nM (epifluorescence) HaloTag TMR ligand at 37°C for 10 min. Cells were harvested (4000×*g*, 3 min, 37°C) and washed four times in pre-warmed MD medium, before being mounted on the previously described microscopy slides.

#### Epifluorescence imaging

For strain characterization (Figure 3B), microscopy images were taken on an Axio Observer 7 microscope (Zeiss) equipped with a Colibri 7 LED light source (Zeiss) and an OrcaR^2^ camera (Hamamatsu) using a Plan-Apochromat 100×/1.4 oil Ph3 objective (Zeiss). TMR fluorescence was visualized with a 90 HE LED filter set (QBP 425/30+514/30+592/30+709/100) (Zeiss) and excited at 555 nm (100% intensity of Colibri 7 LED) for 400 ms. The microscope was equipped with an environmental chamber set to 37°C. Digital images were acquired with Zen Blue (Zeiss) and analyzed and edited using Fiji (Schindelin et al., 2012) and ImageJ2 (Rueden et al., 2017). For measurement of cell length and minicells, cells were stained with NileRed (1 µg ml^–1^ final concentration) at OD_600_ 0.5 and incubated for a further 20 min (200 rpm, 37°C) before mounting them on an agarose pad. To minimize bias during analysis, cell lengths and number of minicells were identified using the Fiji (Schindelin et al., 2012) plugin MicrobeJ 5.11c (Ducret et al., 2016) on phase contrast images with manual correction by screening the results against the simultaneously acquired membrane stain images and sorting out mislabeled cells. For cell length measurements, MicrobeJ modules *options*, *exclude on edges*, *shape descriptor*, *segmentation,* and *chain* were used with default settings, except for the following parameters in *options* (*area* 1-max; *length* 1-max; *width* 0.6–1; *sinuosity* 1-max; *angularity* 0–0.5) and *chain* (*tolerance* 0.1; *area* 1.5-max). To identify minicells, MicrobeJ modules *options*, *exclude on edges*, *shape descriptor,* and *segmentation* were used with default settings, except for the following parameters in *options* (*area* 0.15–1; *length* 0.5–1.2; *width* 0.3–1; *circularity* 0.95-max).

#### SMLM imaging

SMLM imaging was performed with an Elyra 7 (Zeiss) inverted microscope equipped with two pco.edge sCMOS 4.2 CL HS cameras (PCO AG), connected through a DuoLink (Zeiss), only one of which was used in this study. Cells were observed through an alpha Plan-Apochromat 63×/1.46 Oil Korr M27 Var2 objective, yielding a final pixel size of 97 nm. During image acquisition, the focus was maintained with the help of a Definite Focus.2 system (Zeiss). Fluorescence was excited with a 561 nm (100 mW) laser, and signals were observed through a multiple beam splitter (405/488/561/641 nm) and laser block filters (405/488/561/641 nm) followed by a Duolink SR QUAD (Zeiss) filter module (secondary beam splitter: LP 560, emission filters: EF BP420−480+BP495–550).

Cells were illuminated with the 561 nm laser (50% intensity) in TIRF mode (62° angle). For each time-lapse series, 10,000 frames were taken with 20 ms exposure time (~24 ms with transfer time included) and 50% 561 nm intensity laser.

#### SMLM analysis

For average localization analysis (Figure 6A), a single-molecule localization table was constructed from a 2D Gaussian fitting in the ZEN 3.0 SR (black) software (Zeiss) with a peak mask size of 9 and a peak intensity-to-noise ratio of 6, and further allowing molecules to overlap. Filtering was used to minimize noise, background, and out-of-focus emitters, discarding events that did not emit within a point spread function size of 100–200 nm and did not display a photon count of 50–600. Further analysis was performed using individual Fiji (Schindelin et al., 2012) scripts for the cell outlines and R (4.2.2) scripts in R Studio (2023.12.1+402) for the corresponding localization events (R Development Core Team, 2022; team, 2024). Here, the remaining events were transformed to align the orientation of all cells in which the signals were detected, to be able to summarize all recorded localizations in one normalized cell per strain. The localization frequency is displayed as a heatmap using the gradual viridis scaling from the ggplot2 R package (Wickham, 2016), where the highest frequency (normalized frequency 1) is displayed in yellow and the lowest frequency (normalized frequency 0) in blue (Figure 6A). The script used to create these heatmaps from SMLM tables was previously published in Peng et al., 2025.

For SMT, spots were identified with the LoG Detector of TrackMate version 6.0.1 (Tinevez et al., 2017) implemented in Fiji 1.53 g (Schindelin et al., 2012), an estimated diameter of 0.5 μm, and median filter and sub-pixel localization activated. The signal-to-noise threshold for the identification of the spots was set at 7. To limit the detection of particles to single-molecules, frames belonging to the bleaching phase of TMR were removed individually from the time lapses prior to the identification of spots. Spots were merged into tracks via the ‘Simple LAP Tracker’ of TrackMate, with a maximum linking distance of 500 nm and no frame gaps allowed. Only tracks with a minimum length of 5 frames were used for further analysis, yielding a minimum number of total tracks per sample of 2596.

To identify differences in protein mobility and/or behavior, the resulting tracks were subjected to SLA, MSD, and SQD analysis in SMTracker 2.0 (Oviedo-Bocanegra et al., 2021), which first required determining cell meshes manually using Oufti (Paintdakhi et al., 2016). SLA was performed with a confinement circle radius of 97 nm and a minimum number of five steps to be considered static. The average MSD was calculated for four separate time points per strain (exposure of 20 ms: *τ*=24, 48, 72, and 96 ms), followed by fitting of the data to a linear equation. The last time point of each track was excluded to avoid track-ending-related artifacts. The cumulative probability distribution of the SQD was used to estimate the diffusion constants and relative fractions of up to three diffusive states. Diffusion constants were determined simultaneously for the compared conditions, therefore allowing a more direct comparison of population fractions. Additionally, the analysis was performed for each strain non-comparatively, yielding individual diffusion coefficients, as displayed in Table 4.

## Data Availability

All data generated in this study are included in the manuscript, the supplementary material or the source data files. The single-molecule localization microscopy source data file has been deposited on Dryad: https://doi.org/10.5061/dryad.95x69p91b. All strains and plasmids are available on request. The following dataset was generated: FeddersenH DyckmansC BramkampM 2026Membrane Binding Controls the ATPase Cycle and Localization of MinD in *Bacillus subtilis*Dryad Digital Repository10.5061/dryad.95x69p91b42258376

## Additional files

Supplementary file 1. Oligonucleotides used in this study.

Supplementary file 2. Plasmids used in this study.

Supplementary file 3. Strains used in this study.

Supplementary file 4. Bio-layer interferometry (BLI) advanced kinetics protocol.

MDAR checklist

Source data 1. Source data underlying most figures and tables in this paper.

## Appendix 1

### Plasmid and strain construction

All plasmids generated in this study were subjected to sequencing of relevant regions to verify plasmid integrity and to avoid occurrence of mutations. The oligonucleotides, plasmids, and strains used in this study are listed in Supplementary files 1–3, respectively. *E. coli* NEB5-alpha was used to amplify and maintain plasmids.

### *E. coli* overexpression vector construction and transformation

For protein expression, the respective pET-28a plasmid variant was freshly transformed into BL21(DE3)/pLysS the day before expression using appropriate selection.

pET-28a-*minD* (NAP013). The *minD* gene was PCR-amplified from *B. subtilis* 168 gDNA using primers NA018 and NA019. Subsequently, pET-28a (+) and the purified PCR product were digested using NdeI and XhoI and afterward ligated and transformed.

pET-28a-*minD*-G12V (EHF045). This plasmid was generated using the Q5 Site-Directed Mutagenesis Kit (New England Biolabs) on plasmid NAP013 in combination with primers HF0213 and HF0214.

pET-28a-*minD*-K16A (EHF046). This plasmid was generated using the Q5 Site-Directed Mutagenesis Kit (New England Biolabs) on plasmid NAP013 in combination with primers HF0215 and HF0216.

pET-28a-*minD*-I260E (EHF047). This plasmid was generated using the Q5 Site-Directed Mutagenesis Kit (New England Biolabs) on plasmid NAP013 in combination with primers HF0237 and HF0238.

pET-28a-*minD*-D40A (EHF052). This plasmid was generated using the Q5 Site-Directed Mutagenesis Kit (New England Biolabs) on plasmid NAP013 in combination with primers HF0239 and HF0240.

pET-28a-*minC* (NAP012). The *minC* gene was PCR-amplified from *B. subtilis* 168 gDNA using primers NA016 and NA017. Subsequently, pET-28a (+) and the purified PCR product were digested using NdeI and XhoI and afterward ligated and transformed.

pET-28a-PDZ (CD001). The truncated *minJ* gene containing the PDZ domain sequence was PCR-amplified from *B. subtilis* 168 gDNA using primers HF0255 and HF0256. Subsequently, pET-28a (+) and the purified PCR product were digested using BamHI and XhoI and afterward ligated and transformed.

### *B. subtilis* integration plasmid construction, transformation, and selection of Halo-MinD constructs

Plasmid construction was verified via individual control digestion and DNA sequencing. Correct plasmids were transformed into *B. subtilis* strain 168 with the respectively indicated genetic background and selected for the introduced resistance. Resistant candidates were confirmed via PCR and microscopy.

pUC18-*minC-aad9-haloTag-minD-minDdown* (EHF054). This plasmid was constructed by assembling pUC18 (Norrander et al., 1983) with three PCR products encoding *minC* (Mahone and Goley, 2020), *aad9-haloTag* (3, encoding the spectinomycin adenyltransferase gene *aad9* and the *haloTag* gene), and *minD-minDdown* (4, encoding the *minD* gene and sequence downstream of *minD*) using the NEBuilder HiFi DNA Assembly Cloning Kit (New England Biolabs) according to protocol. The pUC18 plasmid was SmaI-digested and purified, while the other three assembly pieces were PCR-amplified. Specifically, the *minC* sequence with overhangs for pUC18 and *aad9* homology was amplified from gDNA template of 168 using primers HF0245 and HF0246. The *aad9-haloTag* sequence with overhangs for *minC* and *minD* homology was PCR-amplified from plasmid template EHF042 using primers HF0247 and HF0248. The *minDdown* sequence with overhangs for *minD* and pUC18 homology was PCR-amplified from gDNA template of 168 using primers HF0249 and HF0250.

pUC18-*minC-aad9-haloTag-minD-G12V-minDdown* (EHF055). This plasmid was generated using the Q5 Site-Directed Mutagenesis Kit (New England Biolabs) on plasmid EHF054 in combination with primers HF0213 and HF0214.

pUC18-*minC-aad9-haloTag-minD-K16A-minDdown* (EHF056). This plasmid was generated using the Q5 Site-Directed Mutagenesis Kit (New England Biolabs) on plasmid EHF054 in combination with primers HF0215 and HF0216.

pUC18-*minC-aad9-haloTag-minD-D40A-minDdown* (EHF057). This plasmid was generated using the Q5 Site-Directed Mutagenesis Kit (New England Biolabs) on plasmid EHF054 in combination with primers HF0239 and HF0240.

pUC18-*minC-aad9-haloTag-minD-I260E-minDdown* (EHF058). This plasmid was generated using the Q5 Site-Directed Mutagenesis Kit (New England Biolabs) on plasmid EHF054 in combination with primers HF0237 and HF0238.
