# Supplementary figures and images for: Membrane binding controls the ATPase cycle and localization of MinD in Bacillus subtilis
Source: eLife. 2026 Jun 8;13:RP101517. doi: 10.7554/eLife.101517 (PMC13246001; doi:10.7554/eLife.101517)

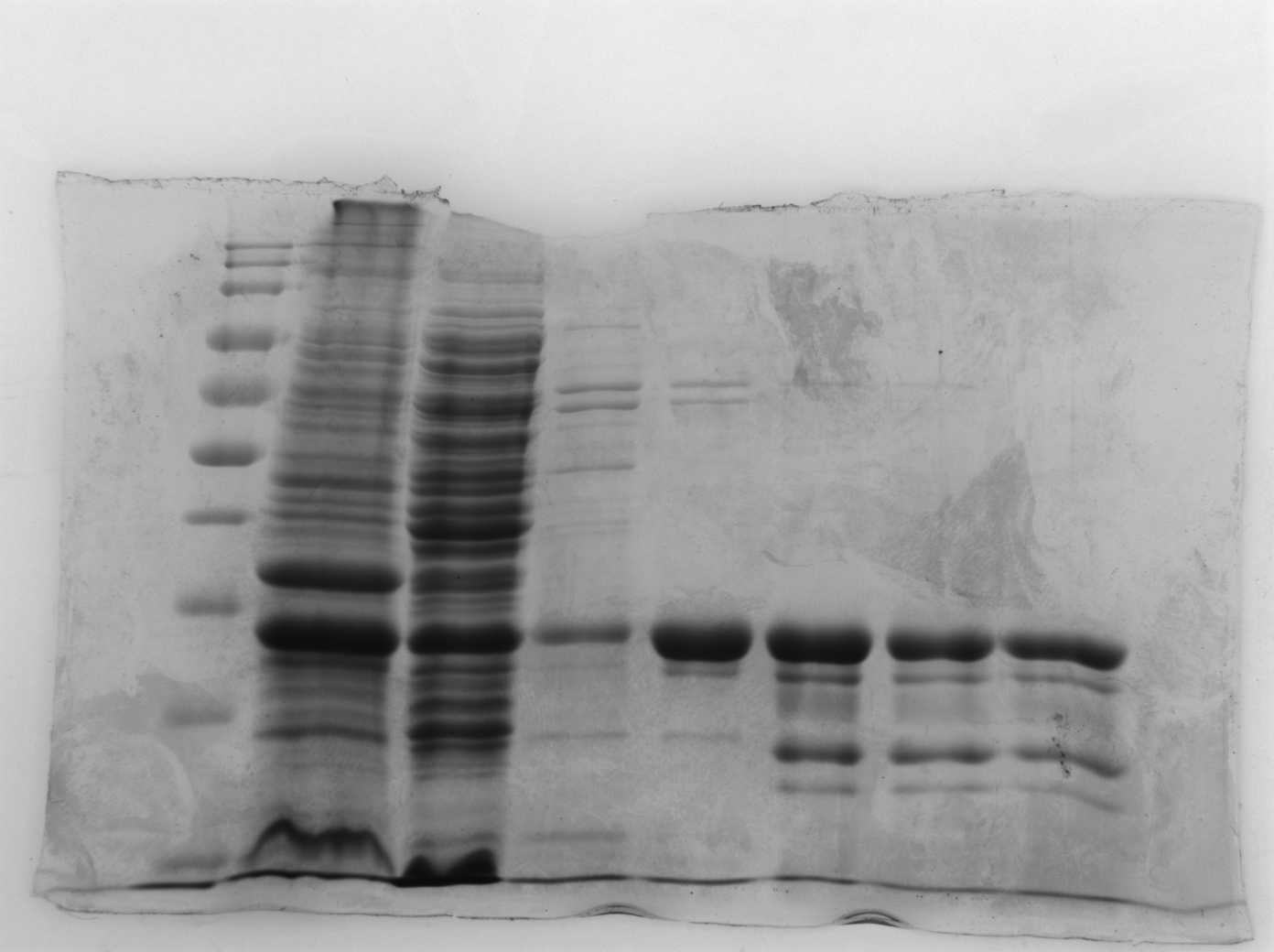

Supplement: Figure 1—figure supplement 1—source data 1. [file elife-101517-fig1-figsupp1-data1.zip › Figure 1—figure supplement 1-upper panel.tif]

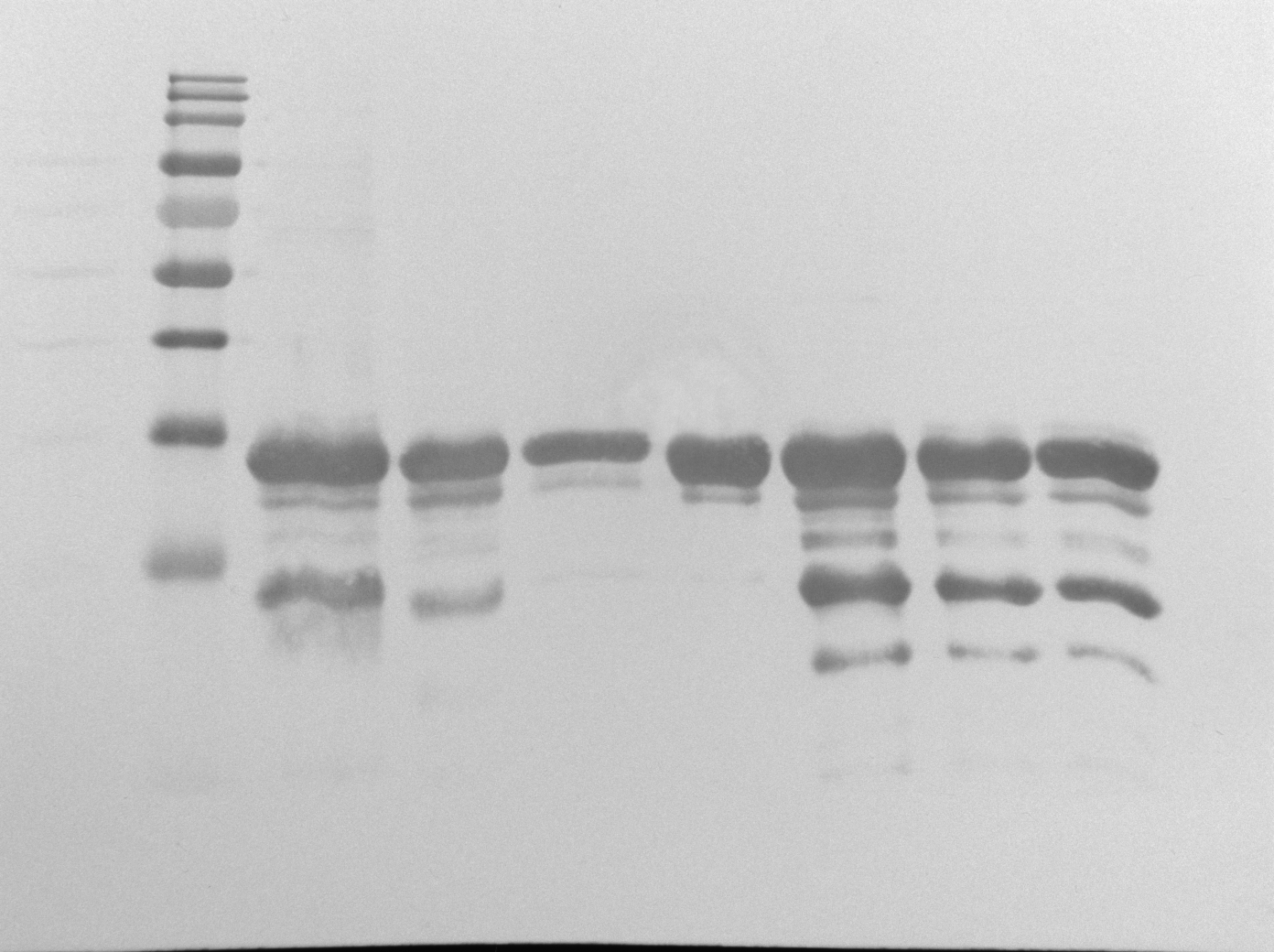

Supplement: Figure 1—figure supplement 1—source data 1. [file elife-101517-fig1-figsupp1-data1.zip › Figure 1—figure supplement 1-lower panel.tif]

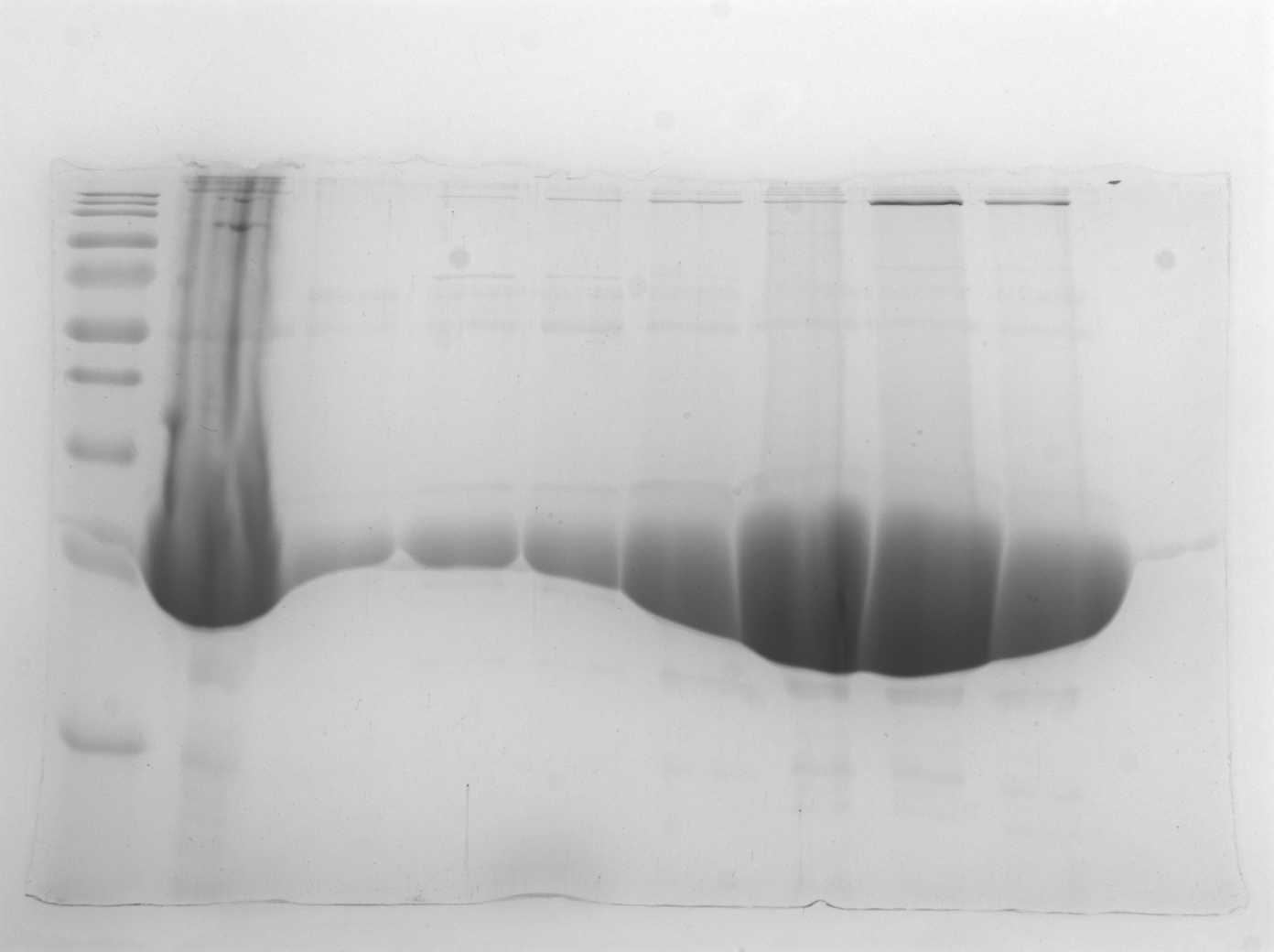

Supplement: Figure 2—figure supplement 1—source data 1. [file elife-101517-fig2-figsupp1-data1.zip › Figure 2–figure supplement 1-upper panel, right.tif]

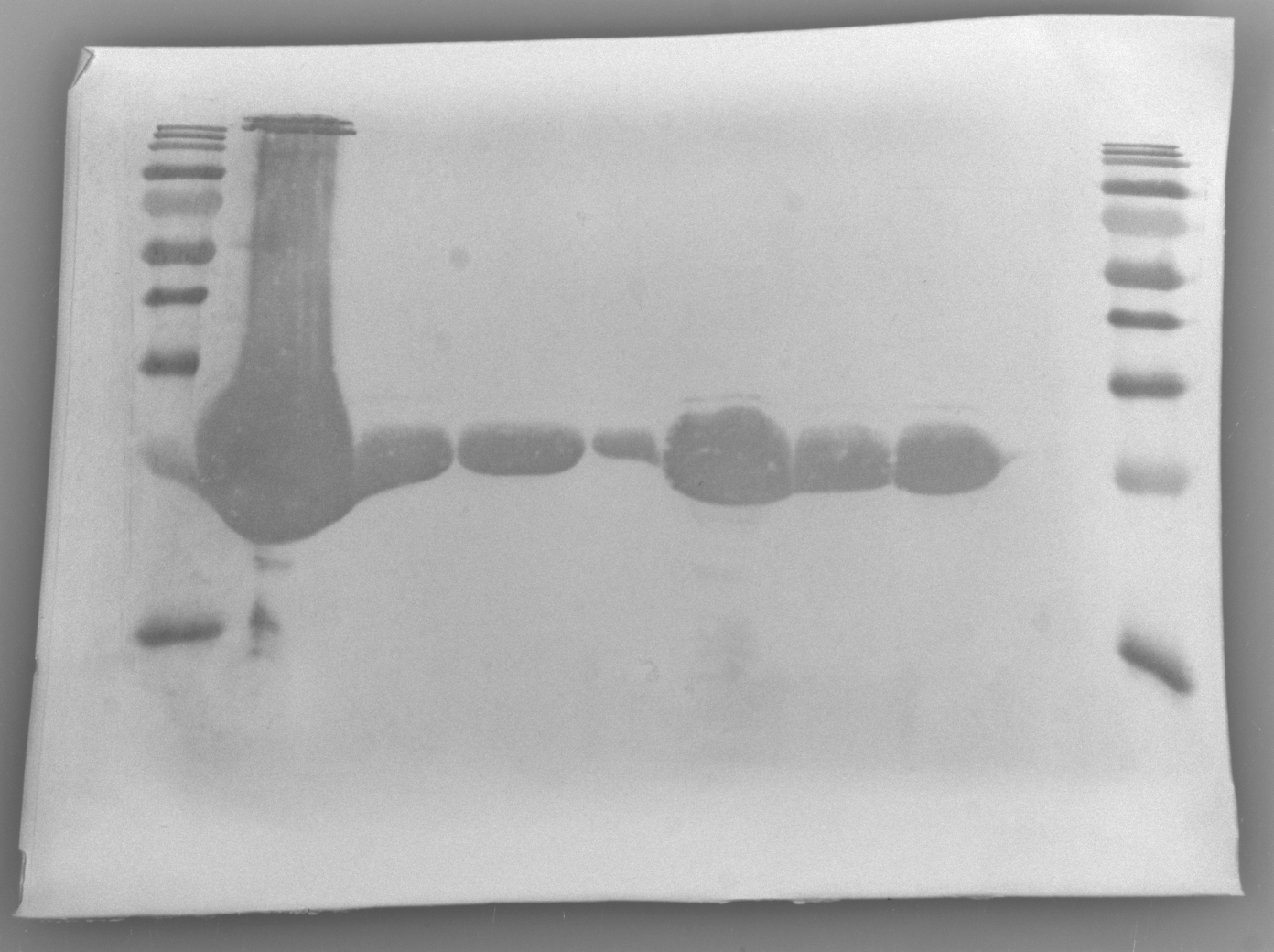

Supplement: Figure 2—figure supplement 1—source data 1. [file elife-101517-fig2-figsupp1-data1.zip › Figure 2–figure supplement 1-lower panel, left.tif]

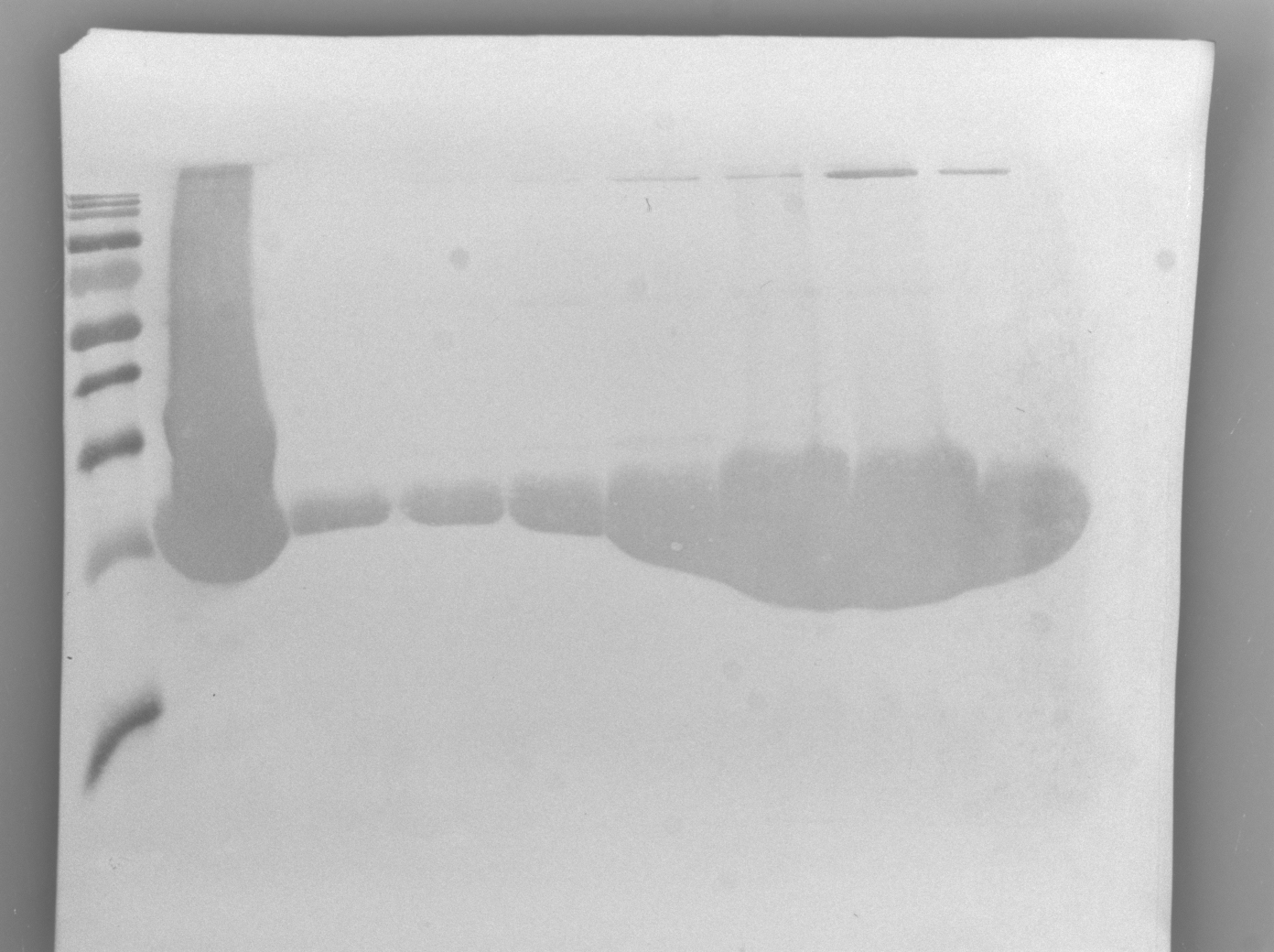

Supplement: Figure 2—figure supplement 1—source data 1. [file elife-101517-fig2-figsupp1-data1.zip › Figure 2–figure supplement 1-lower panel, right.tif]

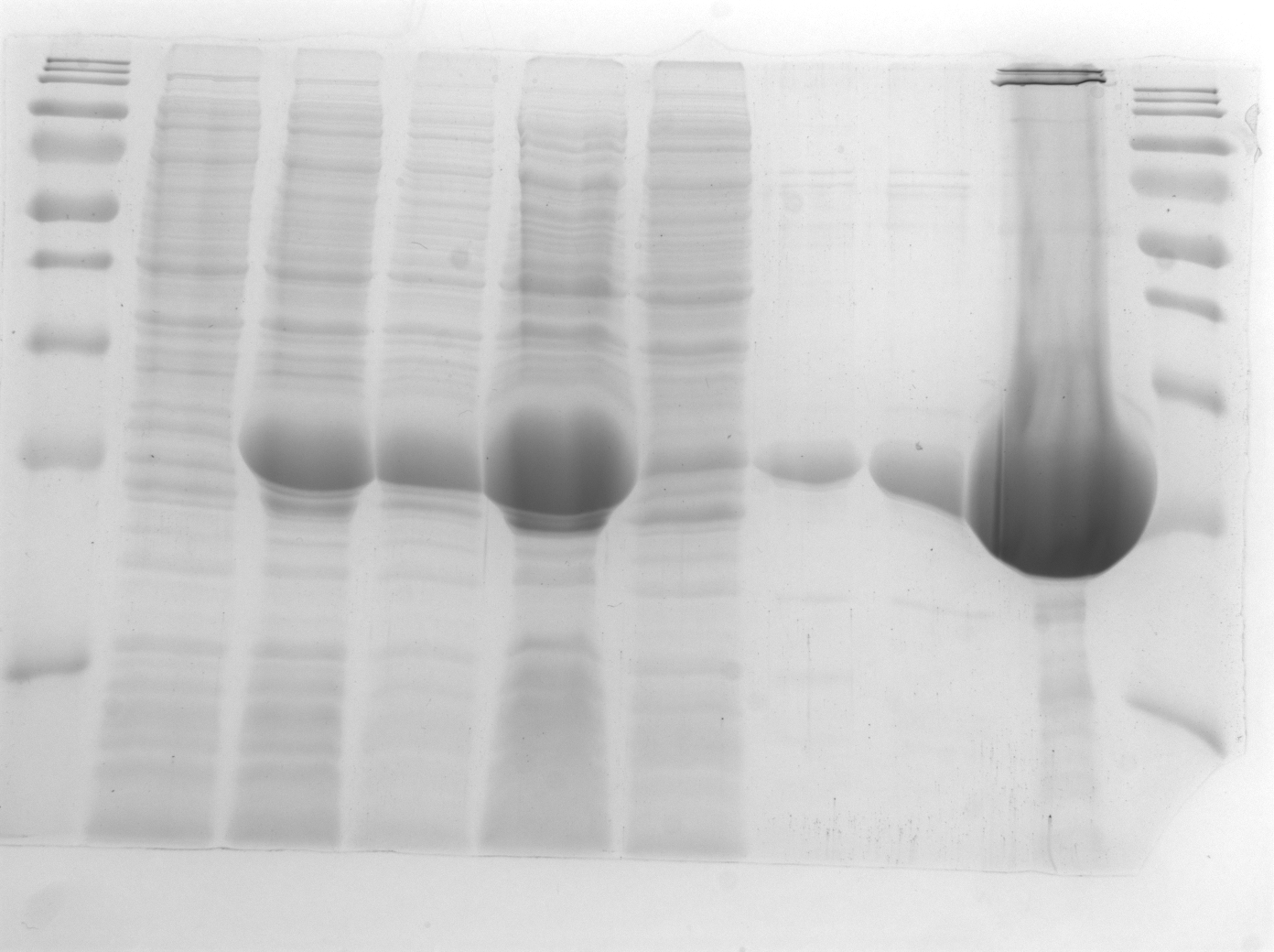

Supplement: Figure 2—figure supplement 1—source data 1. [file elife-101517-fig2-figsupp1-data1.zip › Figure 2–figure supplement 1-upper panel, left.tif]

Figure 2—figure supplement 1—source data 1

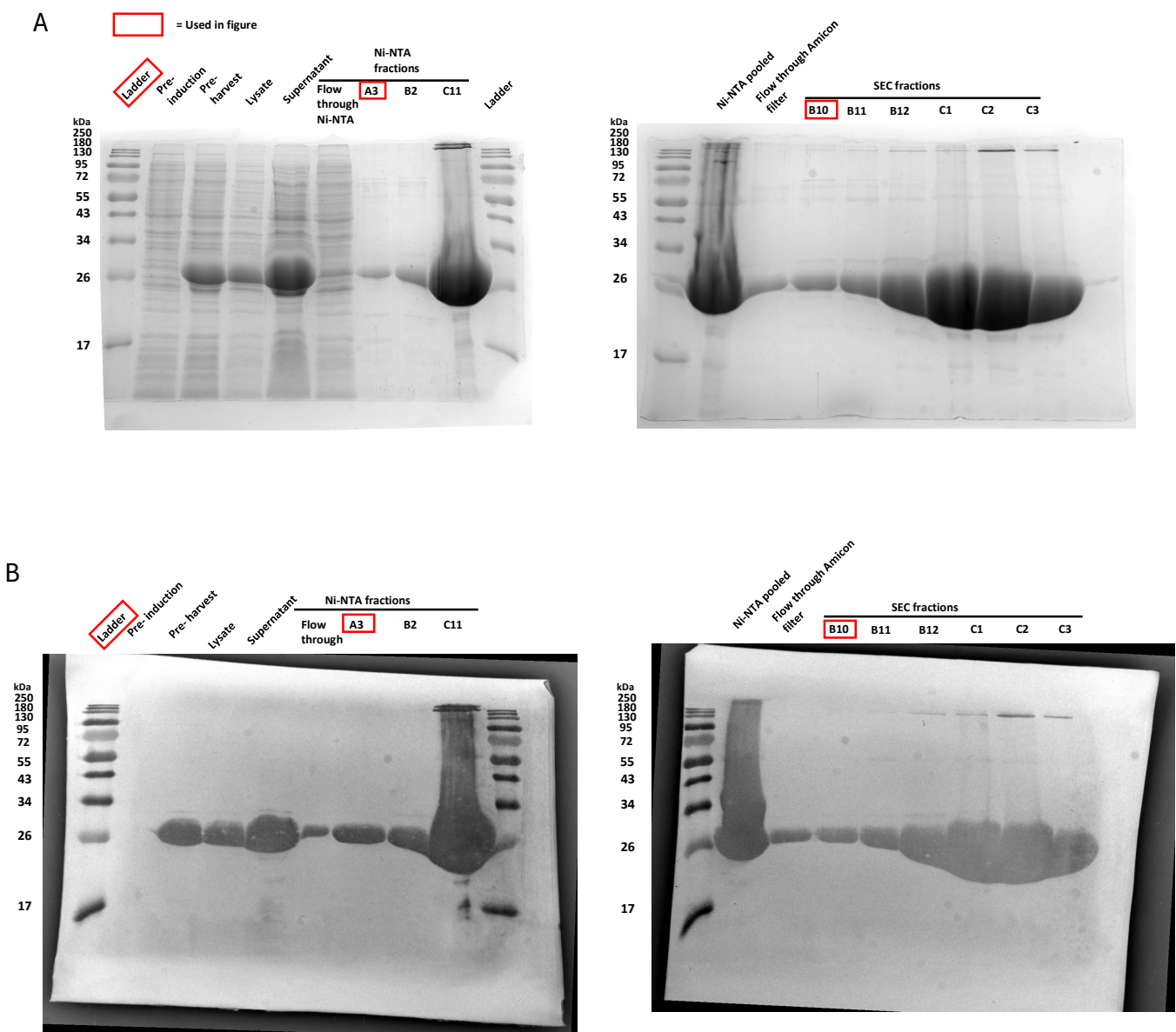

Supplement: Figure 2—figure supplement 1—source data 2. [file elife-101517-fig2-figsupp1-data2.zip › Figure 2–figure supplement 1-source data 1.pdf]

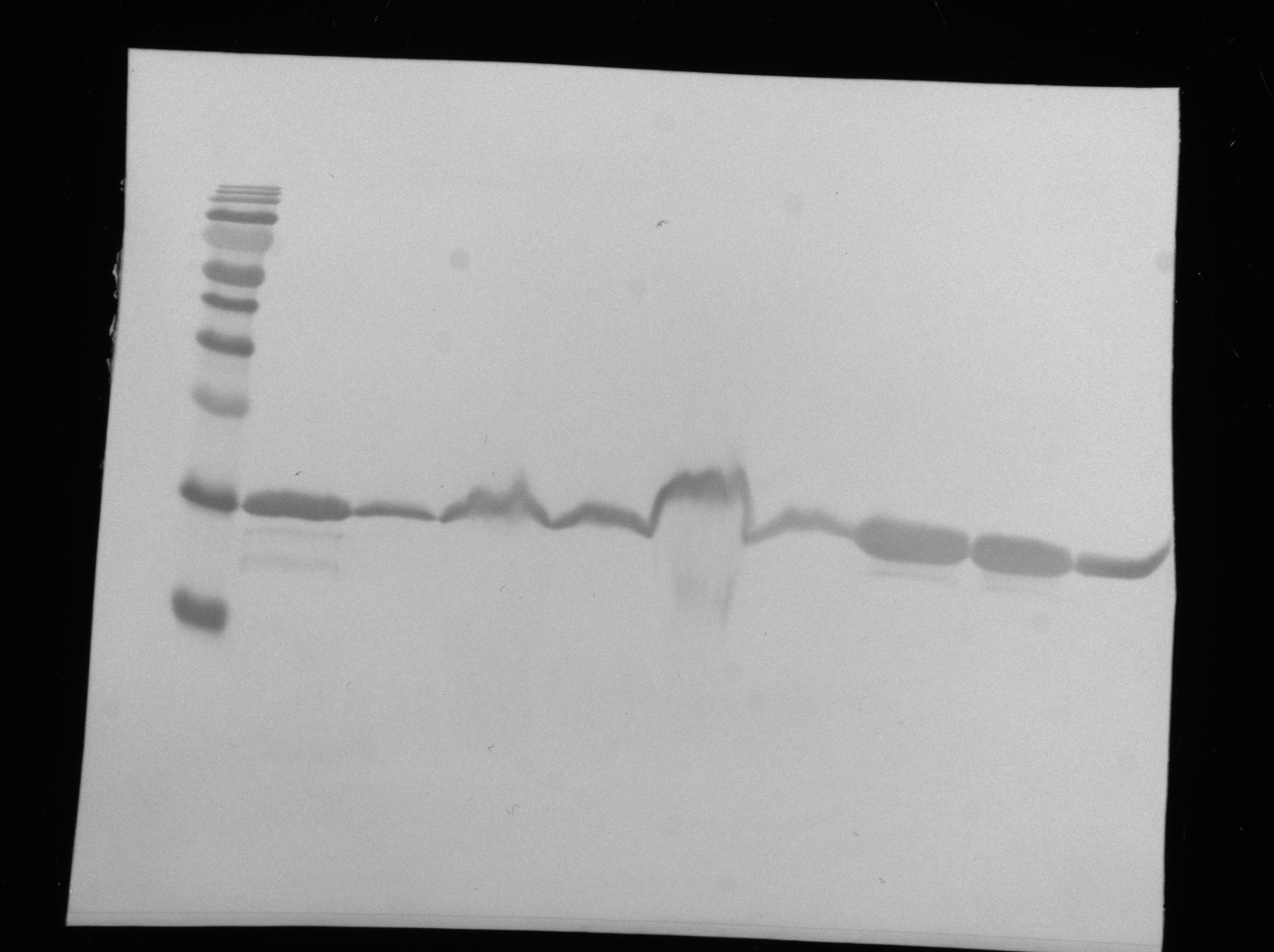

Supplement: Figure 2—figure supplement 2—source data 1. [file elife-101517-fig2-figsupp2-data1.zip › Figure 2–figure supplement 2-right panel.tif]

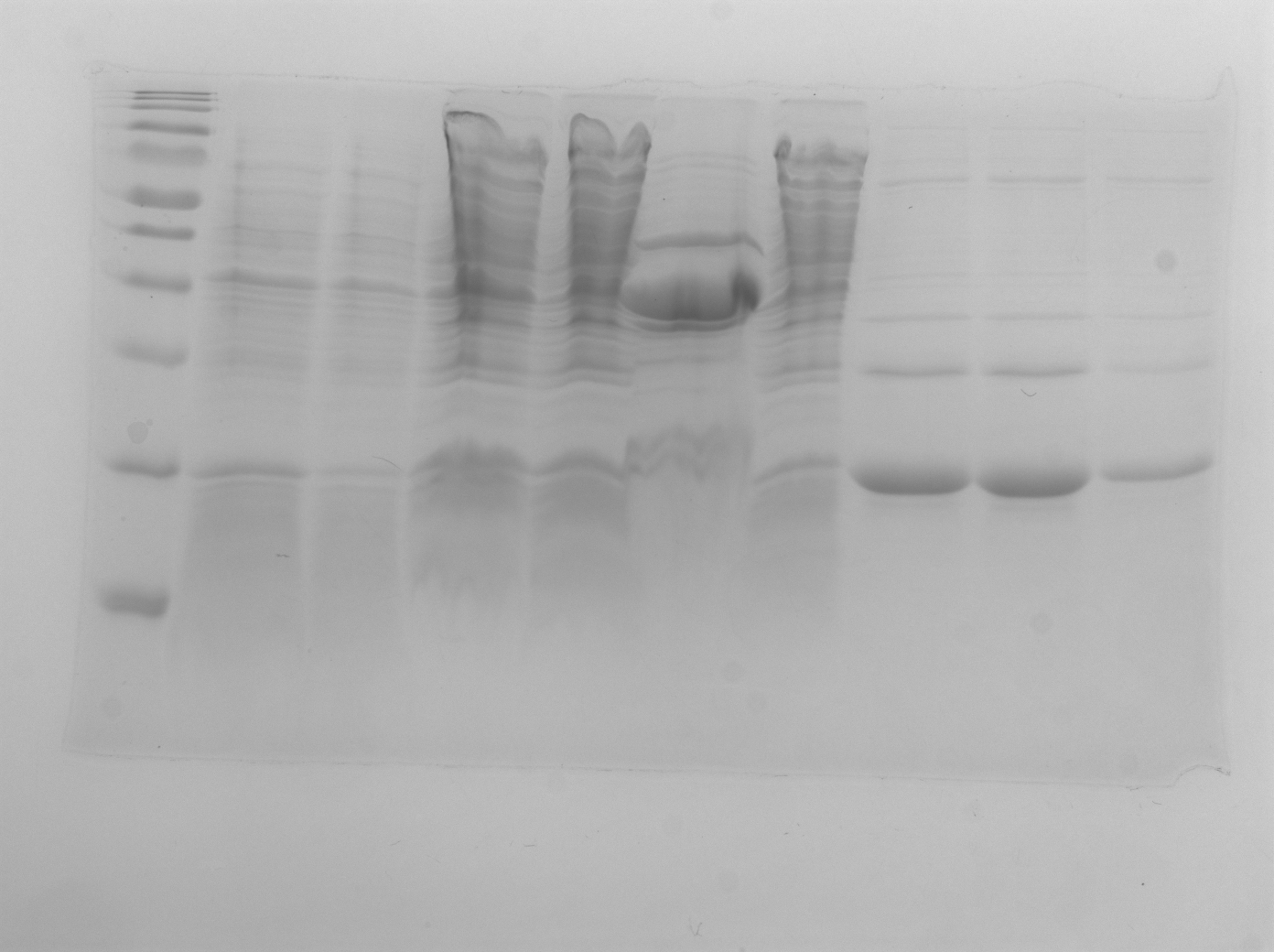

Supplement: Figure 2—figure supplement 2—source data 1. [file elife-101517-fig2-figsupp2-data1.zip › Figure 2–figure supplement 2-left panel.tif]

Figure 2—figure supplement 2—source data 1

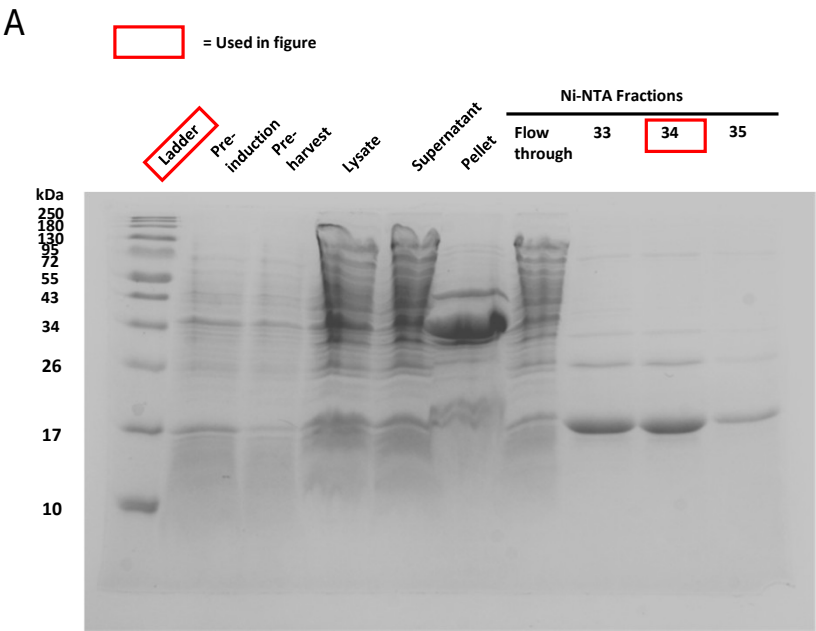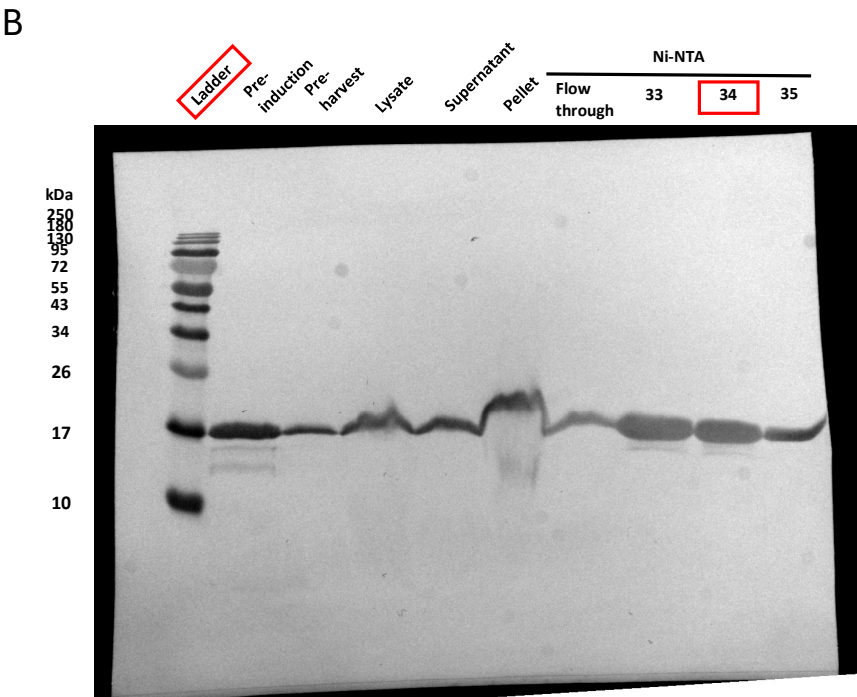

Supplement: Figure 2—figure supplement 2—source data 2. [file elife-101517-fig2-figsupp2-data2.zip › Figure 2–figure supplement 2-source data 1.pdf]

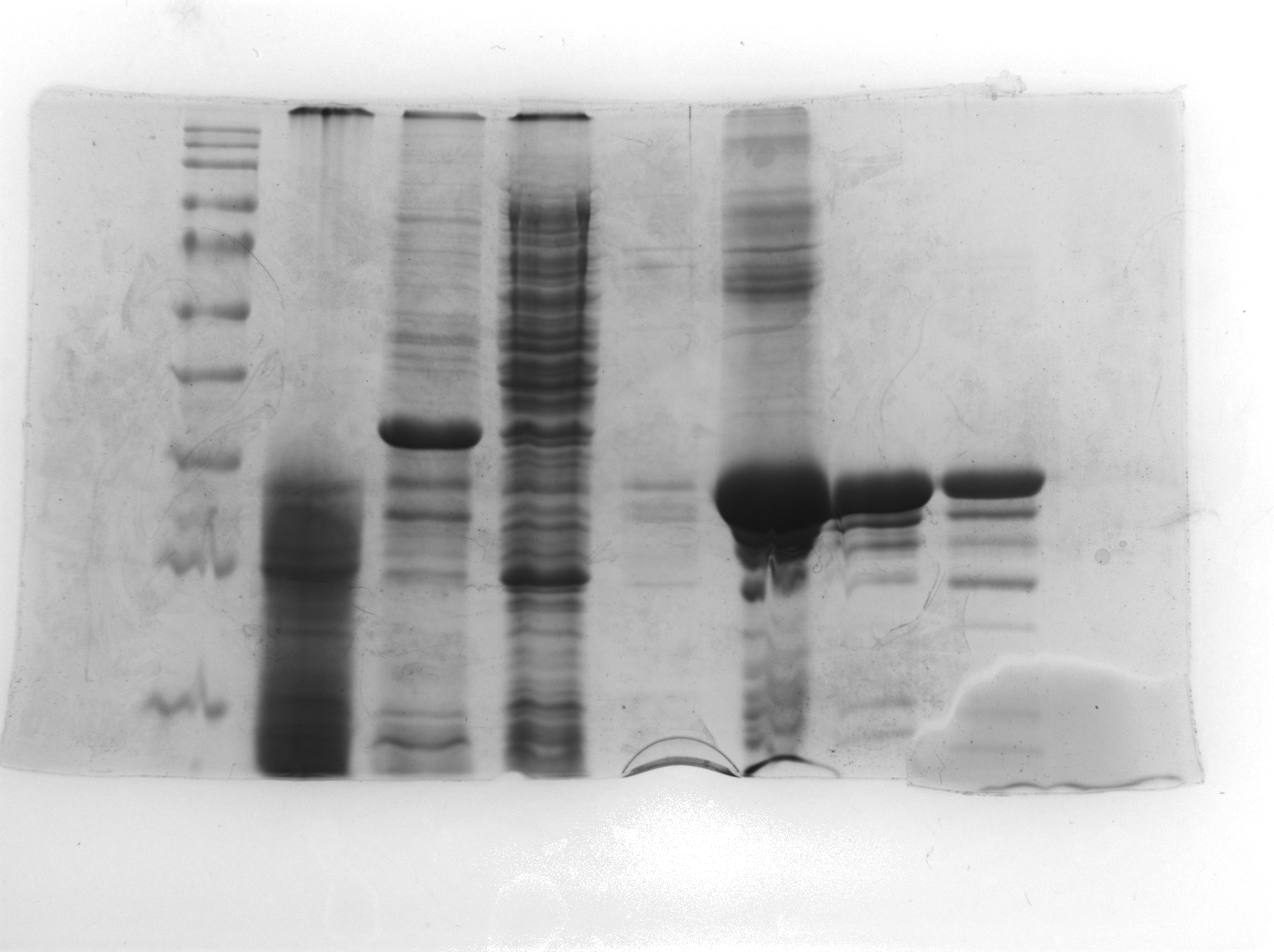

Supplement: Figure 4—figure supplement 1—source data 1. [file elife-101517-fig4-figsupp1-data1.zip › Figure 4-figure supplement 1-left panel.tif]

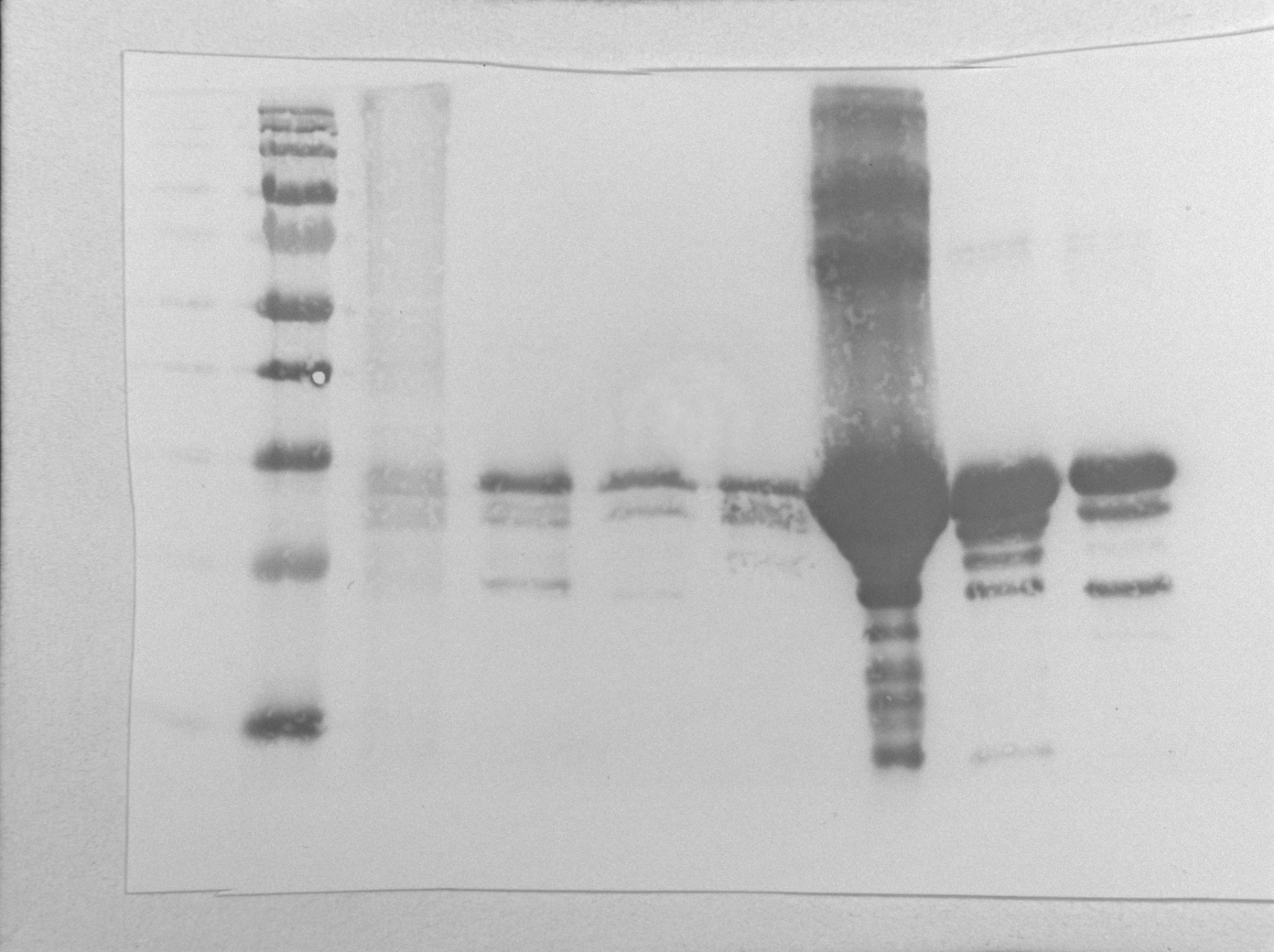

Supplement: Figure 4—figure supplement 1—source data 1. [file elife-101517-fig4-figsupp1-data1.zip › Figure 4-figure supplement 1-right panel.tif]

Figure 4—figure supplement 1—source data 1

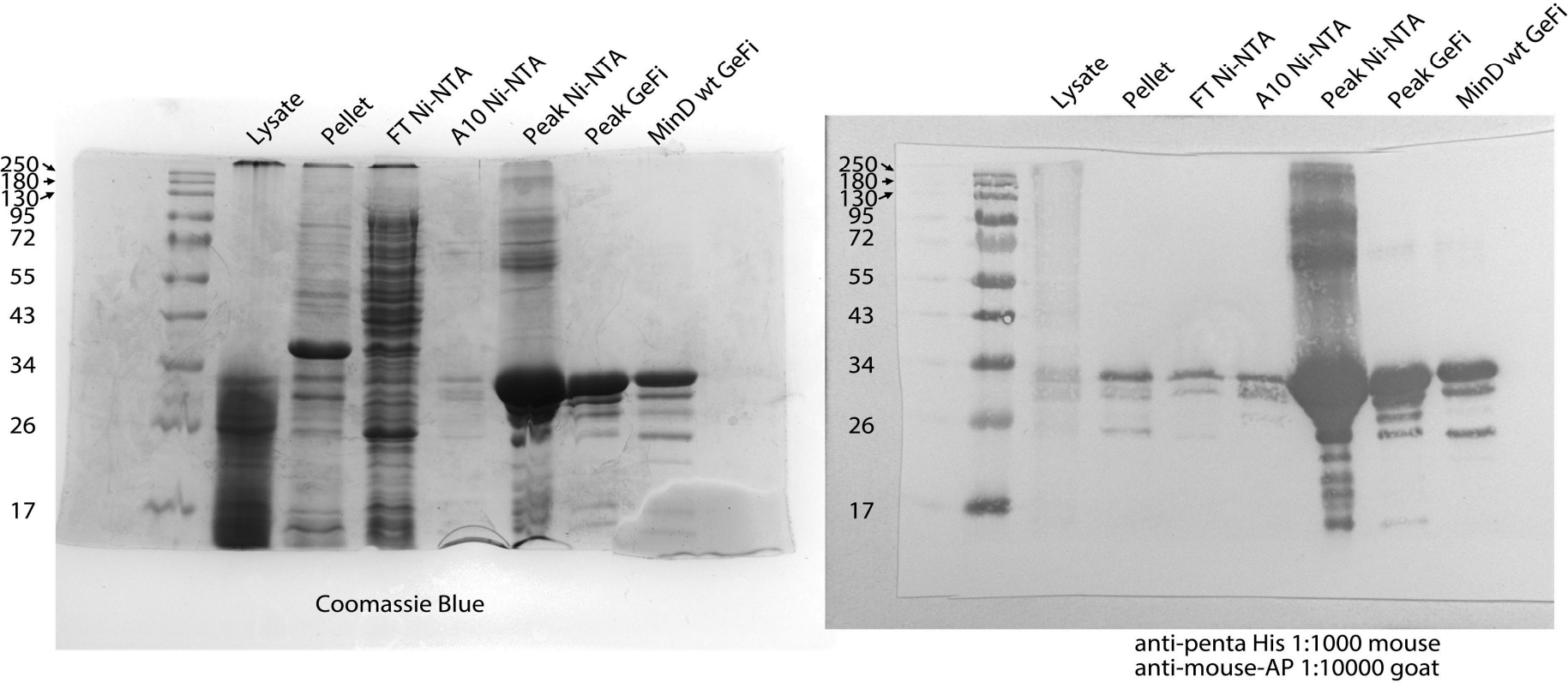

Supplement: Figure 4—figure supplement 1—source data 2. [file elife-101517-fig4-figsupp1-data2.zip › Figure 4–figure supplement 1-source data 1.pdf]

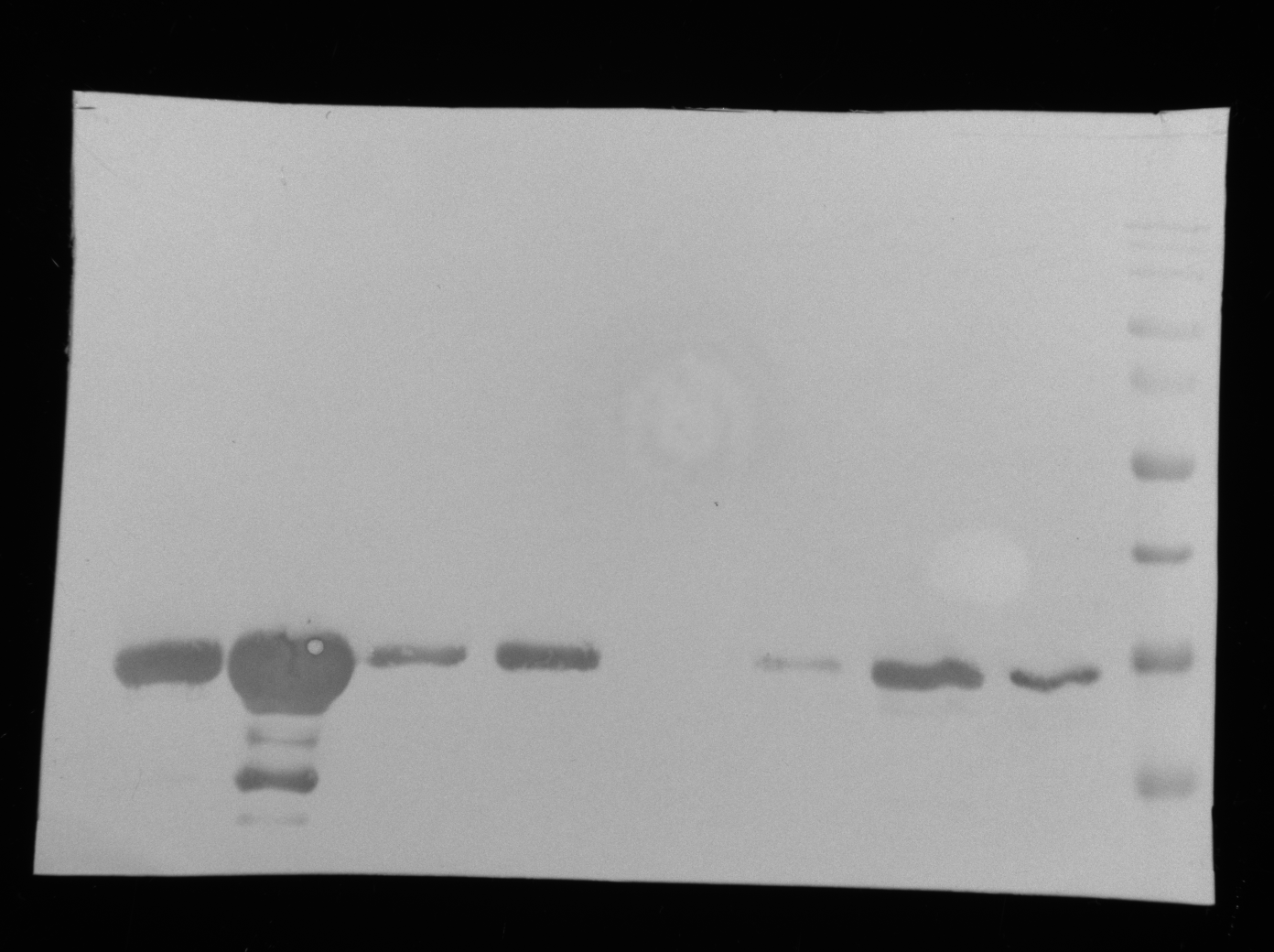

Supplement: Figure 4—figure supplement 2—source data 1. [file elife-101517-fig4-figsupp2-data1.zip › Figure 4-figure supplement 2-lower panel-mirrored.tif]

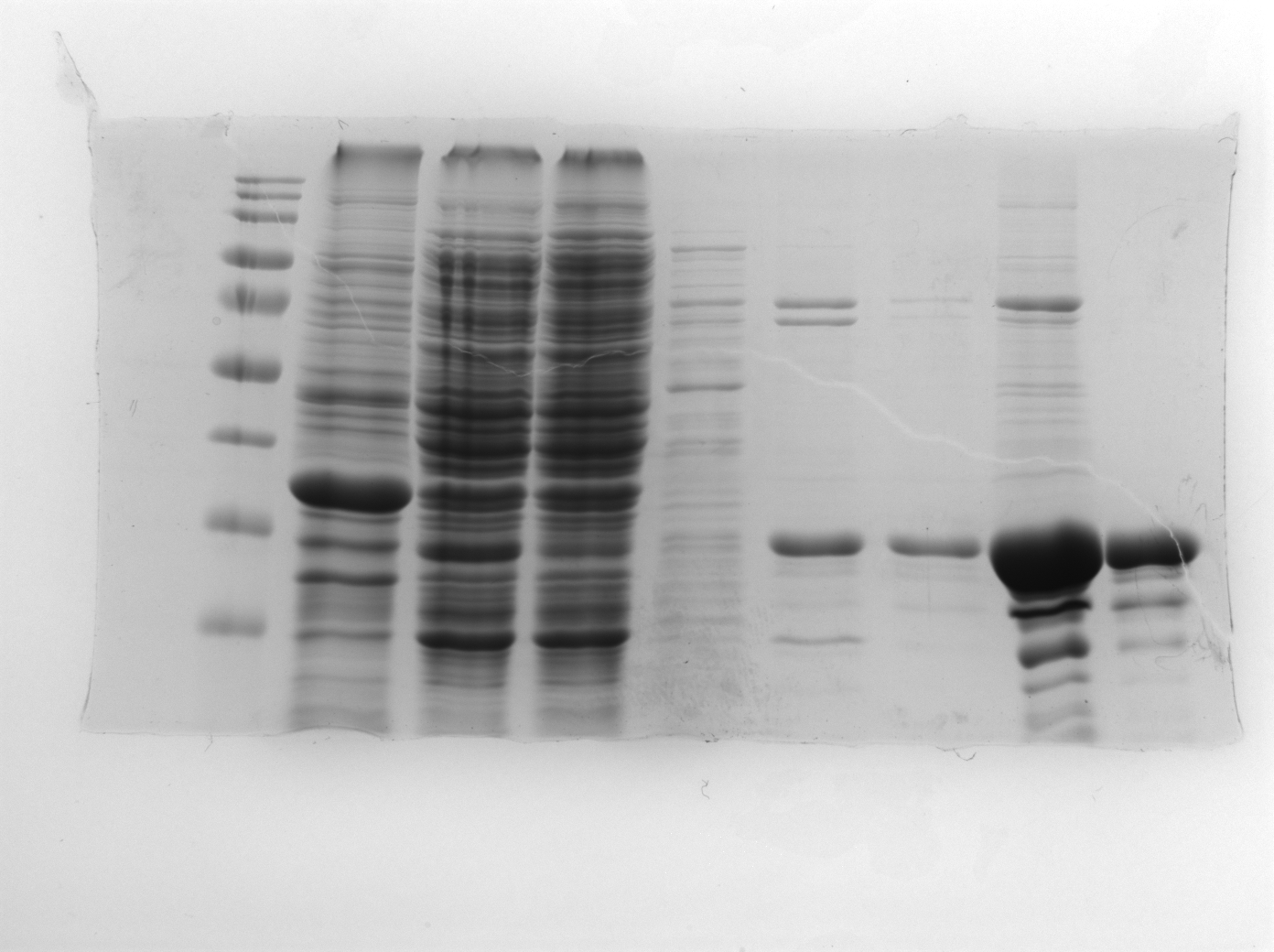

Supplement: Figure 4—figure supplement 2—source data 1. [file elife-101517-fig4-figsupp2-data1.zip › Figure 4-figure supplement 2-upper panel.tif]

Figure 4—figure supplement 2—source data 1

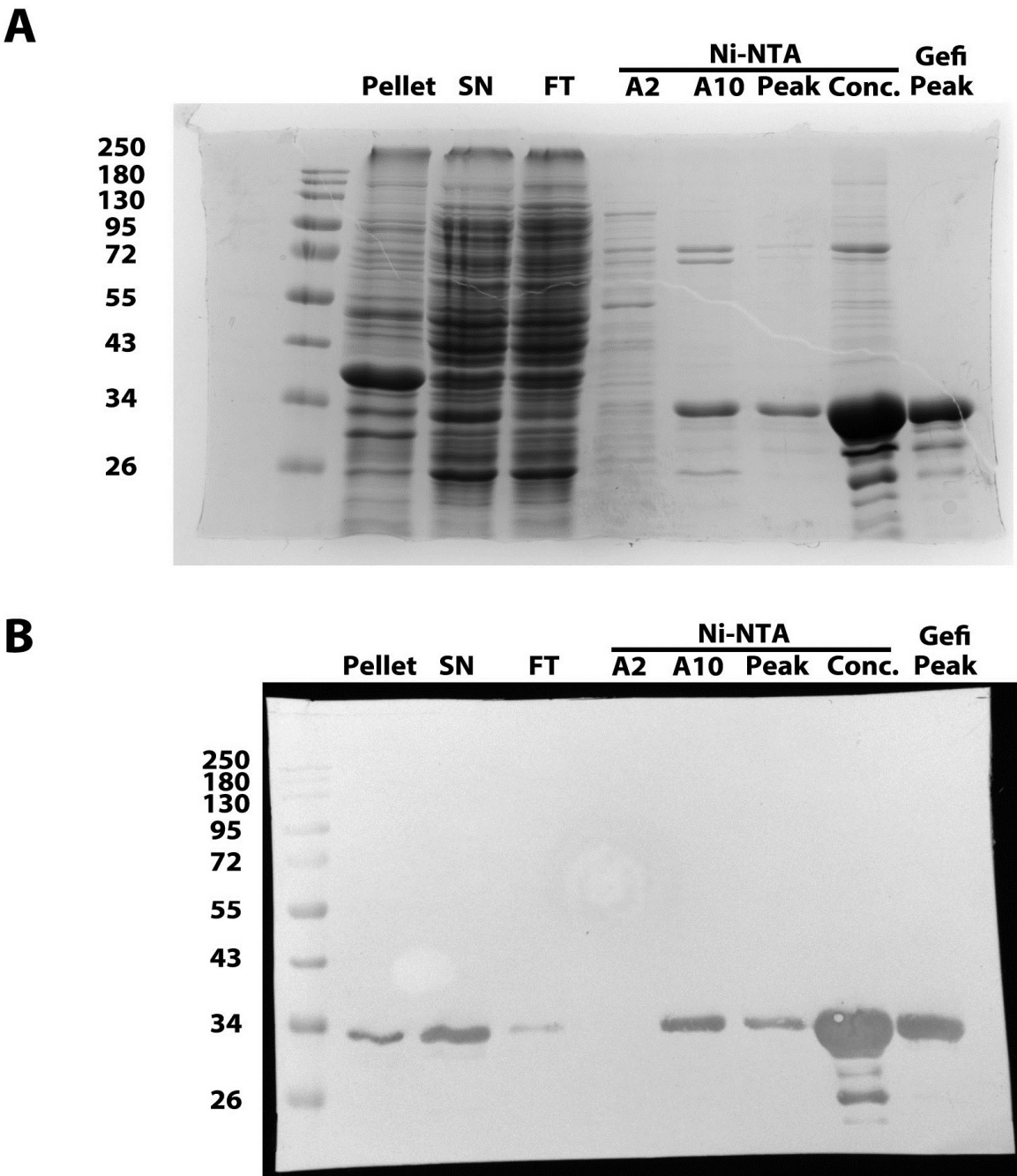

Supplement: Figure 4—figure supplement 2—source data 2. [file elife-101517-fig4-figsupp2-data2.zip › Figure 4–figure supplement 2-source data 1.pdf]

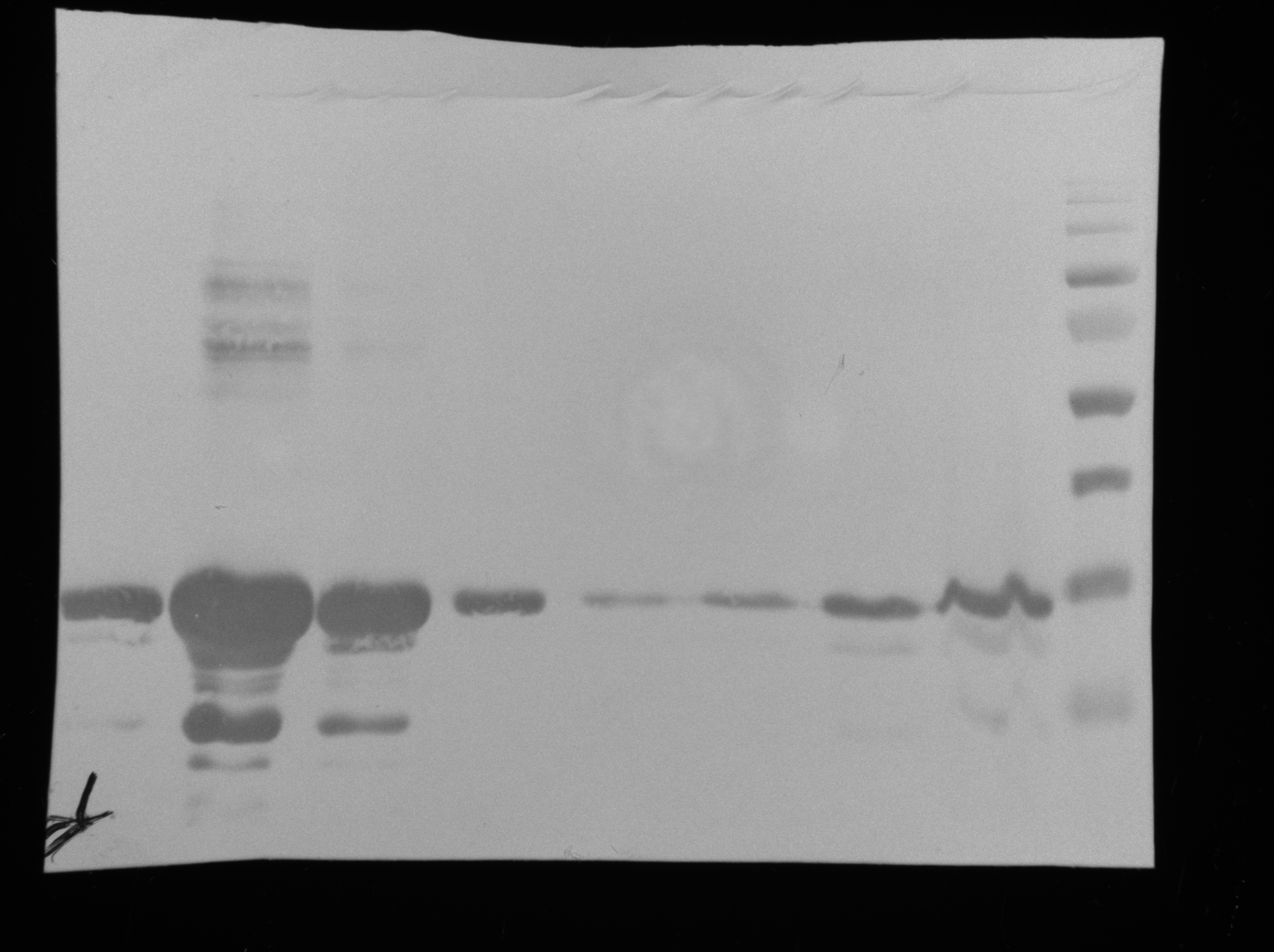

Supplement: Figure 4—figure supplement 3—source data 1. [file elife-101517-fig4-figsupp3-data1.zip › Figure 4-figure supplement 3-lower panel-mirrored.tif]

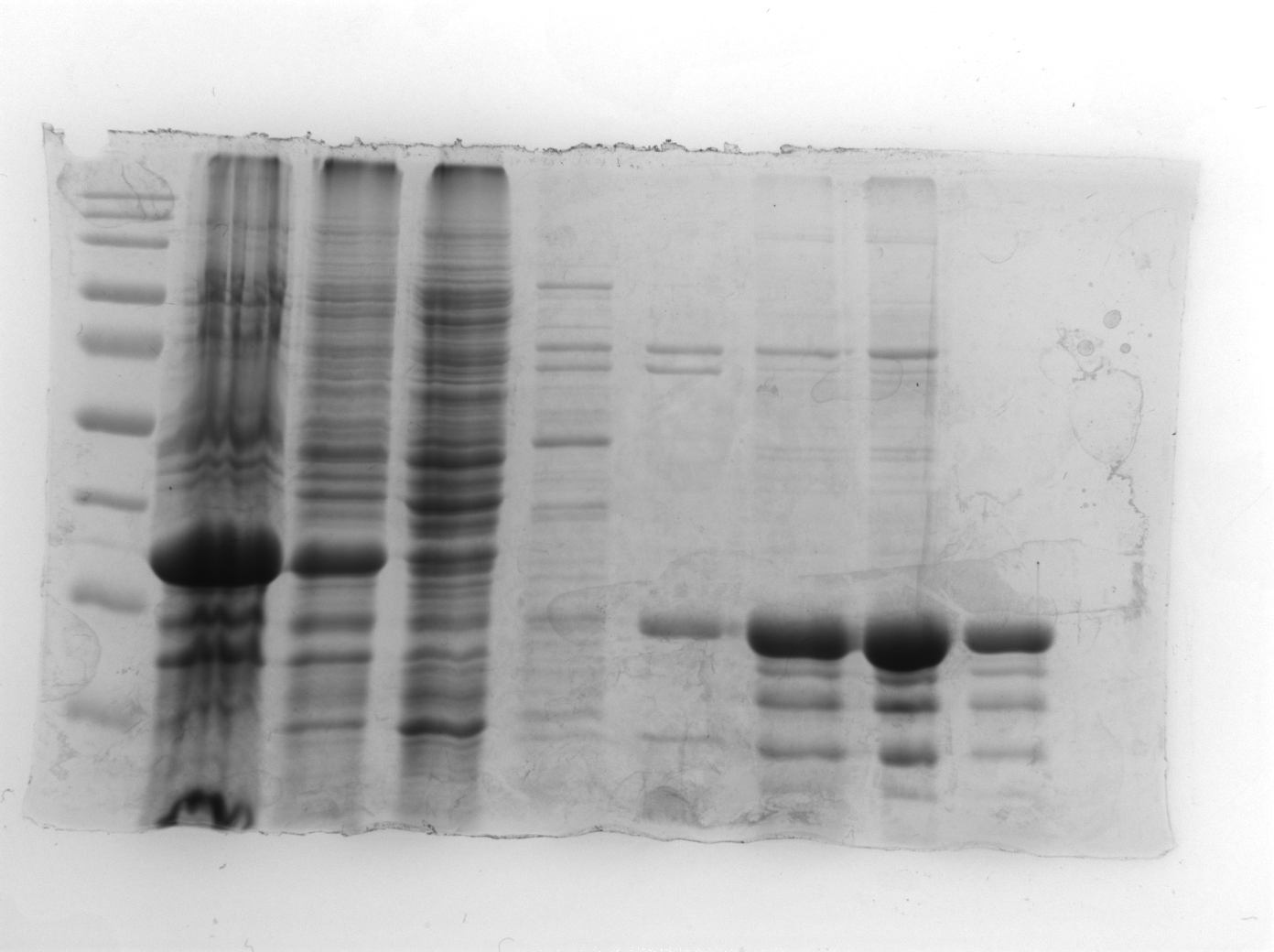

Supplement: Figure 4—figure supplement 3—source data 1. [file elife-101517-fig4-figsupp3-data1.zip › Figure 4-figure supplement 3-upper panel.tif]

Figure 4—figure supplement 3—source data 1

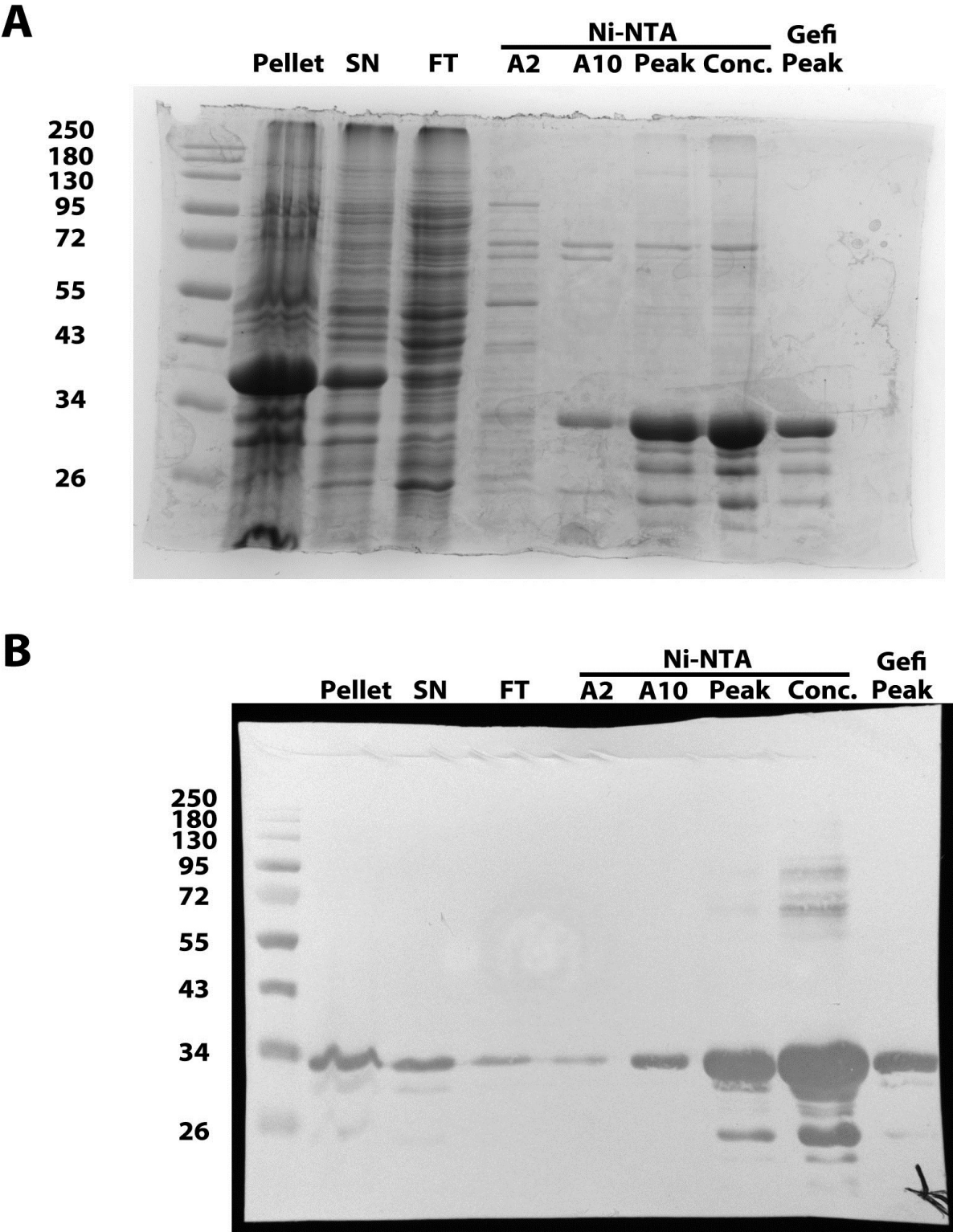

Supplement: Figure 4—figure supplement 3—source data 2. [file elife-101517-fig4-figsupp3-data2.zip › Figure 4–figure supplement 3-source data 1.pdf]

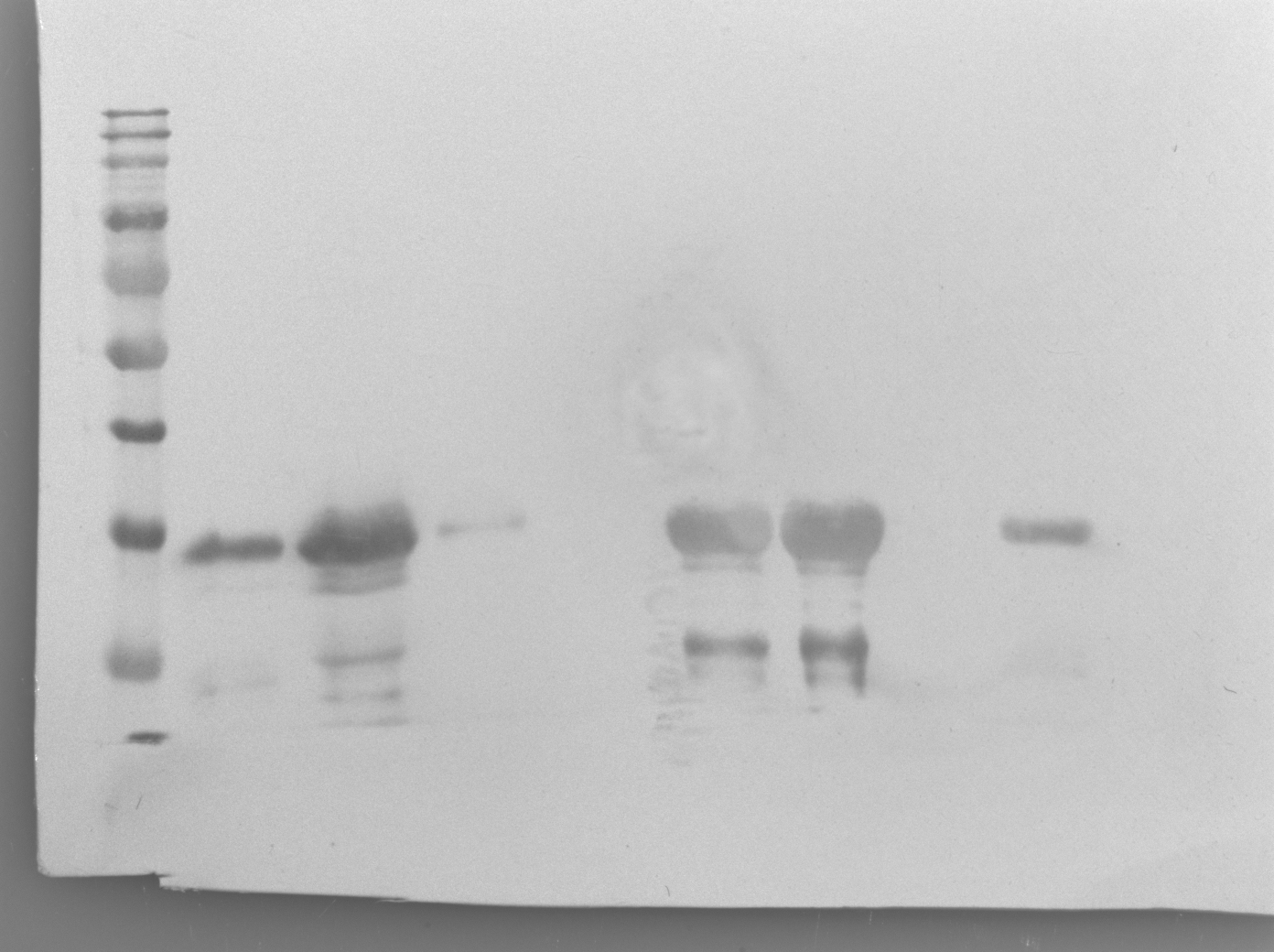

Supplement: Figure 4—figure supplement 4—source data 1. [file elife-101517-fig4-figsupp4-data1.zip › Figure 4-figure supplement 4-lower panel-mirrored.tif]

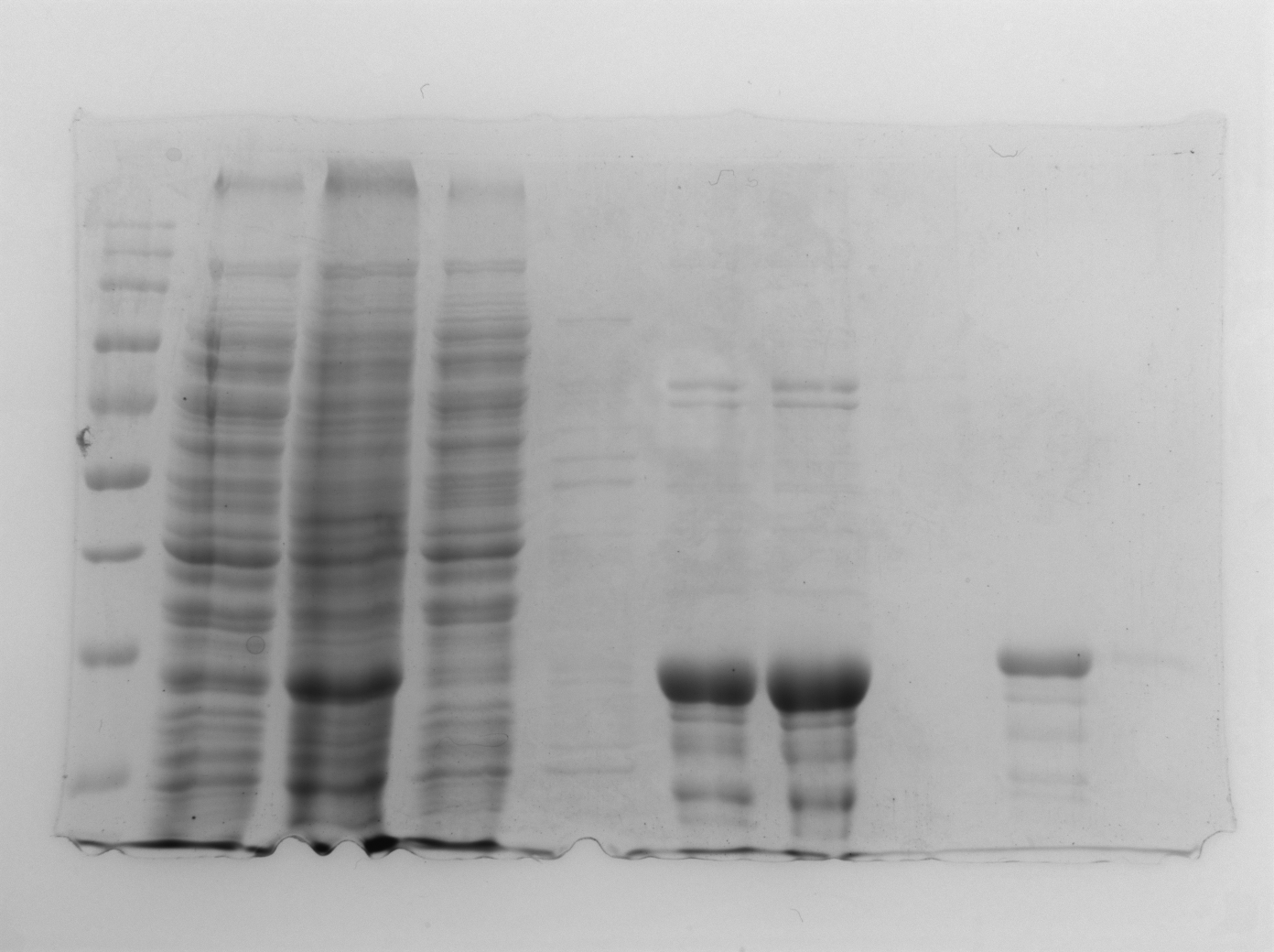

Supplement: Figure 4—figure supplement 4—source data 1. [file elife-101517-fig4-figsupp4-data1.zip › Figure 4-figure supplement 4-upper panel.tif]
