# Supplementary material for: Membrane binding controls the ATPase cycle and localization of MinD in Bacillus subtilis
Source: eLife. 2026 Jun 8;13:RP101517. doi: 10.7554/eLife.101517 (PMC13246001; doi:10.7554/eLife.101517)
Supplement: Figure 1—figure supplement 1—source data 2. [file elife-101517-fig1-figsupp1-data2.zip › Figure 1–figure supplement 1-source data 1.pdf]

Figure 1—figure supplement 1—source data 1

SDS-PAGE Gel

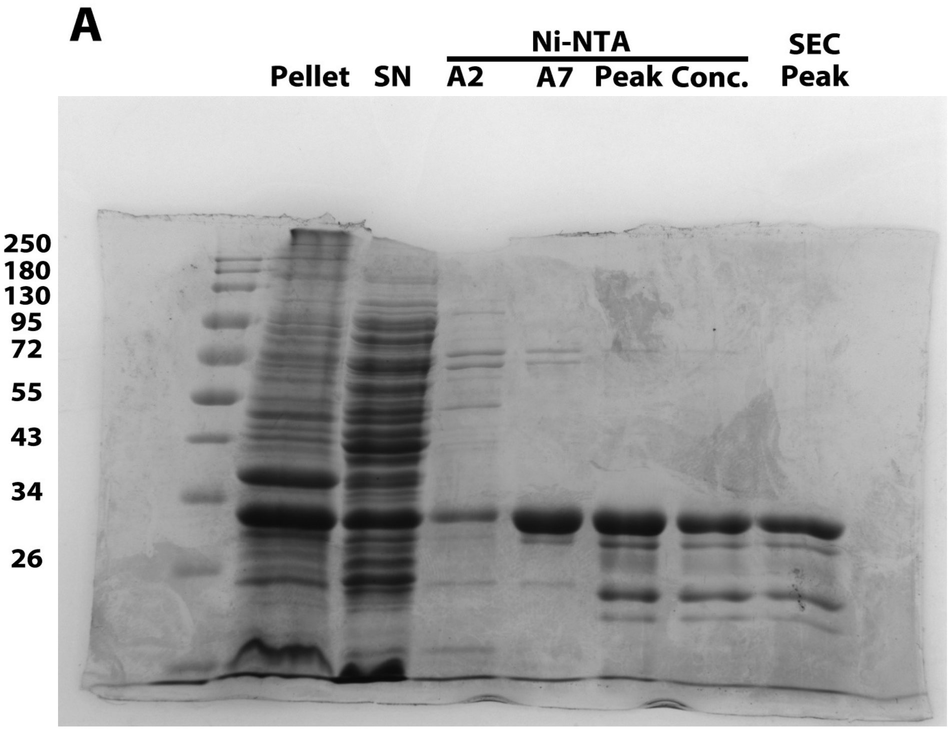

Western blot

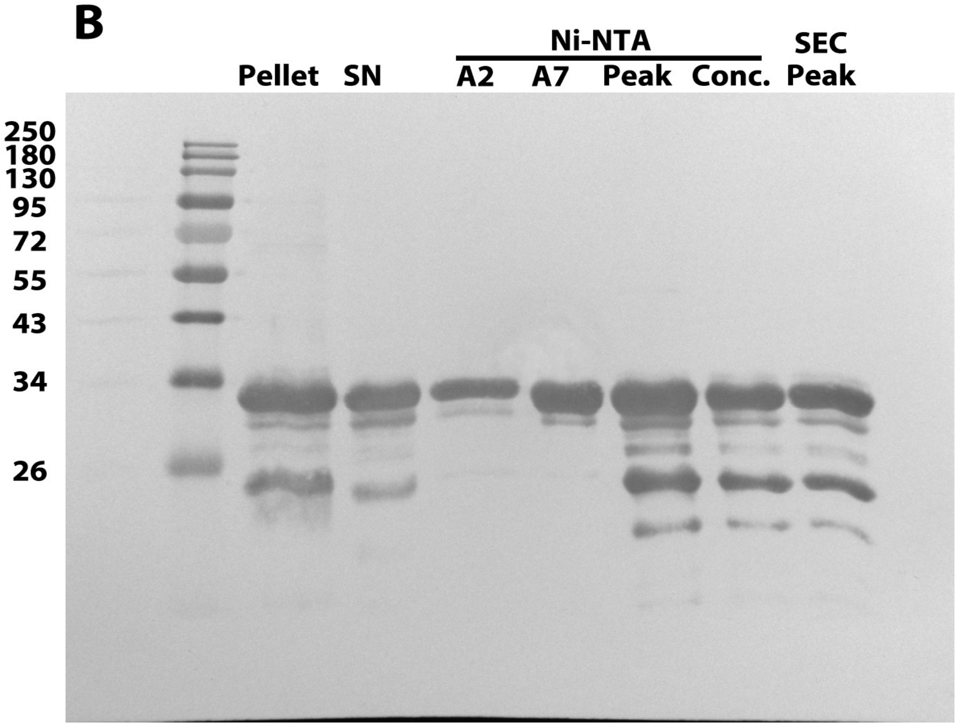

SN = Supernatant

Conc. = Concentrated sample after Amicon centrifugation

SEC Peak = Gel filtration / size exclusion peak
