# Supplementary material for: Membrane binding controls the ATPase cycle and localization of MinD in Bacillus subtilis
Source: eLife. 2026 Jun 8;13:RP101517. doi: 10.7554/eLife.101517 (PMC13246001; doi:10.7554/eLife.101517)
Supplement: Figure 4—figure supplement 4—source data 2. [file elife-101517-fig4-figsupp4-data2.zip › Figure 4–figure supplement 4-source data 1.pdf]

Figure 4—figure supplement 4—source data 1

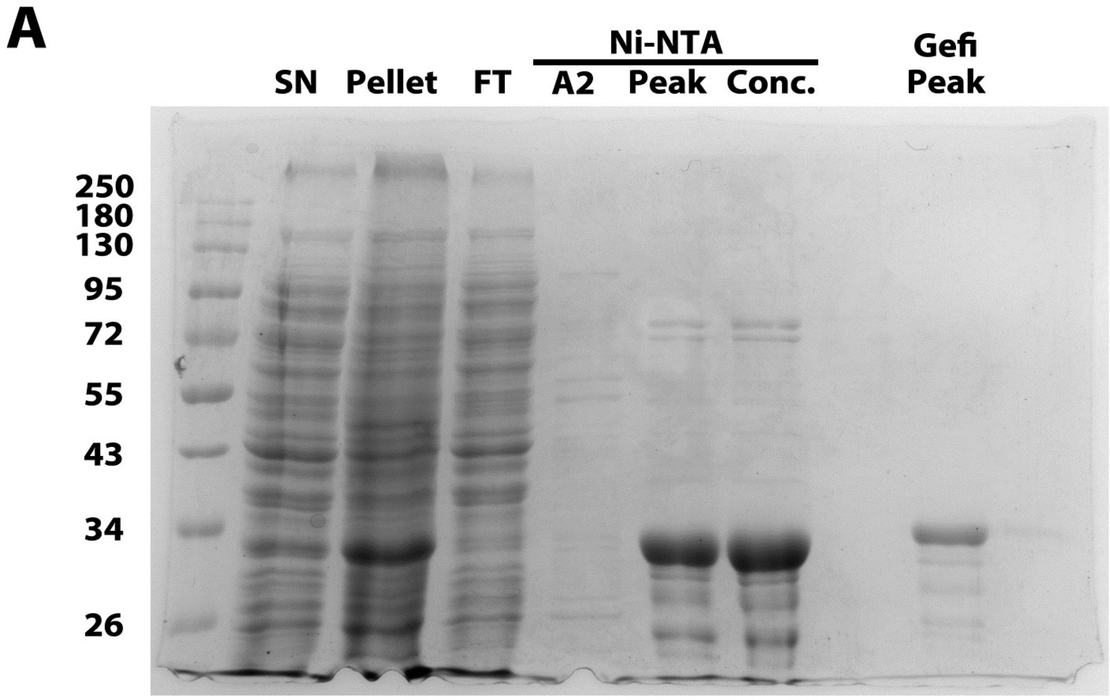

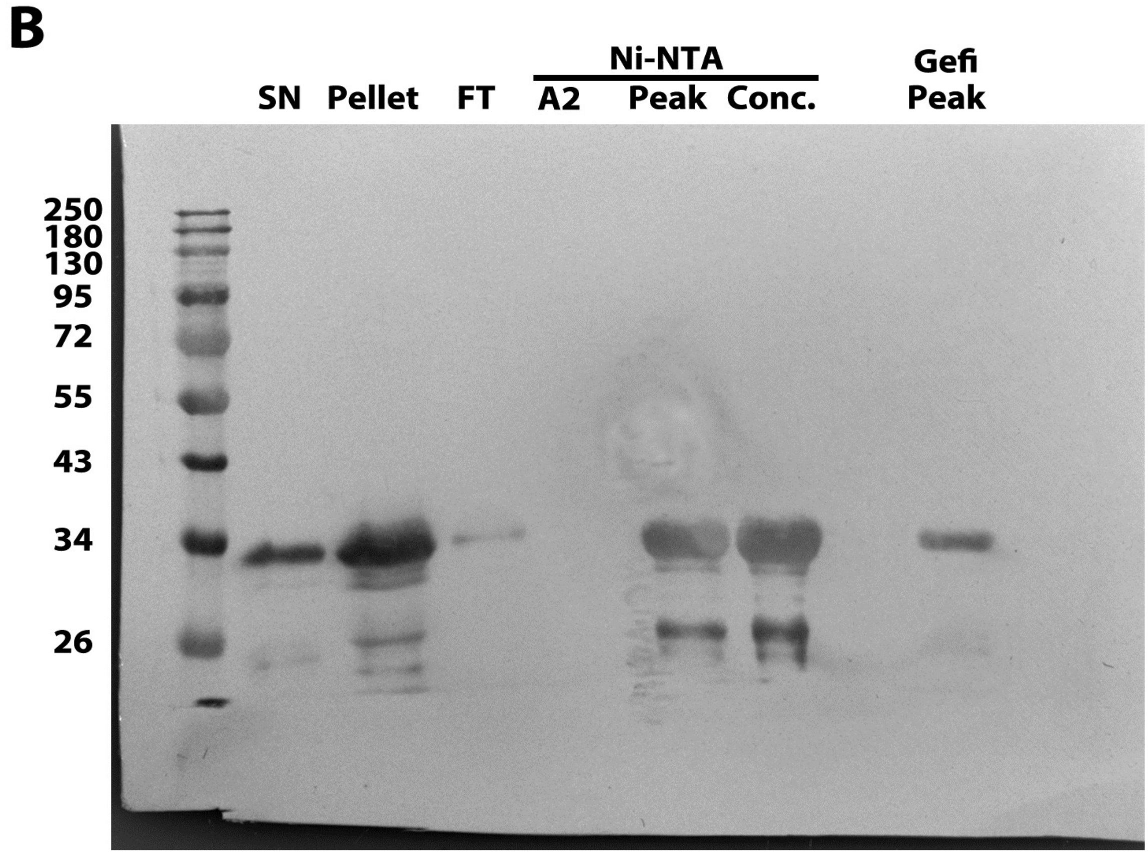

SN = Supernatant

FT = Flow through

Conc. = Concentrated sample after Amicon centrifugation

Gefi Peak = Gel filtration / size exclusion peak
