# Supplementary material for: Membrane binding controls the ATPase cycle and localization of MinD in Bacillus subtilis
Source: eLife. 2026 Jun 8;13:RP101517. doi: 10.7554/eLife.101517 (PMC13246001; doi:10.7554/eLife.101517)
Supplement: Peer review history

# Editor report: eLife Assessment

**DOI:** 10.7554/eLife.101517.4.sa0

**Authors:** Petra Anne Levin

**Affiliations:** Washington University in St. Louis United States

**Keywords:** Convincing, Important

This **important** study provides **convincing** data suggesting that subcellular localization of the spatial regulator of cell division, MinD, is an intrinsic feature of the protein's ability to associate with the membrane as both a dimer and a monomer. These findings distinguish the behavior of MinD in *B. subtilis* from its counterpart in *E. coli* and suggest that there is not a need to invoke additional localization factors. The reviewers felt that the revisions, particularly the additional experiments and changes to the text to make the experimental design and conclusions clearer, improve the quality of the manuscript.

# Referee report: Reviewer #1 (Public review)

**DOI:** 10.7554/eLife.101517.4.sa1

[Editor's note: this version has been assessed by the Reviewing Editor without further input from the original reviewers. The authors have addressed the comments raised in the previous round of review.]

Summary:

In this work the authors investigate the molecular dynamics of MinD, a component of the *Bacillus subtilis* Min system, in vitro and in vivo. In *Escherichia coli* the Min system is highly dynamic and displays rapid pole to pole oscillation whereby a time average minimum of the Min proteins at mid cell is established. However, in *B. subtilis*, this is not the case, and there is no MinE present. MinD in *B. subtilis* dynamically relocalizes from the poles to division sites, and binds to MinC and MinJ, which mediates its interaction with DivIVA. This paper reports biochemical characterization of *B. subtilis* MinD in vitro and dynamics of MinD variants in vivo, providing mechanistic insight into the mechanism of dynamic localization.

Strengths:

In the current study, the authors perform a detailed biochemical characterization of the in vitro ATPase activity of MinD and demonstrate that rapid hydrolysis is elicited by adding phospholipids. They further show using a collection of substitution mutants of MinD that both monomers and dimers bind to the membrane, and ATP occupancy changes the on and off rates. Identification, quantification, and tracking of discrete Halo-MinD populations was nicely done and showed that mutations in MinD alter dynamic localization, correlating with PL binding on and off rates in vitro.

In the revised manuscript, the authors now demonstrate localization and tracking data for minC and minJ deletion strains, which suggest that MinJ impacts MinD membrane cycling, but MinC does not. Additional in vitro work showed that the PDZ domain of MinJ modifies MinD ATP hydrolysis rates, and the authors propose that MinJ may promote MinD dimer formation.

Weaknesses of the revised version: No major weaknesses.

# Referee report: Reviewer #2 (Public review)

**DOI:** 10.7554/eLife.101517.4.sa2

Summary:

Feddersen & Bramkamp determined important characteristics of how MinD protein binds/dissociates to/from the membrane, and dimerizes in relation to its ATPase activity. The presented data clearly shows the differences in function of MinD homologs from *B. subtilis* and *E. coli*.

Strengths:

The work presents well-executed experiments that lead to interesting conclusions and a new model of how Min system works during *B. subtilis* mid-cell division. Importantly, this model is supported by in vitro characterization of well-chosen mutants in the functional domains of MinD. Outstandingly, most of the in vitro data are confirmed by single-molecule localization microscopy.

# Author comment: Author response

**DOI:** 10.7554/eLife.101517.4.sa3

**Authors:** Helge Feddersen, Charlotte Dyckmans, Marc Bramkamp

**Affiliations:** Kiel University Kiel Germany; Kiel University Kiel Germany; Kiel University Kiel Germany

The following is the authors’ response to the previous reviews

**Public Reviews:**

**Reviewer #1 (Public review):**

Summary:

In this work the authors investigate the molecular dynamics of MinD, a component of the *Bacillus subtilis* Min system, in vitro and in vivo. In *Escherichia coli* the Min system is highly dynamic and displays rapid pole to pole oscillation whereby a time average minimum of the Min proteins at mid cell is established. However, in *B. subtilis*, this is not the case, and there is no MinE present. MinD in *B. subtilis* dynamically relocalizes from the poles to division sites, and binds to MinC and MinJ, which mediates its interaction with DivIVA. This paper reports biochemical characterization of *B. subtilis* MinD in vitro and dynamics of MinD variants in vivo, providing mechanistic insight into the mechanism of dynamic localization.

Strengths:

In the current study, the authors perform a detailed biochemical characterization of the in vitro ATPase activity of MinD and demonstrate that rapid hydrolysis is elicited by adding phospholipids. They further show using a collection of substitution mutants of MinD that both monomers and dimers bind to the membrane, and ATP occupancy changes the on and off rates. Identification, quantification, and tracking of discrete Halo-MinD populations was nicely done and showed that mutations in MinD alter dynamic localization, correlating with PL binding on and off rates in vitro.

In the revised manuscript, the authors now demonstrate localization and tracking data for minC and minJ deletion strains, which suggest that MinJ impacts MinD membrane cycling, but MinC does not. Additional in vitro work showed that the PDZ domain of MinJ modifies MinD ATP hydrolysis rates, and the authors propose that MinJ may promote MinD dimer formation.

Weaknesses of the revised version: No major weaknesses.

We thank this reviewer for the positive evaluation of our manuscript and the precise summary of our findings.

**Reviewer #2 (Public review):**

Summary:

Feddersen & Bramkamp determined important characteristics of how MinD protein binds/dissociates to/from the membrane, and dimerizes in relation to its ATPase activity. The presented data clearly shows the differences in function of MinD homologs from *B. subtilis* and *E. coli*.

Strengths:

The work presents well-executed experiments that lead to interesting conclusions and a new model of how Min system works during *B. subtilis* mid-cell division. Importantly, this model is supported by in vitro characterization of well-chosen mutants in the functional domains of MinD. Outstandingly, most of the in vitro data are confirmed by single-molecule localization microscopy.

Weaknesses:

The authors immobilized liposomes, for which they used *E. coli* total lipids, to measure ATPase activity and liposome association and dissociation of *B. subtilis* MinD. For these experiments would be more suitable to use *B. subtilis* total lipids as more biologically relevant data could be gained.

Although the work is in detail and nicely compares the function of *B. subtilis* Min system with *E. coli* Min system, it lacks the comparison of the Min system function in other rod-shaped Gram-positive bacteria. I would suggest including in the Discussion the complexity of other Min systems. Especially, this complexity is seen in other rod-shaped and spore formers such as Clostridial species in which one of these Min systems or both are present, an oscillating *E. coli* Min system type and more static as in *B. subtilis*.

Comments on revisions:

I'm satisfied with the authors response to my private recommendation points. However, I thought that they would also respond to my points mentioned in Public Review part, weaknesses as shown above and update the revised version accordingly.

We are very grateful to the reviewer for the positive comments and fully agree with the points raised. Due to the overall length of the manuscript, we initially omitted a discussion of the complexity of the Min system in certain Firmicutes. However, we agree that this aspect should be considered. Accordingly, we have now added a dedicated paragraph to the Discussion section addressing this point.

We also agree that investigating different lipid compositions, including native membranes from *Bacillus subtilis*, represents a logical next step to further elucidate the influence of lipids on the MinD activity cycle. However, we consider this to constitute a separate project and therefore beyond the scope of the present study.

**Recommendations for the authors:**

**Reviewing Editors:**

Some minor corrections are requested-the addition of a bit more details about the complexity of Min systems in other bacteria in particular to the discussion as suggested by Reviewer 2 would be very much appreciated.

We thank the editors for their positive assessment and the clear recommendations. We have now added a dedicated paragraph to the Discussion section addressing the complexity of the Min system in *Clostridioides*.

**Reviewer #1 (Recommendations for the authors):**

The following corrections are requested:

Abstract - Line 29 - Remove the word "solely" from this statement of the abstract. It would be wise to not be so rigid for a biological system that is only partially characterized and to allow for the possibility that biological factors, including local concentrations and/or other molecules, may yet be discovered to impact MinD activation under certain conditions.

We agree and have amended the text to avoid a to restrictive statement.

Line 38 - Remove "do not require any unknown protein component" for the reason stated above. Currently, the experiments recapitulate activation suggesting the membrane binding and release controls dynamics without additional factors. This allows for the possibility that biological factors may yet be shown to impact MinD activation under certain conditions.

We agree and have change the text.

Discussion - Line 526 - Thermus thermophilus is misspelt.

Corrected.
